# Ontogeny of the spinal cord dorsal horn

**DOI:** 10.1126/science.adx5781

**Published:** 2026-01-08

**Authors:** Robert Brian Roome, Archana Yadav, Lydia Flores, Amrit K. Puarr, Diana Nardini, Alexander Richardson, Ronald R. Waclaw, Ruth M. Arkell, Vilas Menon, Jane E. Johnson, Ariel J. Levine

**Affiliations:** 1 Spinal Circuits and Plasticity Unit, National Institute of Neurological Disorders and Stroke, Bethesda, MD, USA; 2 Center for Translational and Computational Neuroimmunology, Department of Neurology, Columbia University Irving Medical Center, New York, NY, USA; 3 Department of Neuroscience, UT Southwestern Medical Center, Dallas, TX, USA; 4 Maternal-foetal Precision Health Laboratory, John Curtin School of Medical Research, The Australian National University, Canberra, ACT, Australia; 5 Experimental Hematology and Cancer Biology, Cincinnati Children’s Hospital Medical Center, Cincinnati, OH, USA; 6 Department of Pediatrics, University of Cincinnati College of Medicine, Cincinnati, OH, USA; 7 Department of Pharmacology, UT Southwestern Medical Center, Dallas, TX, USA

## Abstract

The dorsal horn of the mammalian spinal cord is organized into laminae where each layer is populated by different neuron types, has unique circuit connections, and plays specialized roles in behavior. An outstanding question is how this organization emerges during development from an apparently homogeneous pool of neural progenitors. Here, we show that mouse dorsal neurons are diversified by time, with families of related cell types born as temporal cohorts, and by a spatial-molecular gradient that specifies individual cell types. Excitatory neurons settle into a chronotopic arrangement that transforms their progressive birthdates into anatomical order and is required to establish proper laminae. We identified essential ontogenetic principles that shape dorsal progenitors into the diverse cell types and structure that subserve sensorimotor function.

The dorsal horn of the spinal cord is crucial for some of the most important neural tasks of vertebrate animals, from processing pain and touch signals from the body to implementing sensorimotor control. It relies on a highly structured organization in which dozens of molecularly discrete excitatory and inhibitory neuronal cell types are arrayed within anatomical layers, or laminae (1, 2). Each cell type has a characteristic transcriptional profile and “families” of closely related cell types are highly conserved across mammalian species (3–5). Each cell type also has a characteristic spatial distribution that is a strong predictor of its synaptic inputs and function, as the sensory and descending pathways that target the dorsal horn each terminate in stereotyped laminar zones (1, 2, 4, 6–9). For example, neurons in lamina I and II process pain, itch, and temperature (6, 10–12), those in lamina III and IV process light touch (6, 8, 13, 14), and those in lamina V and VI process the sense of body-position and contribute to motor control (4, 15–20). Overall, the arrangement of diverse cell types in laminae is a hallmark of the dorsal horn and the framework that supports normal behavioral function.

Despite our detailed knowledge of these cell types and structure, how all this diversity and anatomical organization comes to be remains to be fully elucidated. This is in sharp contrast with development of the ventral horn – a textbook example of developmental logic (21, 22). The ventral horn is mainly formed during an early phase of spinal neurogenesis, when secreted ligands spatially pattern the progenitors into discrete lineage domains that each give rise to a “cardinal class” of interneurons (23–27) . Then, as neurogenesis proceeds, a small group of late dorsal progenitors lose their early lineage restriction, expand dramatically, and form a large common pool of pdL progenitors (progenitors of dorsal late) that generate the inhibitory (dILA; dorsal interneuron late) and excitatory (dILB) neurons that comprise the dorsal spinal cord (23, 24, 28–30). How does this seemingly homogeneous population of progenitors generate the highly diverse and highly structured cellular organization of the dorsal horn?

Here, we examined mouse dorsal horn development from neurogenesis until the establishment of laminar structure using neuronal birthdating, transcriptional profiling, spatial transcriptomics, and mouse mutant analysis. We found that the families of the mature dorsal horn neuron types emerged as progressive waves of dIL neurons during late spinal neurogenesis. The excitatory families then settled into a chronotopic order, with progressively born families settling into adjacent lamina. To test the hypothesis that dorsal excitatory neurons establish the laminar structure of the dorsal horn, we genetically eliminated dorsal excitatory, dorsal inhibitory, or sensory neurons and found that dorsal excitatory neurons are crucial for the proper anatomical organization of the dorsal horn. We also discovered a spatial gradient of Zic transcription factors in dorsal progenitors and post-mitotic dIL neurons that sub-divided each family into more refined cell types, generating an array of approximately 150 molecularly discrete types. Together, this work uncovered the underlying developmental mechanisms that transform neural progenitors into the dorsal horn of the spinal cord, establishing the neural substrates for sensorimotor control.

## A transcriptional atlas of the late embryonic dorsal horn

Our understanding of cell type organization and development has shown recent advances due to the development of single cell profiling methods. We and others have applied these methods to study spinal cord biology in many different contexts; however, there is a persistent gap in our knowledge: the crucial period in which dorsal spinal neurons differentiate from relatively generic immature neurons into the diverse cell types of the dorsal horn. Prior work has shown that unsupervised transcriptional clustering of spinal neural progenitors does not separate cells into the expected lineage domains, and, instead, supervised “knowledge matrices” have been used to analyze the early cardinal classes (31, 32). This challenge is even greater for the late dILA and dILB classes that arise from common progenitors (23, 24, 28, 30). We therefore sought to characterize the full complement of newly born dorsal neurons at the transitional stage when they might retain ontogenic signatures while gaining mature neuronal signatures. Describing this period in detail should provide insights into the processes of neuronal diversification, how cell types relate to each other, and laminar development.

**Fig. 1:**
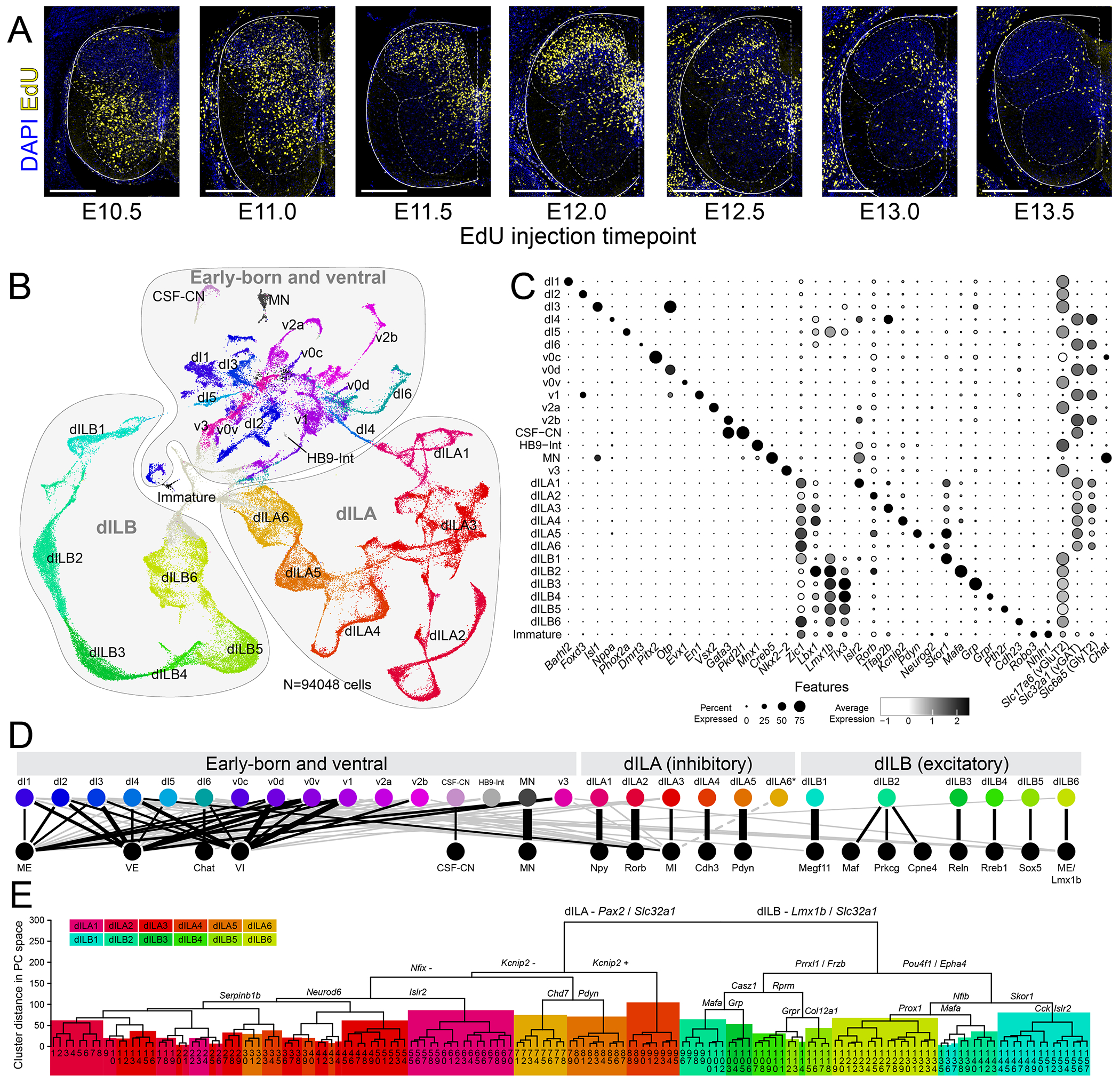
A single-cell transcriptomic atlas of the mouse spinal cord after neurogenesis. (**A**) Neuronal birthdate labeling in spinal cord sections from E16.5 embryos that received an EdU pulse (yellow) to mark dividing cells at the indicated time points between E10.5 and E13.5, with DAPI staining (blue). (**B**) UMAP plot of single-cell RNA-seq data taken from E14.5 and E16.5 wild-type mouse embryos showing all neurons, and annotated by Group (1 = Early-born and ventral, 2 = dILA, 3 = dILB) and by neuronal classes – including six dILA and six dILB families, each shown in a distinct color. (**C**) Dot plot showing gene expression in each neuron class. Dot size indicates the percent of each cluster expressing each gene and dot shade indicates the average expression level of each gene in cells of each cluster. (**D**) Diagram illustrating the relationships between embryonic neuron families and postnatal/adult neuron classes from Russ et al. (4). Line thickness is inversely proportional to centroid-centroid distance in PC space, with the strongest relationships emphasized with black (versus gray) lines. Asterisk indicates that the relationship between dILA6 to the postnatal class “MI” has been inferred by comparing refined neuron annotations (see Figs S7, S8). (**E**) A dendrogram of centroid-centroid distance in PC space for refined neuron types among all dIL neurons. Each terminal branch number corresponds to named cell-types in Fig. S4. Selected branch points are annotated with top differentially expressed genes. Where branches encompass large parts of dIL families they are shaded with colors representing each dIL family (see legend). Numbers: (A,B) EdU birthdating data was derived from 79 E16.5 mouse embryos given EdU on E10.5 (n=13 from four litters), E11.0 (n=10 from three litters), E11.5 (n=15 from four litters), E12.0 (n=7 from three litters), E12.5 (n=12 from four litters), E13.0 (n=10 from three litters) or E13.5 (n=12 from four litters). (C-F) Single-cell RNA-seq data was derived from fifteen litters of mouse embryos (n=3 E14.5, n=12 E16.5), and of which data from N=94048 neurons were extracted. Scale bars: (A) 250 μm

To examine the full complement of dorsal horn neurons, we charted a detailed time course of spinal neurogenesis. EdU (5-ethynyl-2’-deoxyuridine), which is incorporated into the DNA of dividing cells, was injected into female mice with timed pregnancies at half-day time points from embryonic day 10.5 (E10.5) through E13.5, covering the general period of spinal cord neurogenesis. At E16.5, when the dorsal horn’s laminar structure is evident (33), we analyzed the distribution of EdU+ neurons (Fig. 1A). Nearly all neurons that settled in the dorsal horn were born between E11.0 and E13.0, and neurogenesis was essentially complete by E13.5, consistent with previous work (24–27, 34, 35. Based on these results, we generated a single-cell RNA-sequencing (RNA-seq) dataset of spinal cords at E14.5 and E16.5 (Fig. S1A). We profiled 195,146 spinal cord cells (Fig. S1, Tables S1 and S2) and obtained 94,048 neurons (Fig. 1B). Hierarchical clustering was used to reveal multiple levels of spinal neuron organization. At the highest level, neurons were divided into three major groups that each accounted for approximately one third of the dataset: Group 1 was enriched for *Zfhx3*, *Meis2*, and *Nrp2*; Group 2 was enriched for *Pax2*, *Gad2*, and *Neurod6*; and Group 3 was enriched for *Lmx1b*, *Ebf2*, and *Tlx3* (Fig. S2).

Group 1 was composed of the cardinal spinal cord neuron types clearly identified by classical marker genes including dI1 (*Barhl2*), dI2 (*Foxd3+Slc17a6*), dI3 (*Isl1*+*Otp*), dI4 (inhibitory *Lbx1*+ without *Dmrt3*), dI5 (*Phox2a*), dI6 (*Dmrt3*), v0c (*Pitx2*), v0d (presumed; *Otp* without *En1* or *Isl1*), v0v (*Evx1*), v1 (*Foxd3* + *En1*), v2a (*Vsx2*), v2b (*Gata3*), and v3 (*Nkx2-2*) interneurons, motoneurons (*Isl1*+*Mnx1+Creb5*) , as well as Hb9 interneurons (*Mnx1*+*Slc17a6*) and cerebrospinal fluid contacting neurons (*Gata3*+*Pkd2l1*). Semi-supervised clustering yielded the full set of expected spinal lineage classes, which we identified by expression of classic markers (Fig. 1B,C, Tables S3 and S4) and by correlation with a well-annotated atlas of the period of spinal neurogenesis (E9.5 – E13.5) by Delile and colleagues (31).

Groups 2 and 3 represented the putative dILA neurons (Group 2) and dILB neurons (Group 3), based on the expression of general embryonic dorsal markers (shared with putative dI4 and dI5 classes), modest residual expression of early post-mitotic markers (suggesting recent neurogenesis), later-born temporal transcription factors, as well as the expression of known markers of mature dorsal neurons, the region where dILA/B neurons reside (4, 23, 24, 31) (Fig. 1B,C). Given that dILA and dILB neurons share some gene expression with dI4 and dI5 neurons, we sought distinguishing characteristics between these late-born and early-born classes to justify a point of demarcation in our dataset. Exploratory analysis revealed that the transcription factor Zic1 was expressed in putative dILA and dILB neurons, but not dI4 and dI5 neurons. Zic1 is known to be expressed in the dorsal progenitor zone throughout neurogenesis and to appear in post-mitotic neurons around E11.5, which aligns with the emergence of the first dIL neurons (35, 36). Immunofluorescence of newly born neurons in the sub-ventricular zone confirmed this pattern. We found that Zic1 was expressed in a few scattered dI4 neurons and no dI5 neurons at E10.5, but was expressed in the majority of dILA and dILB neurons at E11.5 (Fig. S3). Accordingly, we used *Zic1* expression to delineate the boundaries between dI4-dILA and dI5-dILB clusters (see Methods).

The internal organization of dILA and dILB neurons remained to be fully investigated, limiting our understanding of how dIL neurons diversify into the dozens of cell types in the mature dorsal horn. There are many potential ways to divide the Group 2 dILA and Group 3 dILB populations; we opted for a practical clustering-based approach (see Methods). This yielded six classes within each Group that we designated dILA1 through dILA6 and dILB1 through dILB6 (Fig. 1B,C and Fig. S4,). Each of these classes were robust and well-separated, with high silhouette width values (that measure the similarity distance between cells of the same cluster versus cells of nearby clusters), very low LISI scores (Local Inverse Simpson’s Index that measures the degree of intermixing between cells of different clusters), and high discrimination accuracy and precision with a machine learning (random forest) classifier (Fig. S4, S5A). Comparison of these dILA and dILB classes with a harmonized transcriptomic atlas of postnatal spinal neurons (4) revealed that each dILA or dILB class correlated strongly with a single “family” or a small group of related families (Fig. 1D, Table S5), with the exception of dILA6 which did not have a clear postnatal family counterpart. To determine whether dILA6 neurons are present at postnatal stages or instead represent a transient fate, we performed in situ hybridization (ISH) for a dILA6 marker gene set (coincident *Tfap2a*, *Galr1*, and *Neurog2*) at E16.5 and postnatal day 6, and found that small groups of triple positive dILA6 cells were found adjacent to the dorsal midline in each case, suggesting that dILA6 represents a bona fide class of spinal neurons (Fig. S6). Overall, these data reveal that, at E14.5 and E16.5, young dILA and dILB neurons are already well organized as families, the intermediate level of cell type hierarchy.

To examine the most refined level of cell type organization hierarchy, that of specific cell types, we sub-clustered each family and found approximately 150 molecularly distinguishable clusters (Fig. 1E, Figs. S4, S5B, S7 and Tables S6–S8). To determine whether refined dIL neuron types were segmentally restricted, we compared the cell type proportions in samples derived from cervical, thoracic, and lumbar segments. We found that refined dIL neuron-types were largely present at all segments, with a few modest differences (Fig. S4). We next compared these refined dIL clusters to the refined postnatal neuron types (4), and found strong mutually specific relationships between them, allowing us to define the embryonic origin of each previously identified mature population in the dorsal horn (Fig. S8, Tables S9–S10). This analysis also revealed a specific association for dILA6 which corresponded to the refined class of Inhib-15 cells (which was also *Tfap2a*^+^/*Galr1*^+^/ *Neurog2*^+^), rather than sharing a broad correspondence to an entire postnatal family.

This work provided a comprehensive characterization of embryonic spinal neurons post-neurogenesis, allowing us to link the transcriptomic profiles of progenitor lineage domains with their differentiated progeny. In particular, we found that both the family and refined type organization of the mature dorsal horn are already present shortly after neurogenesis.

## Temporal diversification of embryonic dILA and dILB families

During the early wave of spinal neurogenesis, morphogen-directed spatial patterning is the dominant driver that creates the set of canonical lineage classes (37). However, to the best of our knowledge, spatially restricted domains within the pdL progenitor pool have not been found, raising the possibility that a distinct logic governs the development of the late-born dorsal neurons. Based on analogy with other laminated neural structures including the vertebrate retina, cortical plate, and tectum, we hypothesized that temporal patterning may govern the cell type diversification and anatomical layering of the dorsal horn (38–41).

**Fig. 2:**
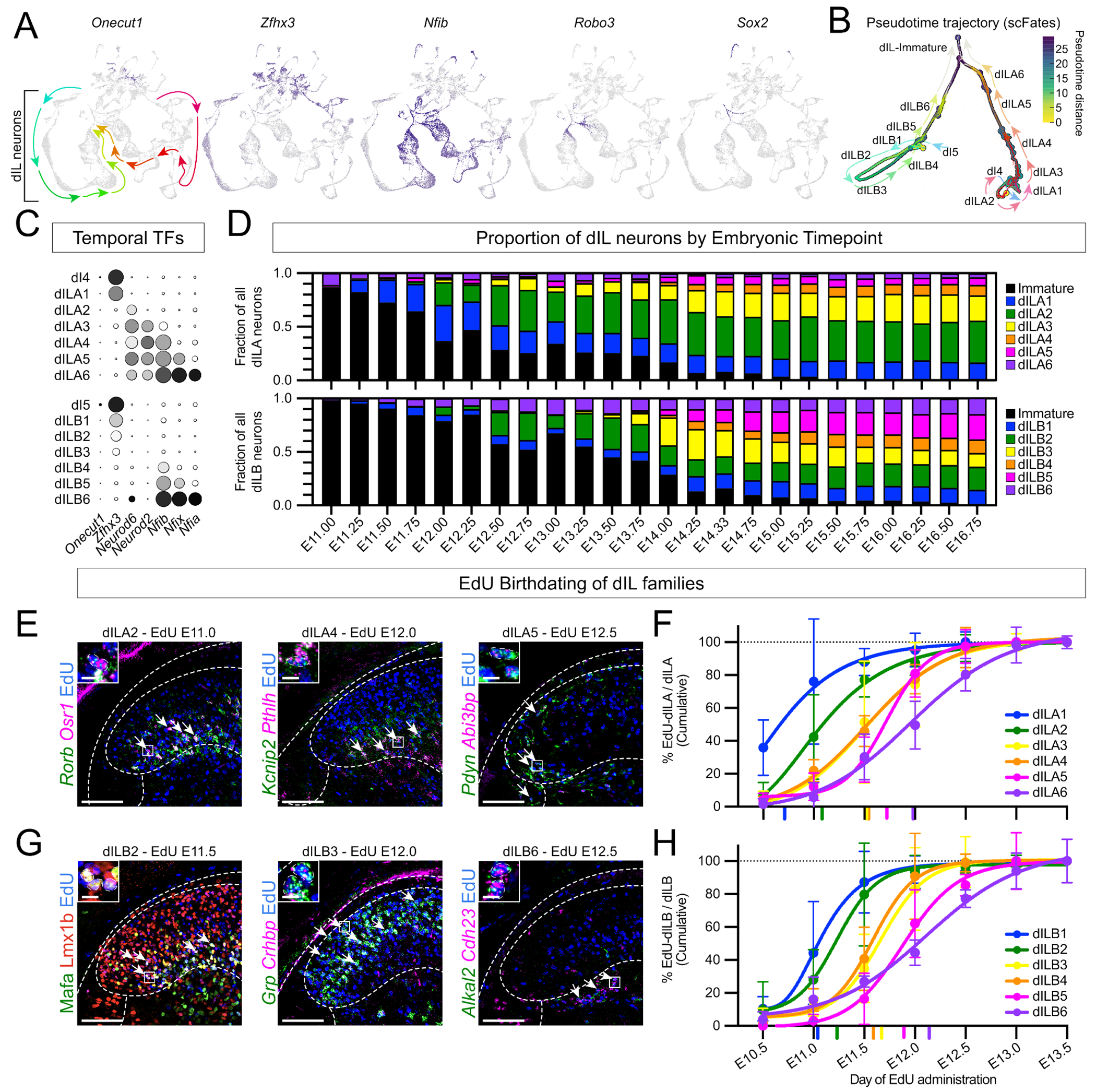
dIL families represent temporal cohorts. (**A**) UMAP feature plots of gene expression of each indicated gene in single-cell RNA-seq data, including temporal transcription factors (*Onecut1, Zfhx3, Nfib*), a marker of migrating and commissural neurons (*Robo3*) and a marker of neural progenitors (*Sox2*). Predicted birthdate trajectories for each family are indicated by correspondingly colored arrows on the UMAP plot indicating *Onecut1* expression. **(B)** Scatter plot showing spinal neurons in pseudotime space (scFates), with the pseudotime trajectory drawn in the background. Pseudotime values along the trajectory are colored per the legend. Neurons in (A) and (B) are colored as in Fig. 1 and are indicated by text and correspondingly colored arrows. (**C**) Dot plot of temporal transcription factors among dIL families, with dot size representing the percent of cells in each type expressing the gene and shade representing average expression. (**D**) Composition over time of dILA families (top) and dILB families (bottom), together with immature dILA/B neurons (black) in single-nucleus RNA-seq data from precisely-timed mouse embryo dissections (49). **(E-H)** EdU birthdating of dILA and dILB families. [E and G Examples of dILA (E) and dILB (G) family neurons (indicated by marker RNA or protein in green and red) co-labeled with EdU (blue) injected at the indicated time points]. White arrows indicate examples of dIL family subtypes co-labeled with EdU. Dashed white lines indicate approximate anatomical boundaries of the spinal cord, including the pial outer boundary, the gray/white boundary, and the superficial dorsal horn. Boxes indicate the field of view magnified in the inset. (F,H) Cumulative percent of each dILA (F) or dILB (H) family co-labeling with EdU pulsed at the indicated embryonic time point (x-axis) showing mean +/− standard deviation for all litters at each time point, connected by sigmoidal fit lines for each family. The imputed 50% cumulative birthdating time point for each family is indicated by a colored vertical line beneath the x-axis. Numbers: (A-C) Single-cell RNA-seq data is the same as Fig. 1B (see legend). (D) Enriched spinal cord neuron single-nucleus RNA-seq data (N=349,306 neurons) were retrieved from Qiu et al., (49) and refined cell type labels were transferred from our data (Fig. 1B,E, Figs. S4, and S7); from this, 74,764 dILA and 71,834 dILB neurons were analyzed across 24 time points. (E-H) EdU birthdating data were derived from 35 E16.5 mouse litters from a separate cohort to Fig. 1A, and data from individual embryos per litter were averaged. Pregnant mothers were given EdU on E10.5 (n=3-5 litters), E11.0 (n=4 litters), E11.5 (n=5 litters), E12.0 (n=5 litters), E12.5 (n=4-5 litters), E13.0 (n=5 litters) or E13.5 (n=2-6 litters). Scale bars: [(E) and (G)] 250 μm, 25 μm for insets. Statistics: See Methods; Table S12.

We first noted that dILA and dILB families were organized as streams in a UMAP (uniform manifold approximation and projection) plot, with one end closest to early-born Zfhx3+ dI4 and dI5 counterparts (34) and the other end closest to Robo3+ immature/migrating neurons (42–44) and Sox2+ progenitors (45) (Fig. 2A), suggestive of continuous temporal variation. A complementary dimensionality reduction and visualization approach, PHATE, revealed a similar pattern (Fig. S9) (46). Such inter-family relationships were further supported by the observation that the rare mistakes made by a classifier trained on family identity were between families that were adjacent in UMAP and PHATE plots (Fig. S5). These exploratory findings prompted us to test whether families are organized temporally, which we did using four independent approaches.

First, to test whether the dILA and dILB families were organized along transcriptional “pseudotime” trajectories, we used scFates, a probabilistic trajectory learning method that can be applied directly to single cell gene expression count data (47, 48). Using immature cells as the bifurcating root and the early born dI4/5 neurons included as temporal references, we found that dILA and dILB families each formed a continuous trajectory from dILA1 through dILA6 and from dILB1 through dILB6 (Figs. 2B and S9, Table S11).

Second, we assessed the expression of the Onecut, Zfhx, Nfi, and Neurod temporal transcription factors in each dILA and dILB family (Fig. 2C). The Onecut genes, that often mark the earliest-born cell types, were not present in the dIL families, but we did observe a smooth progression of Zfhx (mid early-born), Neurod (mid late-born), and Nfi (latest-born) genes from dILA1/B1 through dILA6/B6. Therefore, both the inhibitory and excitatory families of the dorsal horn vary by a combinatorial code of temporal transcription factors.

Third, we reasoned that molecularly distinct dILA and dILB families should appear in a progressive order if they are born successively. Recent work from Qiu et al. provided a large embryonic single cell atlas with high temporal resolution, with embryos staged and sorted on based on refined anatomical criteria into quarter-day time points (49). We extracted spinal cord neurons from this dataset, used label transfer to assign family identity, and then analyzed family composition over time. Among dILA families, the main order was dILA1 > dILA2 > dILA3 > dIL4 and dILA5, while dILA6 was detected throughout (Fig. 2D). Among dILB families, the main order was dILB1 > dILB2 > dILB3 > dILB5 > dILB4, with dILB6 detected throughout (Fig. 2D). The apparent early appearance of dILA6 and dILB6 likely reflects the similarity between late-born neurons and the ongoing presence of immature neurons which could reduce the accuracy of label transfer. Overall, these data further support the conclusion that dILA and dILB families represent temporal cohorts.

Fourth, we assessed dILA and dILB birthdates using timed EdU administration over half-day intervals combined with expression of family marker genes, or subsets thereof. This method measures birthdate more directly, though it provides slightly lower temporal resolution due to imprecision in timing of conception relative to injection time and experimental analysis (50, 51). Among dILA families, we found that the families were born in overlapping waves of neurogenesis with dILA1 born at E10.5-E11.0, dILA2 born at E11.0, dILA3 and dILA4 born at E11.5, dILA5 born at E11.5-E12.0, and dILA6 born last at E12.0 (Fig. 2E,F and Fig. S10). Among dILB families, we found that the main birthdates for each group were as follows: dILB1 at E11.0, dILB2 at E11.0-11.5, dILB3 and dILB4 at E11.5, dILB5 at E11.5-12.0, and dILB6 at E12.0-12.5 (Fig. 2G,H, Figs. S11,12). These data showed an orderly progression of family birthdate and confirm the last-born status of the dILA6 and dILB6 families.

We concluded that families of dorsal horn neurons are born progressively across the period of late neurogenesis, and that temporal diversification is a major organizing principle of the dorsal horn.

## A chronotopic map of dILB families establishes the precise laminar structure of the dorsal horn

**Fig. 3:**
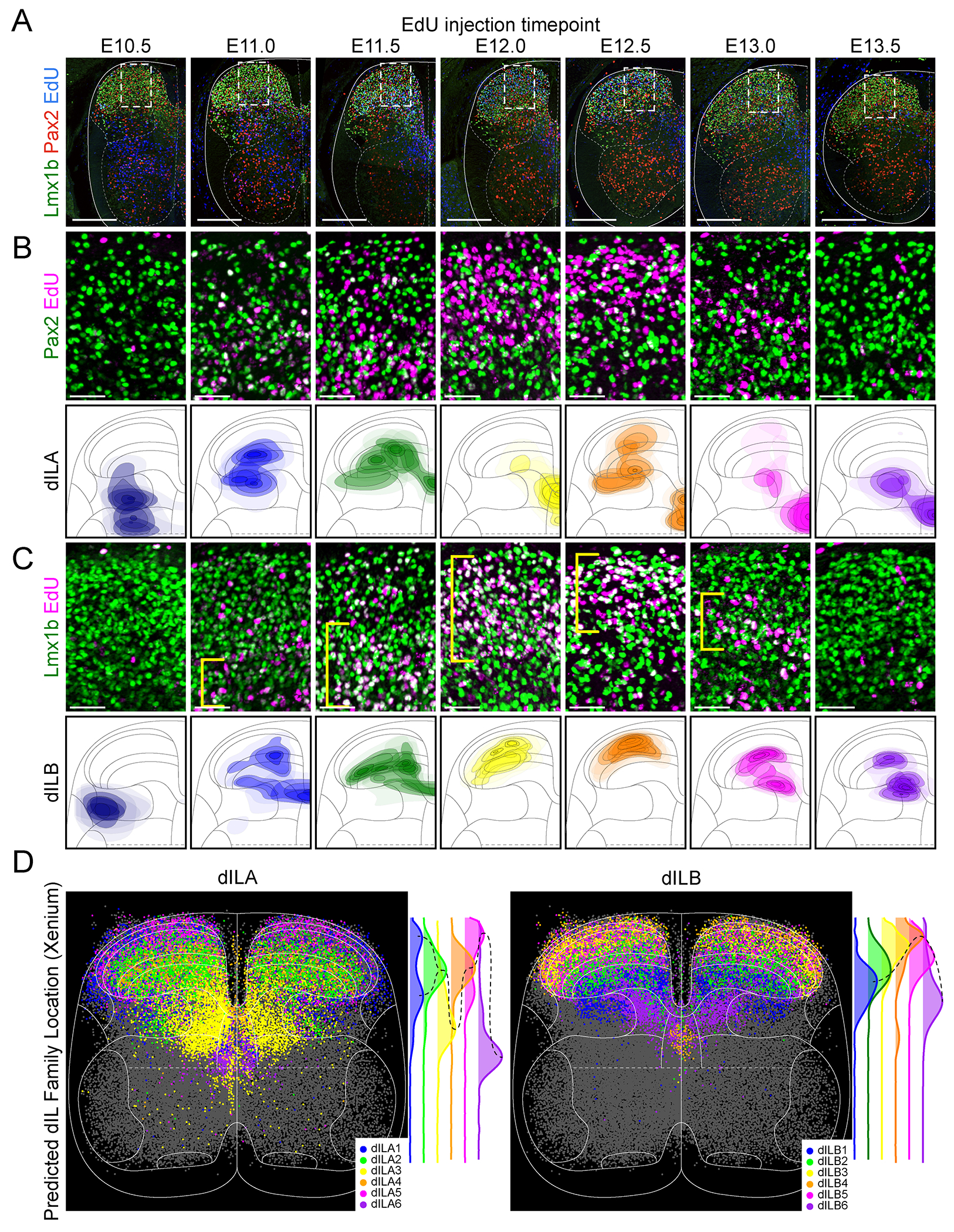
dILB neurons settle into a chronotopic map. (**A**) Images of embryonic spinal cord hemisections taken from separate E16.5 embryos that received EdU (blue) pulses at the indicated time points between E10.5 and E13.5, with immuno-labeling for the excitatory marker Lmx1b (green) and the inhibitory marker Pax2 (red). Solid and dashed white lines indicate approximate anatomical boundaries of the spinal cord. **(B,C)** Top rows depict magnified images from dashed boxes in (**A**), showing either Pax2+/EdU+ (dILA, **B**) or Lmx1b+/EdU+ (dILB, **C**) neurons at each of the time points indicated in (**A**), and with regions of frequent Lmx1b+/EdU+ co-labeling indicated with yellow brackets. Bottom rows indicate density plots of x,y coordinates of birth-dated dILA neurons (Pax2+/EdU+, **B**) or dILB neurons (Lmx1b+/EdU+, **C**) shown in the top rows of **B** and **C,** for EdU pulse experiments at each of the indicated time points in **A**. Separate perimeters were generated for each replicate per time point and for five proportions of labeled cells (50, 25, 15, 5, and 2.5%), colored in order of decreasing opacity. Replicates consist of all embryos of a single litter labeled with EdU at the indicated time point. (**D**) Scatter plots of neurons in data from Xenium spatial transcriptomics, annotated on the basis of predicted dIL family identity. Adjacent histograms demonstrate the frequency of neurons belonging to dILA or dILB families by depth. Probe sets used were the mouse brain panel v1.1 (248 genes) in addition to 100 custom genes from single-cell RNA-seq generated here (Table S12). Numbers: (**A-C**) n=79 embryos, 7-15 per time point. These data are derived from the same cohort of embryos depicted in Fig. 1A, see legend for details. (**D**) For spatial transcriptomics, cell coordinates were derived from six whole sections and seven hemisections (N=9.5 sections) from n=3 replicate wild-type E16.5 spinal cords. From these sections 54,166 cells were identified as neurons and plotted, of which 27,714 were annotated as dIL neurons. Scale bars: (**A**) 250 μm, (**B-C**) 50 μm.

A common theme of neural development, particularly in laminated structures, is chronotopy: an organizational principle in which progressively born neuronal subsets settle into ordered anatomical arrangements, providing the underlying structure for mature circuit function (38–41, 52). To explore whether successively born dILA or dILB neurons could account for the laminar structure of the dorsal horn, we examined the anatomical location of all dorsal inhibitory (Pax2+) and dorsal excitatory (Lmx1b+) neurons with respect to neuronal birthdate (EdU+, Fig. 3A, Fig. S13). We found that dILA neurons born at particular times had stereotypical spatial distributions, but often spanned many laminae and had no spatially congruent pattern across time points (Fig. 3B). In contrast, we observed that progressively born Lmx1b+ neurons settled into a coherent order: Putative dI5 neurons, born at E10.5 (23, 24, 53), were found in lamina I and lateral lamina V, followed by dILB neurons born at E11.0/E11.5 in laminae III/IV, those born at E12.0/E12.5 in laminae I/II, and those born at E13.0/E13.5 in part of laminae IIi/III and medial laminae V (Fig. 3C). Based on well-established links between laminar location and function, these data suggest a progressive development of excitatory neurons with projection neurons being born first (13, 34, 53), followed by neurons that process touch, then those that process pain, itch, and temperature, and finally those that contribute to proprioceptive processing and premotor control (2).

We next assessed the spatial distribution of each dILA/B family to determine whether the particular families followed the pattern of the overall Pax2+ and Lmx1b+ neurons. Xenium spatial transcriptomics was used to profile gene expression in tissue sections from E16.5 mouse spinal cords (n=3) and predicted cell identities (54) were mapped onto normalized cell coordinates from all replicates. As predicted by the EdU birthdating, we found that dILA families settled into stereotypical locations that lacked an overarching temporal-spatial order, whereas progressively born dILB families were found in progressively adjacent laminae (Fig. 3D, S14). The highest density of the first-born dILB1 family was found in laminae IV-V, dILB2 was in laminae III andIV, dILB3 was in laminae II and III, and dILB4 was in lamina IIo. The progression then reversed direction, with dILB5 settling broadly throughout laminae II and III and dILB6 in medial lamina V as well as a streak along dorsal lamina V. The arrangement of dILA and dILB families corresponded to the progression of Pax2 and Lmx1b-birthdated neurons across neurogenesis.

The chronotopic development of dILB families raised the possibility that excitatory neuron organization is the critical determinant of laminar structure in the dorsal horn. To test this hypothesis, we used mouse genetics to selectively remove each of the major classes of neurons from the dorsal horn: dorsal inhibitory neurons, dorsal excitatory neurons, and sensory neurons whose cell bodies are in the dorsal root ganglion but whose axons innervate the dorsal horn. We then compared the impact of each perturbation on the distribution of the remaining neural elements.

**Fig. 4:**
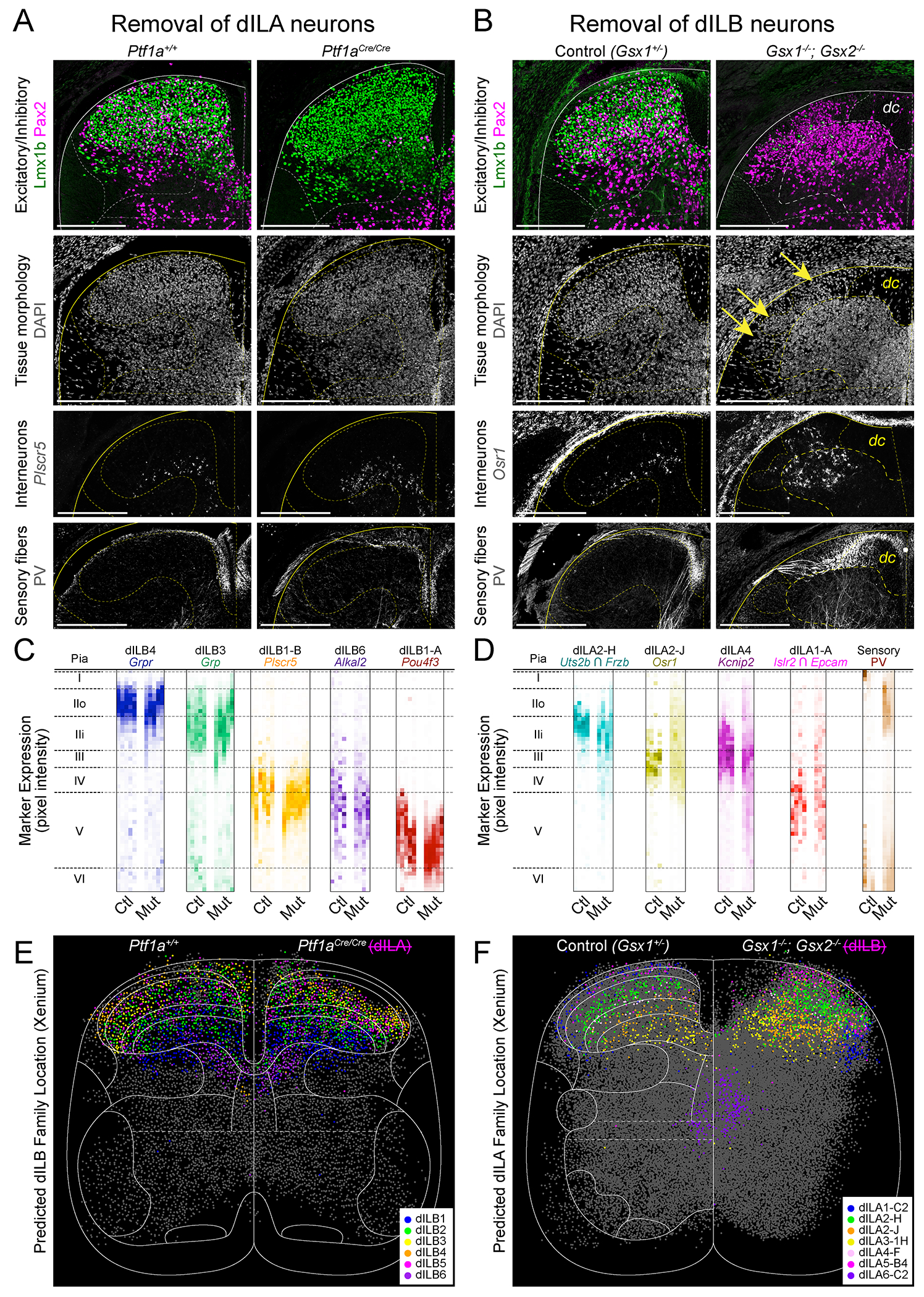
dILB neurons are required for laminar organization of the dorsal horn. **(A and B)** Images of E16.5 spinal cord dorsal horn hemi-sections from control and mutant embryos. (**A**) Sections from *Ptf1a*^+/+^ and *Ptf1a*^*Cre/Cre*^ embryos showing loss of Pax2-immunolabeled neurons (magenta), tissue morphology (DAPI, gray), an example marker of an excitatory interneuron (*Plscr5* RNA, gray), and proprioceptive sensory afferents [parvalbumin (PV) antibody, gray]. dc, dorsal column. (**B**) Sections from *Gsx1*^*+/−*^; *Gsx2*^*+/+*^ and *Gsx1*^−/−^; *Gsx2*^−/−^ embryos showing loss of Lmx1b-immunolabeled neurons (green), tissue morphology (DAPI, gray), an example marker of an inhibitory interneuron (*Osr1* RNA, gray), and proprioceptive sensory fibers (PV antibody, gray). **(C and D)** Spatial distribution of individual gene or protein markers or marker sets (as indicated at the top of each box), showing the normalized proportion of total signal per dorso-ventral bin (color intensity) where the pia is set as the dorsal boundary and approximate laminar positions are shown, for either (**C**) absence of dILA or (**D**) absence of dILB neurons. Biological replicates are grouped together as individual columns for either control (“Ctl”, *Ptf1a*^*+/+*^ or *Gsx1*^*+/−*^; *Gsx2*^*+/+*^) or mutant (“Mut”, *Ptf1a*^*Cre/Cre*^ or *Gsx1*^*−/−*^; *Gsx2*^*−/−*^) embryos. **(E and F)** Scatter plots of neurons in data from Xenium spatial transcriptomics, annotated according to predicted dIL family identity. (**E**) Brachial control *Ptf1a*^*+/+*^ embryo sections (n=3, left side) were compared with a single *Ptf1a*^*Cre/Cre*^ embryo section (n=1, right side). (**F**) Thoracic control *Gsx1*^*+/+*^; *Gsx2*^*+/+*^ embryo sections (n=3, left side) were compared with *Gsx1*^*−/−*^; *Gsx2*^*−/−*^ embryo sections (n=3, right side). Control neurons were downsampled to match the number of mutant cells displayed. Cells not belonging to the indicated dIL cell type are plotted in gray. Solid and dashed white and yellow lines indicate approximate anatomical boundaries of the spinal cord. Numbers: (A,D) Representative images were derived from n=4-6 *Ptf1a*^*+/+*^, n=3-7 *Ptf1a*^*Cre/Cre*^, n=3-4 *Gsx1*^*+/−*^; *Gsx2*^*+/+*^ and n=3-4 *Gsx1*^*−/−*^; *Gsx2*^*−/−*^ and histograms were derived from these same embryos (different numbers within each range were analyzed for each marker set depending on staining quality). (E,F) Spatial transcriptomics from E16.5 wild-type embryo brachial segments was previously described (Fig. S14). For E16.5 wild-type thoracic segments, cell coordinates were derived from five whole sections and one hemisection (N=5.5 sections), from n=3 replicate spinal cords. From these sections 27,758 cells were identified as neurons and plotted, of which 13,724 were annotated as dIL neurons. For E16.5 *Ptf1a*-null brachial segments, cell coordinates were derived from two whole sections (N=2 sections) from n=1 spinal cord. From these sections, 8,267 cells were identified as neurons, and of which 2,767 were dIL neurons. E16.5 brachial data were downsampled to 8,106 neurons of which 4,151 were dIL neurons to match. For E16.5 *Gsx1/2*-null thoracic segments, cell coordinates were derived from four whole sections and three hemisections (N=5.5 sections) from n=3 replicate spinal cords. From these sections, 24,737 cells were identified as neurons, of which 12,217 were dIL neurons. E16.5 thoracic data were downsampled to 24,583 neurons with, also, 12,217 dIL neurons, to match. Statistics: See Table S12. Scale bars: [(A) and (B)] 250 μm

To remove dorsal inhibitory neurons from the spinal cord, we used *Ptf1a^Cre/Cre^* homozygous null embryos, in which the dI4/dILA neurons are trans-fated to become dI5/dILB neurons (29, 55). As expected, Pax2^+^ cells were nearly absent from the dorsal horn, but the overall morphology of the tissue appeared normal (Fig. 4A). ISH and immunofluoresence for markers of dILB and sensory neuron types revealed that their typical spatial distributions were preserved (Fig. 4A,C and Fig. S15). Spatial transcriptomics performed on *Ptf1a^Cre/Cre^* tissue also revealed that predicted cell type locations for dILB families were normal (Fig. 4E and Fig. S16–17). Thus, inhibitory neurons were not required for the anatomical organization of the dorsal horn.

To remove dorsal excitatory neurons from the spinal cord, we used *Gsx1^−/−^; Gsx2^−/−^* double mutant embryos, in which dI5/dILB neurons are trans-fated to become dI4/dILA neurons (28, 56, 57). In Gsx double mutants, Lmx1b was nearly absent from the dorsal horn at E16.5, as expected, and the remaining tissue displayed abnormal morphology (Fig. 4B). There was a striking spatial disorganization of the remaining neural components of the dorsal horn. The restricted laminar distributions of dILA neuron types found in wild-type embryos were perturbed in double mutants, with some neuron types present in a mildly expanded range (Uts2b+/Frzb+ dILA2-H neurons) and others being scattered from the pia all the way to the base of the dorsal horn (Osr1+ dILA2-J neurons and Kcnip2+ dILA4 neurons) (Fig. 4B,D,F and Figs. S16,S18,S19). Despite this disorganization, dILA types destined for the same lamina were often found together in clumps, which may reflect cell-type intrinsic sorting mechanisms. Sensory fibers were similarly disorganized, with PV+ large proprioceptor fibers crossing through the superficial dorsal horn (Fig. 4B,D). We also observed neurons scattered among the dense longitudinal sensory fibers just below the pia, reflecting an abnormal boundary between the gray and white matter (Fig. 4B).

We also considered whether laminar organization could be imposed on dIL neurons by an extrinsic source. At E16.5, sensory fibers are the only other neural element in the dorsal horn, as descending axons from the brain have not yet penetrated the dorsal gray matter (58–63). To ablate sensory neurons, we used *Pax3^Cre^*;*Isl2^lsl-DTA^* embryos (64, 65), which express diphtheria-toxin in sensory neurons (and in some other neural crest-derivatives). As expected, this ablation led to loss of dorsal root ganglia and of sensory axons in the gray matter (Fig. S20). In addition, Lissauer’s tract and the dorsal columns were dramatically reduced in size and the dorsal horns often fused at the midline. Despite these changes, dILA and dILB neuron markers were present and displayed laminar patterns in the same dorso-ventral order as control spinal cords (Fig. S20) consistent with a prior report (66), indicating that sensory neuron innervation was not required for lamination.

Together, these data suggest a hierarchy of structure in the dorsal horn, in which excitatory neurons form laminar structures organized by birthdate and that inhibitory and sensory axons are dependent on this process to achieve their normal laminar distributions.

## Spatial-molecular diversification of dIL neuron families generates refined cell types

The temporal diversification process that generated the six dILA and six dILB families is unlikely explain the impressive molecular diversity of the ~150 refined dorsal spinal neuron types, so we next asked what other sources of variation may instruct dorsal neuron identity. To nominate candidate factors, we explored the most highly weighted genes for the top principal components explaining molecular variation within each dIL family (Table S14). This revealed that Zic transcription factors contributed highly to variation in nearly all families. In addition to *Zic1*, which we showed is expressed in post-mitotic dIL neurons (Fig. S3), there are four other mammalian members of the Zic zinc finger transcription factor family, *Zic2*, *Zic3*, *Zic4*, and *Zic5*, all of which have been shown to be present in the embryonic spinal dorsal horn, particularly within the progenitor domain (35, 36, 67–69). Zics can interact with the transcription factors that are downstream of Sonic hedgehog (Shh), transforming growth factor-β (TGF-β), and Wnt signaling – three major pathways that pattern the embryonic spinal cord (70–72). This led us to hypothesize that Zic factors may regulate heterogeneity among pdL progenitors and the identity of refined neuron types in the dorsal spinal cord.

**Fig. 5:**
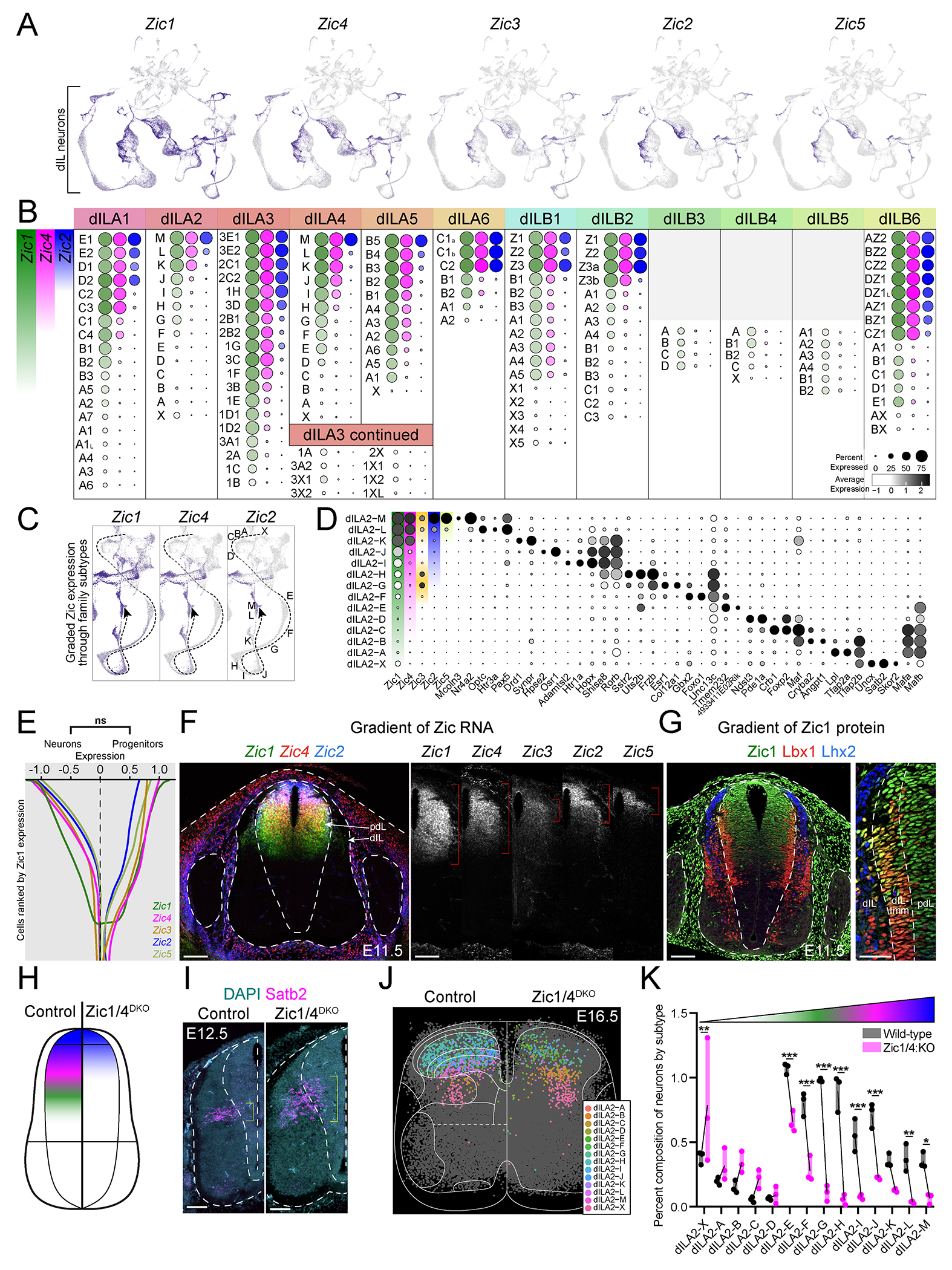
A progenitor gradient of Zic transcription factors patterns dIL neurons. (**A**) UMAP feature plots of *Zic1* to *Zic*5 expression in E14.5/E16.5 single cell data. (**B**) Dot plots of *Zic1* (green), *Zic4* (pink), and *Zic2* (blue) RNA expression in refined cell types within each dIL family, with dot size representing the percent of cells in each type expressing the gene and shade representing average expression amounts. (**C**) Gradients of Zic gene expression align with UMAP structure, within the dILA2 refined types M-X. (**D**) Dot plot of dILA2 neuron subtypes, showing unique markers for each type as well as groups of gene expression which co-vary along the Zic axis. (**E**) dIL neurons (left) and progenitors (right) on the y-axis, ranked by *Zic1* expression (x-axis), showing expression of each Zic gene. (**F**) Images of E11.5 spinal cord sections with ISH for Zic genes (left, merged; right single genes). (**G**) Image of E11.5 spinal cord section with immuno-labeling for Zic1 (green), a marker of newborn immature and migrating dIL neurons (Lbx1, red), and with Lhx2 (dI1, blue) shown for orientation. The ventricular zone is shown as the most medial area bounded by dashed white lines. Right panel shows higher magnification inset of newborn dIL neurons as they exit the ventricular zone. (**H**) Genetic strategy for perturbing the Zic gradient through double knockout (DKO) alleles of *Zic1* and *Zic4*. (**I**) E12.5 spinal cords from control and *Zic1/4*^DKO^ embryos, showing ISH for *Satb2* (magenta) and DAPI (cyan). (**J**) Scatter plots of normalized coordinates of neurons identified in Xenium spatial transcriptomics data, annotated to show dILA2 refined subtypes in control brachial sections (n=3, left) and in *Zic1/4*^DKO^ sections (n=3, right). Spatial transcriptomics from E16.5 wild-type embryo brachial segments was previously described (Fig. S14). For E16.5 *Zic1/4*^DKO^ brachial segments, cell coordinates were derived from three whole sections and one hemisection (N=3.5 sections) from n=3 replicate spinal cords. From these sections, 17,505 cells were identified as neurons, and of which 7,376 were dIL neurons. E16.5 brachial data were downsampled to 17,347 neurons of which 8,852 were dIL neurons to match. (**K**) Quantification of the data from (J) showing the relative composition of neuron subtypes as a percentage of all neurons, including replicates and lines connecting control and mutant means. Numbers: (A-D) N=94,048 neurons from single-cell RNA-seq data generated here (see Fig. 1B), (E) Single-cell RNA-seq data from dIL progenitors (N=1,963 cells) (31) and dIL neurons (N=61,444 cells, see Fig. 1B) , (F-G) n=3 E11.5 wild-type embryos, (I) n=3 E12.5 wild-type and n=3 E12.5 *Zic1/4*^DKO^ embryos, (J-K) n=3 wild-type and n=3 *Zic1/4*^*DKO*^ E16.5 mouse embryos. Statistics: **(E)** Dynamic time warping, comparing each combination of genes and cell types (45 comparisons). All comparisons between neurons and progenitors were not significantly different (p=1), suggesting that gene expression distributions are more similar than chance. **(K)** Two-way ANOVA (neuron subtype x genotype). For each neuron subtype in dILA2, Tukey’s multiple comparisons test was performed for each pair of genotypes, the result of which is indicated by asterisks above the plot. *<0.05, **p<0.01, ***p<0.001 Scale bars: 100 μm, except 50 μm in the magnified panel of (G) at the right.

We first assessed whether Zic gene expression in dILA and dILB post-mitotic neurons was related to the identity of refined neuron types. All five Zic genes were detected in UMAP feature plots among dILA and dILB neurons (Fig. 5A) and we observed corresponding expression of all five Zic factors in the dorsal spinal cord of E16.5 embryonic tissue sections (Fig. S21). Within the single cell data from all dILA families and most dILB families, Zic genes were expressed in a gradient, with refined cell types varying in both how many Zic genes were present and the amount of each individual Zic gene (Fig. 5B). *Zic1* showed the most widespread expression, followed by *Zic4*, *Zic2*, and then *Zic5*. *Zic3* was only found in restricted cell types. Focusing on one family, dILA2, the refined types were present along a trajectory in UMAP space that varied smoothly in Zic expression amounts from dILA2-M Zic^High^ neurons through dILA2-X Zic^Neg^ neurons (Fig. 5C). Ranking dILA2 refined types by their Zic expression revealed that, while each type expressed unique markers, there were also shared markers among types that were either Zic^High^ (eg. Pax5), Zic^Med^ (eg. Rorb), or Zic^Low/Neg^ (eg. Mafb) (Fig. 5D). Overall, this analysis revealed that a gradient of Zic gene expression sub-divided most dIL families into refined types of dorsal spinal post-mitotic neurons.

Cell fate restriction and diversification are typically established at the progenitor stage, but pdL progenitors have not been reported to display molecular variation. Since Zic expression has been reported previously in the dorsal spinal progenitor domain, we analyzed whether Zic expression varied in this context as well. Indeed, we found that a smooth gradient of Zic expression was present in single cell transcriptomic data from both E14.5 - E16.5 neurons and E11.5 - E12.5 progenitors, with *Zic1* and *Zic4* being the most broadly expressed and *Zic2* and *Zic5* being the most restricted (Fig. 5E).

In embryonic spinal tissue, Zic genes were present in a striking dorsal-ventral spatial gradient in the E11.5-E12.5 progenitor domain, with highest amounts in the dorsal-most part of progenitor domain and decreasing amounts towards the ventral spinal cord. *Zic1* extended the most ventrally, followed by *Zic4*, *Zic2*, and then *Zic5* which displayed the most dorsally restricted expression; *Zic3* had a distinct and more restricted pattern (Fig. 5F,G). The dorsal-ventral gradient of Zic1 protein expression was observed in both pdL progenitors and in post-mitotic Lbx1-expressing dILA/B neurons (Fig. 5G). Thus, Zic factors were expressed in a molecular-spatial gradient within pdL progenitors and their post-mitotic progeny.

Zic expression could simply correlate with dorsal neuron diversity or it could be an important driver of the cell type repertoire. To test whether Zic factors play an instructive role in dorsal neuron diversification, we examined *Zic3* mutant embryos that have lost a spatially restricted Zic (73, 74) and *Zic1/Zic4* double mutant embryos (DKO) that have lost the most widely expressed Zic genes (75). Unlike the other Zic genes, *Zic3* was detected in a faint bipolar gradient midway through the gradient of *Zic1* (Fig. 5E,F). Mutation of *Zic3* through the premature stop codon of the “katun” mutant resulted in a mild increase in Zic^Med^ refined types within several families (Fig. S22). This suggests that *Zic3* may act mainly as a transcriptional repressor to modify the repertoire of refined dIL neuron types.

We hypothesized that the loss of both *Zic1* and *Zic4* would have a more profound impact on dorsal neuron development, shifting the domain of Zic-expressing progenitors and neurons dorsally and expanding the domain of Zic^Neg^ progenitors and neurons. To test this prediction, we analyzed the expression of *Satb2* in spinal cords from *Zic1/4*^DKO^ embryos and control littermates. *Satb2* was a marker of a refined neuron type (dILA2-X) with expression that initiated in the transition zone near progenitors, thereby revealing the domain of origin of these newborn neurons around E12.5. As predicted, the domain of *Satb2*-expressing dILA2-X neurons was expanded and shifted dorsally (Fig. 5H,I). We next used spatial transcriptomics to assess the cell type composition of the dorsal horn in *Zic1/4*^DKO^ embryos and focused our analysis on dILA2 refined types. The Zic^Neg^ dILA2-X type was expanded and Zic^Low^ types showed modest increases as well. Conversely, nearly all of the Zic^Med^ and Zic^High^ dILA2 types were decreased upon loss of *Zic1/4* (Fig. 5J,K and Fig. S22–24). Similar results were observed in all other dIL families (Fig. S23, S24). Thus, the Zic molecular-spatial gradient, anchored by the broadly expressed *Zic1* and *Zic4* genes, was essential for the proper development of refined dIL neuron types.

## Discussion

We sought to uncover the general principles that govern the development of the spinal cord dorsal horn in mice, focusing on how its impressive cellular diversity and layered architecture emerge from a seemingly homogeneous pool of neural progenitors. We found that dorsal spinal progenitors give rise to multiple generations of neurons that varied transcriptionally based on the time and spatial position from which they originated. Specifically, each successive wave of newborn neurons gave rise to an excitatory or inhibitory coarse “family” of related neurons and each family was further divided into refined types based on a dorsal-ventral gradient of Zic transcription factors. Following neurogenesis, the young excitatory neurons settled into an orderly chronotopic map and we found that this organization was essential to the proper formation of the laminae of the dorsal horn. Altogether, we charted the detailed embryonic origins of mature dorsal neurons, discovered the developmental mechanisms that shape their molecular diversity, and revealed how their anatomical organization is established, thereby laying down the neural substrates for sensorimotor control of behavior.

How is the continuous flow of developmental time converted into the various dILA and dILB families? We propose that the conserved dorsal cell type families reflect the statistical modes of cell types generated over time, rather than discrete types separated by abrupt transitions. This model is supported by the overlapping emergence of dILA and dILB families in the precisely timed single cell data; the superimposed waves of peak birthdates that we observed for each family in neuronal birthdating analysis; and the small amount of “blurring” between temporally adjacent families. In this way, dorsal horn development may be similar to the formation of the mammalian retina, in which the generation of cell types is determined by the stochastic combination of progressive changes in a transcription factor code and the particular timing of progenitor cell cycle exit (76, 77). However, to determine the progenitor mechanisms of temporal cell type diversification in the spinal cord, *in vitro* modeling with synchronized progenitors, *in vivo* clonal analysis, and/or new sequencing-based methods for tracking cell fate relationships over time will be needed.

Not only does developmental time guide the emergence of dorsal horn families, but we found that it is etched onto the structure of the mature spinal cord. A general theme of laminated structures in the nervous system is the presence of an anatomically organized microcircuit with precise afferent and efferent connections in each layer (78–81). This is certainly the case for the *adult* spinal dorsal horn, particularly for sensory and descending brain axons that target specific lamina to cooperatively mediate sensorimotor control. Our data suggest that the chronotopic development of the dILB neurons establishes the spatial landscape to receive these inputs at their proper locations. Progressively born excitatory dILB neurons settled into neighboring layers and their loss profoundly disrupted the spatial organization of remaining neural elements including inhibitory neurons and sensory afferents. In contrast, loss of inhibitory dILA neurons or sensory neurons preserved normal laminar organization, whereas descending axons from the brain only target spinal neurons after the lamina have already been established. The factors that mediate dILB-guided anatomical sorting are not known, but they likely comprise a combinatorial code of adhesion-based matching (such as cadherins, neurexins, and Dscam), axon guidance related factors (such as Eph/ephrins and Slit/Robos), and chemotactic cues (such as growth factors and Trk receptors). Regardless of the particular molecular mechanisms, an autonomous role for dorsal spinal neurons in self-organizing into their characteristic layers is further supported by the intrinsic ability of cultured dorsal interneurons to form laminae upon transplantation into spinal tissue, with sensory and corticospinal axons invading these miniature dorsal structures in a spatially appropriate manner (82). Together, these findings suggest that excitatory dorsal neurons form a laminar map which is imparted onto inhibitory dorsal neurons, sensory neurons, and likely also descending pathways from the brain.

Until now, late dorsal progenitors were thought to be a homogeneous group, raising questions about how the impressive diversity of neuron types is generated. We found a dorsal-to-ventral gradient of Zic transcription factors, consistent with prior work showing Zic factors in the neural plate boundary and dorsal neural tube where their expression is determined by a combination of Wnt signaling, BMP (bone morphogenetic protein) inhibitors, and the absence of Shh or notochord influence (35, 36, 83–85). We found that the Zic gradient patterned pdL progenitors, created an array of variants within dIL families, and was required to generate the normal complement of dorsal neurons. Zic transcription factors likely act through multiple mechanisms to pattern dorsal neuron cell fate. First, they can directly regulate expression of the transcription factors *Atoh1*, *Ascl1*, and *Pax3* that establish dorsal lineages during early spinal development (35, 70, 86, 87). Second, Zic transcription factors can physically interact with and modulate the function of key transcription factors in the dorsal horn including Pax3, Smad2/3, and Tcf/Lef proteins (72, 88, 89). Third, Zics can modulate the function of Gli transcription factors that mediate Hedgehog signaling in the ventral spinal cord (70), raising the possibility that Gli and Zic proteins may work together to establish countervailing ventral and dorsal molecular gradients. And fourth, expression of Zic factors can be sustained in dorsal neurons through adulthood which may indicate ongoing roles in neural identity maintenance (90). Future work should probe the molecular mechanisms that induce the Zic gradient and transform it into gene expression signatures that characterize dorsal horn neurons. In particular, *in vitro* pluripotent cell paradigms have the potential to inform these questions through the ability to control the identity, timing, and concentration of candidate inducing proteins, the use of culture substrate geometry to probe the role of spatial constraints, and high-throughput perturbation screens of candidate cell autonomous factors and Zics themselves.

Comprehensive alignment of embryonic and postnatal cell types has the potential to unify key features of cell types. As examples, we highlight three refined populations that were born around E11.5. (1) dILA2-H cells were born at E11.0-E11.5, have intermediate amounts of Zic RNA expression, and likely give rise to dorsal inhibitory neurons in lamina II with a predominantly tonic firing electrophysiological profile, islet cell morphology, and expression of *Rorb*, *Calb2* (Calretinin), and *Tac1* (91, 92). They receive primary sensory input from non-peptidergic nociceptors and provide pre-synaptic inhibition to these afferents as well as post-synaptic inhibition to interneuronal targets; they are thought to contribute to acute pain and itch processing (8, 91). These cells have been called Inhib-3 in mouse, INH-NXPH1 in monkey, and Inh-Dorsal-3 in human spinal cords (3–5). (2) dILB2-A4 cells were born at E11.5 and likely give rise to excitatory neurons in lamina III that express *Maf*, *Cck*, *Adarb2*, and high amounts of *Rora* (13, 16, 93–95). They receive cutaneous low threshold mechanoreceptor sensory and corticospinal inputs (8, 93) and process light touch sensory information for behaviors such as corrective foot placement reflexes (13, 16). They also contribute to dynamic allodynia in the context of inflammatory or neuropathic pain (94). They have been called Excit-5 in mouse, EXC-MAF in monkey, and Ex-Dorsal-4 in human spinal cords (3–5). (3) dILA3-1X and -2X cells were born at E11.5, are Zic-negative, and likely give rise to dorsal inhibitory neurons in lamina V that express *Satb2*. They receive primary proprioceptive inputs and target spinal motoneurons directly as well as numerous interneuronal cell types in the ventral horn (17, 18). Developmental perturbation of these cells results in limb hyperflexion and abnormal reflex responses, reflecting a putative role in sensorimotor integration (18). These cells have been called Inhib-17 in mouse and Inh-M-1 in human spinal cords (4, 5).

Overall, our work supports a model in which four major principles govern dorsal horn ontogeny. First, six temporal cohorts of dIL neurons are born successively and give rise to the families of dorsal neuronal types. Second, each temporal cohort is patterned along a dorsal-ventral axis by Zic transcription factors, thereby fractionating each family into a range of variant types. Third, individual pdL progenitors can give rise to inhibitory dILA or excitatory dILB neurons as directed by Notch signaling, a process we did not study here but which has been shown previously (28, 30). Fourth, the excitatory neurons establish the anatomical structure of the dorsal horn by settling into an ordered distribution based on their birthdate.

As this picture comes into focus, the similarity to drosophila optic lobe development is notable: There is a similar number of discrete transcriptional cell types, similar temporal diversification which is transformed into a spatial arrangement for precise circuits, similar dorso-ventral spatial patterning that is most likely coordinated by the same signaling pathways (Shh/Hh, BMP/Dpp, and Wnt/Wg), similar binary cell fate decisions controlled by the Notch pathway, and even a possible similar role for Zic/Opa factors in regulating neuronal diversity (96–98). These shared features in evolutionarily distant tissues and organisms may be coincidence, or they may reflect convergent forces that produce sensory systems specialized for multi-modal processing. In particular, the high degree of neuronal diversity (generated by the three-fold combination of temporal, spatial, and Notch mechanisms) and the layered anatomical microcircuit may facilitate the parsing, sharpening, blending, integration, and feature extraction from different neural information streams. The next frontier belongs to contextualizing this cellular diversity and structure in terms of behavior – that is, to reconcile these neuronal elements with their contributions to sensation, autonomic control, and movement.

## Materials and Methods

### Animal husbandry and ethical approvals:

The following mouse strains were maintained at the National Institutes of Health in Bethesda, MD, and all experiments received assent from the National Institute of Neurological Disorders and Stroke Animal Care and Use Committee (ACUC protocol number 1384). All wild-type mice used in this study were a mix of BALB/cJ and C57BL/6J strains. *Pax3^Cre^*, *Isl2^DTA^*, and *Zic1/Zic4^+/−^* mice were obtained from The Jackson Laboratory (Bar Harbor, ME, USA).

The *Gsx1^+/−^* and *Gsx2^+/−^* mouse strains were maintained at Cincinnati Children’s Hospital Medical Center, and experiments involving these strains were reviewed and approved by the Cincinnati Children’s Hospital Medical Center Institutional Animal Care and Use Committee.

The *Zic3^Ka^* mouse strain was maintained at Australian National University. Mice were maintained according to Australian Standards for Animal Care under protocol A2021/39 approved by The Australian National University Animal Ethics and Experimentation Committee. The katun (*Ka*) allele (MGI: 3043027) of *Zic3* (*Zic3^Ka^*) (73) was maintained by continuous backcross to C57BL/6J inbred mice; animals from backcross 10 and beyond were used for analysis.

The *Ptf1a^Cre^* mouse line was maintained at the University of Texas Southwestern. All mouse protocols were approved by the Institutional Animal Care and Use Committee at the University of Texas Southwestern Medical Center. All mouse strains were maintained on a mixed background of ICR and C57BL/6.

Only *Zic3^Ka^* embryos were sex-typed (owing to *Zic3* being on the X-chromosome), where even numbers of male and female wild-type and null embryos were examined. Embryos of all other experiments were not sex-typed and experiments were assumed to contain an even male:female ratio. Mice housed at all facilities were given ad-libitum access to food and water, maintained on a 12-hour light:dark cycle (6:00-18:00 light period; following daylight savings time).

### Embryonic spinal cord tissue:

Appropriate pairs of male and female mice were introduced to the same home cage at ~ 18:00 and checked daily thereafter each morning at approximately 10:00 for vaginal plugs. Noon (12:00) on the day a plug was detected was considered E0.5, and embryos were dissected at approximately noon on the embryonic day indicated by the experiments described here, in the Materials and methods and in Results section. Particular care was taken to ensure that EdU birthdating was as accurate as possible; for these experiments, males and females were paired only one night per week and separated in the morning regardless of whether a plug was detected. Only females that plugged received a subsequent intraperitoneal injection of EdU (more details below), ensuring that time of conception was constrained to approximately the dark period (i.e. with an uncertainty of plus or minus 8 hours).

On the indicated embryonic day, female mice were administered CO2 euthanasia, and embryos were promptly dissected and washed in ice-cold 1X phosphate-buffered saline [PBS; except in cold Hanks buffered salt solution (HBSS) for preparation for single-cell suspensions in the 10X Chromium assay]. The spinal column was severed at the spinal cord-hindbrain junction, and the column was further dissected from the torso. For experiments prepared at the National Institutes of Health, the vertebral body was further dissected to expose the ventral surface of the spinal cord to fixative; for experiments performed elsewhere, dissection only involved isolation of the column with contained spinal cord. Spinal columns were immersion-fixed in ice-cold 4% paraformaldehyde (PFA) for either 4-6 hours for immunohistochemical applications, or overnight (20-24 hours) for ISH applications (both 10X Xenium and RNA Scope). After fixation, tissue samples were briefly rinsed in 1X PBS (<10 minutes) and then equilibrated to a solution of 30% sucrose plus 1X PBS overnight or until tissue sunk to the bottom of the vessel containing the tissue. In all cases tissues were agitated on an orbital or nutating shaker (depending on the vessel type) during the fixation and cryoprotection intervals to facilitate equilibration of tissues to the PFA or sucrose solutions.

### Genotyping:

Genotyping was done as described by original publications for each allele. Expected band sizes are: Ptf1a^Cre^ [Wild-type: 123 base pairs (bp), Cre: 300 bp], Zic1/4^DKO^ (Wild-type: 465 bp, Mutant: 400 bp), Gsx1^+/−^ (Wild-type: 219 bp, Mutant: 180 bp), Gsx2^Ra^ (otherwise referred to as Gsx2^+/−^, Wild-type: 298 bp, Mutant: 130 bp). Pax3^Cre^ and Isl2^DTA^ embryos were genotyped by Transnetyx (Cordova, TN, USA) using custom primer sets for the alleles (see Jackson Laboratory pages for original primer sets). For purposes of colony maintenance, Pax3^Cre^ mice were genotyped by the presence of a white splotch on their belly fur at the time of weaning (typical of mice bearing one null Pax3 allele). *Zic3^Ka^* embryos were genotyped as sex-typed as follows: Genomic DNA was prepared for genotyping as previously described (74); 50 ng of genomic DNA was added to each genotyping reaction. The *Zic3^Ka^* strain was genotyped through allelic discrimination using the oligonucleotides Ark241_Katun_F and Ark242_Katun_R and probes Wildtype: 5’ 6FAM and G>T mutation: 5’ VIC, with TaqMan Universal PCR Master Mix (Life Technologies; Cat. No. 4304437). The products were amplified and analysed using a StepOnePlus^™^ Real-Time PCR System (Applied Biosystems) and StepOne software (version 2.2.2; Applied Biosystems). The embryos were sex-typed using MyTaq HS Red (Bioline: BIO-25048) and touchdown polymerase chain reaction (PCR) with an initial annealing temperature of 60°C, as previously described (74). The sex was determined using the oligonucleotides Ark1004_Smcx_y_F and Ark1005_Smcx_y_R. Amplicons were separated by agarose gel electrophoresis (2% agarose in 1X tris-borate-EDTA buffer).

### Birthdating of spinal cord tissue:

Pregnant female mice (detailed above) were injected with an intra-peritoneal solution containing 50 mg/kg of EdU (Thermo Fisher Scientific) at a concentration of 2 mg/ml in sterile 1x PBS. Injections were performed on separate pregnant females at either 12:00 (for the midpoint of an embryonic day - i.e. E12.5) or 0:00 (for the beginning of an embryonic day - i.e. E12.0). Only one injection at only one particular time point was performed for each individual female. Two separate cohorts of embryos were produced for the present timecourse – an initial cohort which were briefly fixed (4-6 h) suitable for immunohistochemistry (IHC; examined in Fig. 1 and 3), and a second cohort where equal numbers of embryos from each litter were fixed for either 4-6 h or for 20-24 h (more suitable for ISH) and which were examined in Fig. 2. A cohort of embryos with IHC and ISH-suitable fix conditions were necessary to make within-litter comparisons of EdU co-labeling between all dIL families, some of which are best labeled with either IHC or ISH. Histochemistry to detect the presence of EdU in spinal cord nuclei is detailed below.

### Cryosectioning:

All tissues were frozen while immersed in optimal cutting temperature (OCT) compound in a cubic mold. Samples from littermate embryos were always embedded and frozen in the same block of OCT. Molds were immersed in 100% ethanol chilled with dry ice up to the top of the mould, although direct contact of OCT compound and ethanol was avoided. Blocks were maintained at −80 °C after freezing until cryosectioning, at which points blocks were equilibrated to the cryostat temperature (generally −18 °C to −24 °C) and cut into 25 μm sections (except for 10X Xenium, which were cut at 10 μm). Sections were mounted on microscope slides which were then stored at −80 °C before to histochemistry.

### Immunohistochemistry (IHC):

All IHC was performed using published primary antibodies (detailed in Table S15). A wide variety of secondary antibodies were used here and were Donkey IgG Alexa-fluor conjugates purchased from either Invitrogen or (for Donkey anti-Guinea pig secondary antibodies) Jackson Immunolabs. Working dilutions of antibodies from their manufactured concentrations are detailed in Table S15. Briefly, slides were removed from −80 °C and dried at 37 °C for at least 20 minutes. Sections were then surrounded by a hydrophobic barrier (Vector Laboratories) and dried at room temperature another 5 minutes. Sections were then washed three times successively for 10 minutes each using cold 1x PBS at 4 °C. Working dilutions of primary antibodies were prepared in 1x PBS with 0.1% Triton X-100, applied inside the hydrophobic barrier, and incubated overnight at 4 °C. Subsequently slides were again washed three times successively for 10 minutes each using cold 1x PBS at 4 °C. Working dilutions of appropriate Alexa-Fluor-conjugated secondary antibodies together with 4’6-diamidino-2-phenylindole (DAPI; for nuclear staining) were prepared in 1x PBS with 0.1% Triton X-100, applied inside the hydrophobic barrier, and incubated for 1 hour at 4 °C. Finally, slides were once again washed three times successively for 10 minutes each using cold 1x PBS at 4 °C. After the final 1x PBS wash was removed from the slide, 2-4 drops of Prolong-Gold (Thermo Fisher Scientific) were applied to the slide and the coverslip was gently laid to spread the medium and avoid trapping bubbles. Covered slides were allowed to dry at least 1 hour before imaging and stored at 4 °C before and after imaging.

### In-situ hybridization:

All ISH was performed according to the published protocols for ACD Bio RNA Scope Multiplex Fluoresence (version 2) assay. All reagents and probes were manufactured by ACD Bio and are listed in Table S15. Target retrieval was omitted from the tissue pretreatment protocol for all tissues examined here, owing to the step producing excessive damage to embryonic sections. Probe fluorescence signal was not evidently changed in embryonic spinal cord sections receiving versus not receiving target retrieval. Briefly, sections were dried for 2 hours at room temperature before the modified tissue pretreatment protocol. Hybridization, development of fluorescence signal, nuclear staining with DAPI, and cover-slip application was done exactly as described by protocol. Covered slides were allowed to dry for at least 1 hour before imaging and stored at 4 °C before and after imaging. Fluorescence was developed using the newer TSA-Vivid dyes (Vivid 520, Vivid 570 and Vivid 650).

### Pixel-frequency depth histograms:

For selected IHC and ISH signals, histograms of pixel-frequency were gathered from E16.5 wild-type, *Ptf1a*-null, *Gsx1/2*-null and sensory neuron-ablated spinal cords. This was done using the Plot Profile function of ImageJ/Fiji on 8-bit gray thresholded images. To measure pixels where two markers colocalize images of both markers were separately thresholded and pixels were multiplied using ImageJ/Fiji’s image calculator, with the histogram being applied to the product image. One to three sections per biological replicate were analyzed and, whenever possible, each hemisection was measured separately. The histogram was collected vertically, with one end of the range at the pial surface of the superficial dorsal horn midway through its mediolateral extent, and the other end of the range at the dorsal-ventral border of the ventricular midline (where both sides become adhered). The histogram width (i.e. how much sampling was done) varied, and generally encompassed one third of the total dorsal horn width except smaller when avoiding sampling autofluorescent vasculature. Histogram bins were compressed to a common number (80) to allow comparisons between genotypes, and bin frequencies were normalized to the total counts for that particular bin per replicate. Lamina VI was mostly excluded from this analysis owing to the lack of dILB neurons there; accordingly, the dorsal-most 56 bins were plotted in figures, with color saturation corresponding to bin weights. Histograms were divided into quintiles dorsoventrally for statistics. Three-way analysis of variance (ANOVA) was run on each of three experiments described above (genotype x quintile x cell-type) and post-hoc tests were run to determine differences in cell-type composition per dorsoventral quintile (see Table S12).

### EdU histochemistry reaction:

In the event of EdU histochemistry, slides being reacted for IHC or ISH were not covered at the end of the protocol; instead, they were washed in 1x PBS with 0.1% Triton X-100 for 30 minutes at 4 °C. After this, they were incubated at room temperature in a solution of EdU reaction buffer, buffer additive, copper (II) sulfate, and Alexa-Fluor-conjugated azide (Alexa-Fluor-488 used in conjunction with Vivid-570 and Vivid-650 dyes on RNA Scope-reacted slides, or Alexa-Fluor-647 with IHC-reacted slides having omitting Alexa-Fluor-647-conjugated secondary antibodies) at the ratios indicated for the Click-iT EdU Cell Proliferation Kit (Thermo Fisher Scientific). They were subsequently washed three times successively for 10 minutes each using cold 1x PBS at 4 °C, cover-slipped and stored per IHC protocols above.

### Microscopy:

All micrographs presented here were obtained either from inverted confocal microscopes (Zeiss LSM-800), or from a Zeiss AxioImager Z2 equipped with Apotome 3 structured illumination and DAPI, GFP, DsRed, Cy5, and Cy7 filter cubes using Zeiss ZEN software. In either case, micrographs were composed of a tile-scan of spinal cord sections using a 20x objective as well as a z-stack of six to nine intervals centered approximately mid-way through the tissue section’s thickness. For experiments using Apotome, five images were taken per field of view, each using different positions of the structured illumination grid, and were deconvoluted using ZEN’s Apotome RAW Convert function. Image arrays from both Apotome and confocal experiments were stitched using Zeiss ZEN’s stitching function; all channels at all z-positions were stitched with reference to nuclear DAPI signal from images in the mid-point of the stack. Images were stored in Zeiss’ .czi format, and were subsequently loaded into Fiji (Image J, v2.3.0) with the current Bio-Formats plugin.

### EdU counts:

All birthdating in this study was done on cervical spinal cord sections, between C3-C8. Images with EdU and signal from two other markers (either from IHC or ISH) were counted in ImageJ using the Cell Counter plugin. Cells were considered positive for EdU if the nucleus was mostly filled with signal and excluded if the nucleus was a discontinuous set of puncta. Overlap with either transcription factor IHC signal or with RNA Scope was considered as a positive colocalized cell and was counted separately to either cells only labeled with EdU or cells only labeled with cell-type markers respectively. Anchor points were labeled on each section to allow coordinate normalization in data processing. Briefly, a set of 10 points were added to each section: two on either dorsal horn’s most dorsal extent; two on either dorsal horn’s lateral-most extent, one at the base of the dorsal columns, two on either ventral horn’s lateral-most extent, two on either ventral horn’s most ventral extent, and one on a point along the midline where ependymal/progenitor cells diverge dorsally but are adhered ventrally (this point is considered to be the boundary between dorsal and ventral horns). Coordinates are then exported using the Measure tool. When examining the birthdate of dIL families, RBR blinded and analyzed unblinded data while AJL counted blinded images; these roles remained fixed throughout the experiment.

### EdU counts: Cumulative birthdating calculations and statistics:

At least one embryo was assessed per litter; for each embryo, the fraction of co-labeled neurons as a fraction of all neurons of any dIL family at any particular time point of EdU pulse were represented as a fraction. The mean of these fractions across all embryos of a litter was counted as a single data point, as we judged that variance across litters was higher than within litters, hence the insufficiency of only one litter for this analysis (regardless of whether that litter had embryos in triplicate).

To produce cumulative counts for each time point, the mean fraction of dIL family neurons co-labeled with EdU for each time point was produced. This value was then normalized to the fraction of mean co-labeling across all time points, so that it reflected the percent of dIL family neurons born at a certain time point within our measurements. From here, mean co-labeling for all prior time points were added to the replicate data of each time point such that within time point variation would be preserved but that mean cumulative co-labeling at the final time point would be 100%. Data were plotted as mean ± standard deviation and sigmoidal fit curves were calculated and plotted in Graphpad Prism using “XY analyses” -> “Interpolate a standard curve” -> “Sigmoidal, 4PL, X is concentration”. Sigmoidal fit could not be done for two time points (dILA2 and dILB1) where substantial neurogenesis between E10.5 and E11.0 produced a negligible slow-growth phase to the curve; these curves were instead calculated according to the “Asymmetric Sigmoidal, 5PL, X is concentration” fit. EC50/IC50 values reported by the standard curve interpolation were plotted as vertical lines on the time (x-) axis of standard curve plots in colors corresponding to each of six dILA or dILB families respectively.

To assess statistically significant differences across the birthdating curves, we fit a logistic (sigmoid) curve to each time series replicate using the ‘SSlogis’ function in R, using the time point as the x variable and the % of cells as the y variable. We then assessed whether the distribution of inflection point (on the x axis, or time points) values differed across the cell types, using a standard t-test with FDR correction. We also assessed whether the distribution of the scaling factor (measuring the steepness of the curve) values also differed across the cell types, using a standard t-test with FDR correction. Results are noted in Table S12. Confidence is generally higher for families on either end of the birth-dating range, but falls near the middle, perhaps owing to rapid changes in output near the peak of neurogenesis.

### Cell coordinate rotation and normalization:

For EdU cell count coordinates (and later also for spatial transcriptomics cell coordinates), cell coordinates were normalized so that statistics and graphics could be produced systematically on cell coordinates across sections and experimental groups. To do this, anchor points (described in the EdU counts section above) were used to transform all coordinates identified from a section. Two midline coordinate pairs (from the dorsal columns base and at the dorsalhorn-ventral-horn junction) were used to rotate all coordinates such that both coordinate pairs had identical x-coordinates. Each hemisection was then stretched to fit an ideal diagram of a spinal cord; y-coordinates from the coordinate pairs at the dorsal surface of the dorsal horn and ventral surface of the ventral horn were normalized to either +2000 or −2000 arbitrary units of distance. Likewise, the x-coordinates of coordinate pairs at the lateral-most extent of each dorsal horn were normalized to either −2000 or +2000. X and Y coordinates of all intervening spinal cord neuron data were linearly transformed on the basis of their distance from these anchor points. Additionally, neurons were linearly transformed based on their proximity to the y-coordinates of the lateral dorsal horn coordinate pairs or to the y-coordinate of the anchor at the base of the dorsal columns. The normalized value for these were also arbitrary, and changed on the basis of segmental group to best fit an ideal spinal cord diagram; because of this, statistical comparisons between data from different spinal cord segments were not done – only within segment and between EdU pulse time (or genotype). Cell coordinates from all conditions were collated in a single database and analyzed further.

### Contour plots:

Owing to variability in time of conception, a certain amount of overlap in terms of the patterns of EdU-dIL family co-labeling was expected and observed for adjacent families, and between group differences are difficult to judge on a scatter plot. We therefore prepared and plotted two-dimensional kernel density estimates for EdU birthdating cell coordinate data. This was done using the kde2d function from the R MASS library. Kernel density estimates were produced at a resolution of 100 for cell coordinates from all litter replicates and all EdU time points, which were then collated in a database. Contours were plotted in ggplot2 using geom_contour_filled and drawn to fit the densest 50, 25, 15, 5 and 2.5% of points. Broader contour levels were drawn with greater transparency to represent lower cell density in these levels.

### Cell preparation for single-cell RNA-seq:

Female wild-type mice with a pregnancy dated to embryonic day E14.5 or E16.5 were used. Each experimental replicate consisted of a pooled litter of 3 to 10 spinal cords taken from embryos of one single pregnant female.

Embryos were rapidly dissected and immersed in cold 1X PBS. Spinal cords were sequentially dissected as described above and placed in cold dissection medium [50 ml HBSS-calcium/magnesium-free with phenol red (Gibco) + 750 μl HEPES (1M, Gibco)]. When possible, extra care was taken to remove meninges and dorsal root ganglia from the isolated cord. From here, appropriate spinal cord segments were dissected from the cord, and then cut into fine pieces with dissecting spring scissors. Either cervical, thoracic or lumbosacral cords were isolated. In either case, care was taken not to include transitional zones of the cord (i.e. where cervical tapers into thoracic or where thoracic begins to enlarge to lumbar); because of this, these regions are likely underrepresented in our data.

Pooled spinal cord tissues were then added to a 15 ml conical tube containing dissociation medium (100 units Papain (Worthington), 4.75 ml HBSS+CM (calcium and magnesium; Gibco), and 250 μl of DNAse (Worthington, 1000 units resuspended in 500 μl of HBSS+CM)). The tube was gently inverted several times every 5 minutes while leaving the tube to incubate at 37 °C between inversions. After 20 minutes, the suspension was triturated gently with a series of three fire-polished glass pipettes (Fisher Scientific) with progressively finer bores. Suspensions were then centrifuged for 8 minutes at 850 rpm; after this, the supernatant was removed and the cells were gently resuspended with a solution of 4.5 ml HBSS+CM, 600 μl ovomucoid protease inhibitor solution (Worthington, resuspended in 30 ml HBSS+CM), and 250 μl DNAse (as above). Debris was allowed to settle for 10 minutes, at which point the supernatant with suspended cells was added to a second 15 ml conical tube containing neutralizing solution [10 ml HBSS+CM + 100 mg Trypsin inhibitor (Sigma) + 100 mg bovine serum albumin (BSA; Sigma)]. This suspension was centrifuged once more for 8 minutes at 850 rpm, and the supernatant was removed. The remaining cells were resuspended in ~ 1 ml of 1X PBS containing 5 μl of molecular-grade BSA (New England Biolabs) and 2 μl of RNAse-inhibitor (Biosearch Technologies). Cell concentration in this suspension was assessed using a standard hemacytometer (a 50:50 sample of cell suspension + Trypan blue). The cell suspension was diluted with the same resuspension medium until the final concentration was appropriate for loading into the 10X Genomics Chromium chip.

### Single-cell RNA-seq - data collection:

Cell suspensions were introduced to 10X Genomics Chromium chips at concentrations appropriate for retrieval of data from an estimated 7,000 cells. Two Chromium wells were loaded per pooled biological replicate, with an anticipated 14,000 cells retrieved per replicate. cDNA and libraries were constructed on the basis of the 10X Genomics Chromium 3’ gene-expression chemistry (v3.1). Library fragment size and variation were inspected on an Agilent 2100 bioanalyzer, then pooled for sequencing. Sequencing was done using the Novaseq 6000 platform, with the aim of sequencing 40,000 reads per cell.

Published datasets were retrieved from ArrayExpress (accession number E-MTAB-7320) or GEO (accession number GSE228590), per instructions from original publications (31, 49).

### Single-cell RNA-seq - data processing:

Raw sequencing data were processed first with Cellranger mkfastq, and those outputs were then run in Cellranger count to produce count matrices. Reads were mapped onto the *mus musculus* GRCm38-mm10 genome reference. Post-hoc quality control per sample (including effective number of cells retrieved, number of reads and genes detected per cell, etc) are detailed in Table S1. Sample 16B (E16.5 cervical spinal cord replicate 1 reaction 2) was excluded from further analyses at this stage because of an observably poor emulsion post Chromium run, poor read quality and almost negligible cell retrieval. Further quality control, clustering and annotation were done using Seurat v4 (although Seurat v5 was released in the interim) in R (v4.4.1). To begin in Seurat, data from each sample were imported into Seurat and minimum thresholds on count and gene numbers per cell, as well as maximum thresholds on mitochondrial gene percentages per cell were imposed (Table S1). From this, the data were log-normalized, variable features were found, and CCA integration (Seurat v4) was run using a list of biological replicates as inputs. At this stage, 195,146 cells were retained.

Principal component analysis (PCA) was run on the integrated dataset of all biological replicates using 90 PCs, while also regressing gene counts per cell. After clustering all cells at resolution 2, clusters were annotated as major cell-types of the spinal column based on top marker genes per cluster (Table S2). Spinal cord neurons were extracted from this broader dataset (Fig. S1; annotation classes “Spinal neurons” and “Immature spinal neurons”) of sensory neurons and non-neuronal cells using the inclusion criteria of *Nhlh1*, *Neurod4*, *Tubb3*, *Rbfox3*, *Slc17a6* (vGluT2) and *Slc32a1* (vGAT) identifying neurons and immature neurons, as well as the exclusion criteria of *Avil* and *Tlx2* identifying sensory neurons, and the spinal cord neurons subset was further processed.

Principal component analysis of spinal cord neurons was once again done using 90 PCs, and the dataset was subsequently re-clustered, excluding clusters whose principal marker gene sets were non-neuronal, whose principal marker genes were a combination of neuronal and non-neuronal in addition to substantially higher gene counts (assumed doublets), and/or whose features were of a variety of cell-types in addition to substantially lower gene counts (assumed unhealthy cells and/or data produced from poor emulsion quality).

Clustering of spinal cord neurons was done using 90 PCs at resolution 4 and split the dataset into three major groups: Group 1 – the well-described cardinal neuron classes as Group 1 (dI1-dI6, v0-v3 and motor neurons); Group 2 – the presumptive dILA neurons; and Group 3 – the presumptive dILB neurons. As the dimensionality of this dataset was evidently quite high, we performed a preliminary clustering at an intermediate level between major groups and refined types in order to identify classes, or families, of neurons. The number of dILA and dILB families was determined by clustering at different resolutions and analyzing cluster markers, robustness, and similarity to mature spinal cord cell families in mice, monkeys, and humans (3–5). Six families in each dIL group were considered optimal. All dILA and dILB neurons were then clustered together using 150 PCs and annotated by family at resolution 32, whereas cardinal neuron classes were clustered separately using 180 PCs and annotated by class at resolution 32. Neurons were annotated by cluster and also by classes (families of dIL neurons or cardinal classes) and these annotations were re-introduced to the dataset comprising all spinal cord neurons. This version of the dataset was presented in main figures, and used as a reference to annotate both spatial transcriptomics data and single-nucleus RNA-seq data retrieved from Qiu and colleagues (described below) (49). At this final stage of processing, 94,048 spinal cord neurons were retained.

PHATE trajectory inference was performed using PHATE (46) (R library “phateR”), with the following parameters: knn = 20, gamma = 0, t = 100, and 90 PCs for dimensionality reduction and visualization. Cluster robustness was performed for all neurons at the level of family, as well as the refined types of dILA and dILB using Silhouette and LISI scores (R libraries “lisi” and “cluster”) and computed with 90 PCs each. Dendrograms for different neuronal subsets were generated on the basis of centroid distances. The primary dendrogram, including all refined neuron types, was constructed using 150 PCs (Fig. 1F). A second, family-level dendrogram—excluding “Hindbrain”-like, “Immature”, and “Other” neuron types—was generated using 90 PCs (Fig. S3). Additionally, dendrograms for the dILA and dILB separately (Fig. S3) were constructed using 90 PCs.

### Pseudotime analysis using scFATES and Palantir:

Trajectory analysis was performed using scFates (v1.1.1) (47) and Palantir (48). PCA embeddings (90PCs) were extracted from the preprocessed AnnData object and then were used to compute diffusion maps (n=50 components) via Palantir, followed by construction of a multiscale diffusion space (X_palantir). A principal tree was learned with scFates using the Palantir embedding (ppt method, 150 nodes, λ=100, σ=0.025, 200 steps, seed=1). The root node was manually defined, and pseudotime was computed along the tree (20 jobs, seed=42). The progression of neuronal cell types along the inferred trajectory was analyzed by calculating pairwise pseudotime distances between mean pseudotime values of each group using scipy.spatial.distance.pdist. The resulting distance matrix was visualized as a heatmap with a reversed *viridis* colormap, where smaller (closer) distances appear brighter.

### Single-cell RNA-seq - annotation transfer to published datasets:

Data from Qiu and colleagues were retrieved in order to examine how dIL families arise at precisely defined embryonic stages. We first generously extracted all CNS neurons from time points E11.0 to E16.75, using the annotations “CNS_neurons”, “Intermediate_neuronal_progenitors”, and “Neuroectoderm_and_glia” from the top-level metadata class “major_trajectory” defined by Qiu et al. (49). We subsequently excluded all evidently non-spinal cord neurons using a battery of genes as exclusion criteria listed here: the presence of *Emx1/Emx2*/*Lhx6*/*Lhx8/Dlx1/Dlx3/Dlx5/Fezf2/Tbr1/Tbr2/Otx2/Six3* (presumptive forebrain neurons), *Tfap2d* and excitatory *En1*+ cells (presumptive midbrain), *Tph2* (presumptive serotonergic), *Dbh* (presumptive noradrenergic), or Hox genes of groups 1-3 but not group 4 and beyond (presumptive hindbrain). After first-pass filtering, data from all time points between E11.0 to E16.75 were integrated together by time point using Seurat v5 integration, and re-clustered using 120 PCs, and cell-type identity was predicted by transferring annotations from single-cell RNA-seq data generated here (see section Single-cell RNA-seq - data processing and Fig. 1) using 120 PCs. As post-integration clustering allowed clear separation of hindbrain and non-hindbrain cardinal classes by absence or presence of Hox group 4 genes and beyond, hindbrain clusters were further filtered using these criteria. From this point, a contingency table of predicted cell types was generated for each individual time point. Data were presented as the fraction of all dIL families and immature neurons present at each specific time point (done separately for dILA and dILB neurons).

### Fine time resolution fraction of cell types:

To assess whether the time course of cell type proportions from Qiu et al (49) varied over a finer temporal resolution, we applied a different statistical approach, motivated by the lack of distinct replicate time series. Here, we used Dynamic Time Warping (‘dtw’ package in R) to calculate a relative distance in the proportion-versus-time curves for every pair of cell types. Dynamic Time Warping is more sensitive than straightforward correlation because it takes into account the inherent ordering of time series data. To assess statistical significance of the distance between the curves for two distinct cell types, we ran 100,000 permutations of shuffled data and calculated the p-value as the proportion of permutations whose distance was greater than the measured distance (Table S12). Note that this is a two-sided test: P-values close to 1 indicate that the measured distance is significantly less than expected, whereas p-values close to 0 indicate that the measured distance is significantly greater.

### Single-cell RNA-seq – examination of published data:

To examine onset of Zic gene expression in dIL neurons, single-cell RNA-seq data from Delile et al. (31) consisting of only spinal cord neurons at time points E9.5, E10.5, E11.5, E12.5, and E13.5. To examine ranked expression of *Zic1* among pdL progenitors, neural progenitor-annotated data were retrieved from Delile et al. (31) from time points E11.5 and E12.5 (both time points of which are confidently during dIL neurogenesis and beyond dI4/dI5 neurogenesis); to enrich pdL progenitors, ventral progenitors (*Dbx2*, *Nkx6-1*, *Nkx2-2*, and *Olig2*) and dI1-3 progenitors (*Olig3*) were excluded by their respective marker sets. Remaining pdL progenitors expressed *Gsx1*, *Gsx2* and *Ascl1*, as expected.

### Spatial transcriptomics - data collection:

All tissues used for spatial transcriptomics were immersion fixed for 20-24 h. To determine the spatial arrangement of dorsal horn neuron subtypes in normal and mutant spinal cords we used the 10X Genomics Xenium (v1) platform. As much of the mutant tissue analyzed here was fixed-frozen, and as Xenium v1 did not yet have a supported fixed frozen tissue protocol, we followed the guide for fresh-frozen tissues with modifications detailed below. Cryosectioning was done as described in CG000579 revision C (10X Genomics) and fixation and permeabilization was done as described in CG000581 revision C (10X Genomics), except that the 30-minute incubation in fixation solution was omitted. Tissue chemistry was done as described in CG000582 revision F (10X Genomics). Probe-sets used were the v1 mouse brain panel (248 genes) plus 100 custom probes (Table S13) marking either classes or individual clusters from the single-cell RNA-seq dataset collected here. A reference dataset was produced using four wildtype E16.5 spinal cords spanning upper cervical to sacral segments, to most accurately capture the variation of the dorsal horn across segments and animals, and these comprised the first of two slides. We sought to capture a variety of mutant tissues on the second slide. Operation of the Xenium Analyzer instrument was done as described in CG000584 revision E (10X Genomics). Sections were grouped by animal or genotype during Xenium analyzer setup, and these data are also noted in table S13. Areas that contained overlapping sections were omitted from this step and were not further analyzed.

### Spatial transcriptomics - data processing:

Raw 10X Xenium datasets obtained from the Xenium analyzer computer were imported into Seurat, cells with zero transcripts were removed, all cells in a group were clustered using 60 PCs at 0.5 resolution, and Seurat objects were written as .RDS files (see code Xenium_2024_1_FilterClusterExport on Github). Subsequently, coordinates were assigned bounding each section, clusters and gene markers were displayed on a UMAP reduction, and non-neuronal and sensory neuron clusters were excluded from further processing (see code Xenium_2024_2_DefineROI_ExtractNeurons on Github). From filtered spinal neurons, annotations were predicted using Robust Cell-Type Decomposition (RCTD) (54) from the R package spacexr and our single-cell RNA-seq annotations (described above) as reference. On the basis of predicted cell-types, family-level metadata were assigned to each cell. Further, a section identifier, and an estimated spinal segment were added as metadata. Additional exclusion of non-neuronal clusters was done here, particularly for clusters that had widely indistinct or sparse annotation transfer; on inspection, these were often clusters with most cells bearing both neuronal and non-neuronal markers resulting from poor cell segmentation or minor cell overlay. Cell-type proportions were also examined here separately per spinal cord zone (C1-C4 – Cervical, C5-C8 – Brachial, Thoracic, L1-L3 – Upper lumbar, L4-L6 – Lower lumbar or Sacral); to ensure proportions were not affected by exclusion of particular parts of a section only complete sections or complete hemisections were included; from these, cell proportions are represented as a fraction of all neurons from the total sections or hemisections examined of a single embryo. Finally, cell coordinates and appropriate metadata were exported as .csv files for localization analysis (see code Xenium_2024_3_RCTD on Github for above processing steps). Subsequently, anatomical landmarks to be used as anchors for coordinate normalization were identified for each individual section retained at this stage. Coordinates from all sections of a given genotype and/or spinal segment were combined and overlaid on each other to best visualize neuron-type location across sections. These coordinates were subsequently exported as scatter plots for figures (see code Xenium_2024_4_Coordinate_processing_overlay on Github for above processing steps). To illustrate the relative dorso-ventral frequency of neurons from each dIL family (Fig. 3C), the normalized y-coordinate of all dIL neurons was assigned to one of 200 bins spanning the full extent of both dorsal and ventral horns. Histograms were smoothed (using Ggplot2 stat_smooth) and plotted as frequency (x-axis) and dorsoventral bin order (y-axis).

Data are made available as raw Xenium outputs, Seurat objects containing all section regions of interest (ROIs) filtered to remove non-neurons, normalized collated cell coordinates from all sections within the indicated segmental zone, and (for selected analyses) mirrored normalized collated cell coordinates from both wild-type (coordinates x<0) and null (coordinates x>0) embryos with wild-type cells downsampled to approximately match the number of null cells (numbers of each predicted cell-type were downsampled on the basis of the ratio of total null to total wild-type neurons).

### Spatial transcriptomics – statistics:

To understand changes in cell-type frequency between genotypes, contigency tables of predicted cell-types from E16.5 wild-type, *Zic1/4*-null and *Zic3*-null spinal cords were generated separately for each biological replicate and then normalized to the number sections collected per replicate. Selected cell-type frequencies were compared across all three groups in a two-way ANOVA (cell-type x genotype), and the post-hoc Tukey’s multiple comparisons test was done between all genotypes for each cell-type.

### Zic gene family expression distribution – statistics:

Gene expression distribution characterization: To assess the similarity in the curve shapes for gene expression across Zic genes, we performed a similar analysis as above, using Dynamic Time Warping to calculate curve-to-curve distances between pairs of genes, and permutations to assess statistical significance. Note that all pairwise distances have a p-value=1, indicating that all curves are “closer” to each other than expected by chance.

## Supplementary Material

Supplemental Figures S1 to S24

table-Table_S1

table-Table_S2

table-Table_S3

table-Table_S4

table-Table_S5

table-Table_S6

table-Table_S7

table-Table_S8

table-Table_S9

table-Table_S10

table-Table_S11

table-Table_S12

table-Table_S13

table-Table_S14

table-Table_S15

## Data Availability

No unique materials were generated herein this study. Sources of previously published reagents and mouse lines are noted in Ttable S15. All raw and processed single-cell RNA-sequencingRNA-seq and spatial transcriptomics data are available on in the Gene Expression Omnibus (GSE291462). All original analysis code is available for download at https://github.com/ArielLevineLabNINDS/Dorsal_Horn.
