# Supplementary material for: Ontogeny of the spinal cord dorsal horn
Source: Science. Author manuscript; Available in PMC 2026 Feb 6. (PMC12879194; doi:10.1126/science.adx5781)
Supplement: Supplemental Figures S1 to S24 [file NIHMS2135978-supplement-Supplemental_Figures_S1_to_S24.pdf]

Science
AAAS

Supplementary Materials for

Ontogeny of the spinal cord dorsal horn

Authors: Robert Brian Roome[1], Archana Yadav[2], Lydia Flores[3], Amrit Puarr[4], Diana Nardini[5], Alexander Richardson[1], Ronald R. Waclaw[5,6], Ruth M. Arkell[4], Vilas Menon[2], Jane E. Johnson[3,7], Ariel J. Levine[1,8,*]

Corresponding author: ariel.levine@nih.gov

The PDF file includes:

Figs S1 to S24

Other Supplementary Materials for this manuscript include the following:

Tables S1 to S15

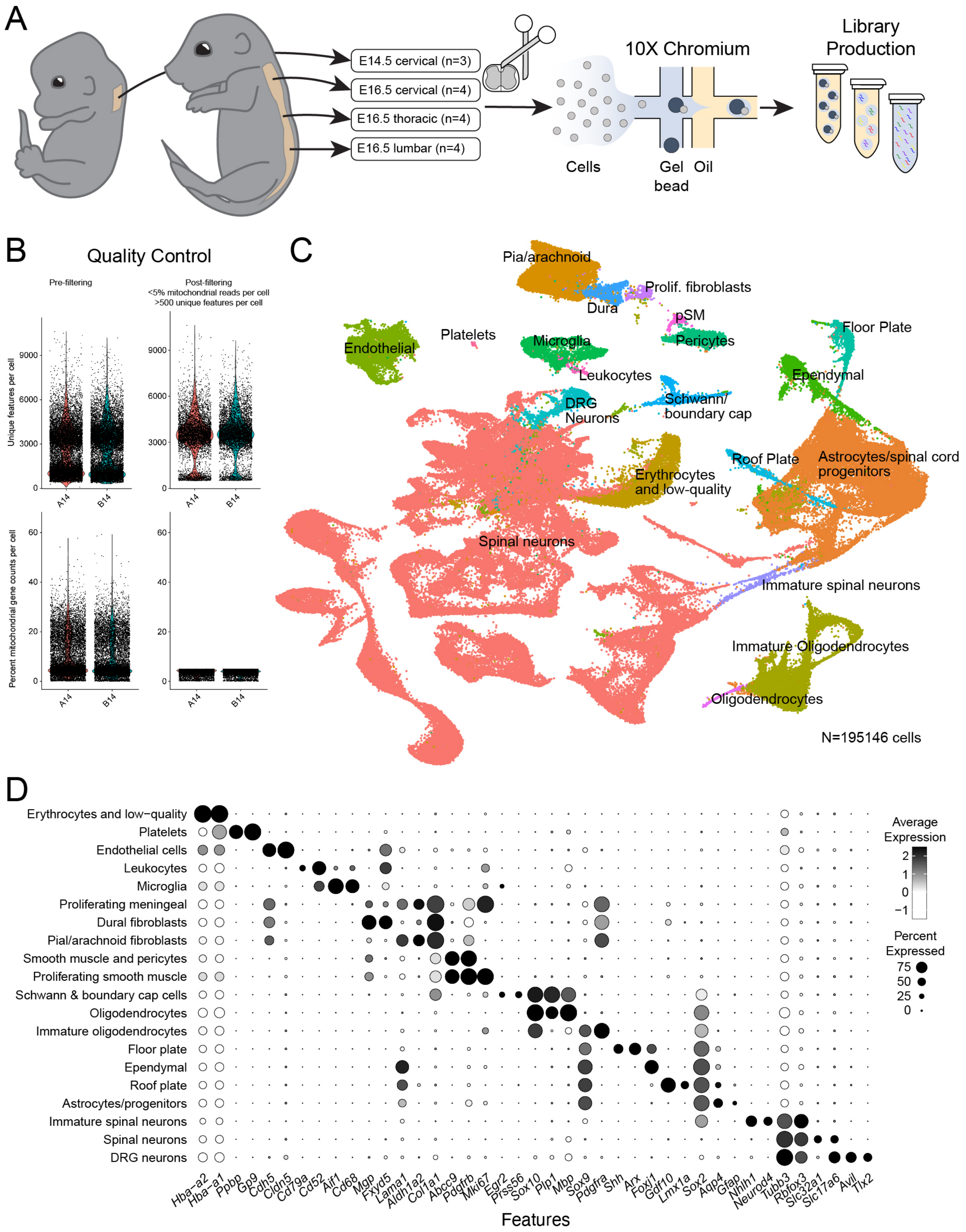
A
E14.5 cervical (n=3)
E16.5 cervical (n=4)
E16.5 thoracic (n=4)
E16.5 lumbar (n=4)
10X Chromium
Cells
Gel bead
Oil
Library Production
B
Quality Control
Pre-filtering
Post-filtering
<5% mitochondrial reads per cell
>500 unique features per cell
Unique features per cell
Percent mitochondrial gene counts per cell
A14
B14
C
Pia/arachnoid
Dura
Prolif. fibroblasts
pSM
Pericytes
Floor Plate
Endothelial
Platelets
Microglia
Leukocytes
Ependymal
DRG Neurons
Schwann/ boundary cap
Roof Plate
Astrocytes/spinal cord progenitors
Erythrocytes and low-quality
Spinal neurons
Immature spinal neurons
Immature Oligodendrocytes
Oligodendrocytes
N=195146 cells
D
Erythrocytes and low−quality
Platelets
Endothelial cells
Leukocytes
Microglia
Proliferating meningeal
Dural fibroblasts
Pial/arachnoid fibroblasts
Smooth muscle and pericytes
Proliferating smooth muscle
Schwann & boundary cap cells
Oligodendrocytes
Immature oligodendrocytes
Floor plate
Ependymal
Roof plate
Astrocytes/progenitors
Immature spinal neurons
Spinal neurons
DRG neurons
Hba−a2 Hba−a1 Ppbp Gp9 Cdh5 Cldn5 Cd79a Cd52 Aif1 Cd68 Mgp Fxyd5 Lama1 Aldh1a2 Col1a1 Abcc9 Pdgfrb Mki67 Egr2 Prss56 Sox10 Plp1 Mbp Sox9 Pdgfra Shh Arx Foxj1 Gdf10 Lmx1a Sox2 Aqp4 Gfap Nhlh1 Neurod4 Tubb3 Rbfox3 Slc32a1 Slc17a6 Avil Tlx2
Features
Average Expression
Percent Expressed

Fig. S1: Generation of embryonic spinal cord single-cell RNA-sequencing data.

(**A**) Spinal cords were extracted from E14.5 and E16.5 spinal cords, dissociated, run through the 10X Chromium 3' gene expression pipeline, and libraries were produced and sequenced. (**B**) After importation into Seurat, poor quality cells with low unique features or a high percentage of mitochondrial gene reads were removed from the dataset. (**C**) UMAP plot of the high-quality cells, following integration and clustering. (**D**) Dot plot showing all major annotated spinal cord cell types, showing marker gene sets used to classify clusters into archetypal spinal cord cell types. Dot size represents the fraction of a cell type expressing a gene, and dot shading represents the average normalized expression of a gene per cell type.
Numbers: Pooled embryonic spinal cord replicates (A): E14.5 cervical spinal cord (n=3), E16.5 cervical spinal cord (n=4), E16.5 thoracic spinal cord (n=4), E16.5 lumbosacral spinal cord (n=4). Total cells after quality control: N=195146.
Abbreviations: DEG – differentially-expressed gene, UMAP – uniform manifold approximation and projection.

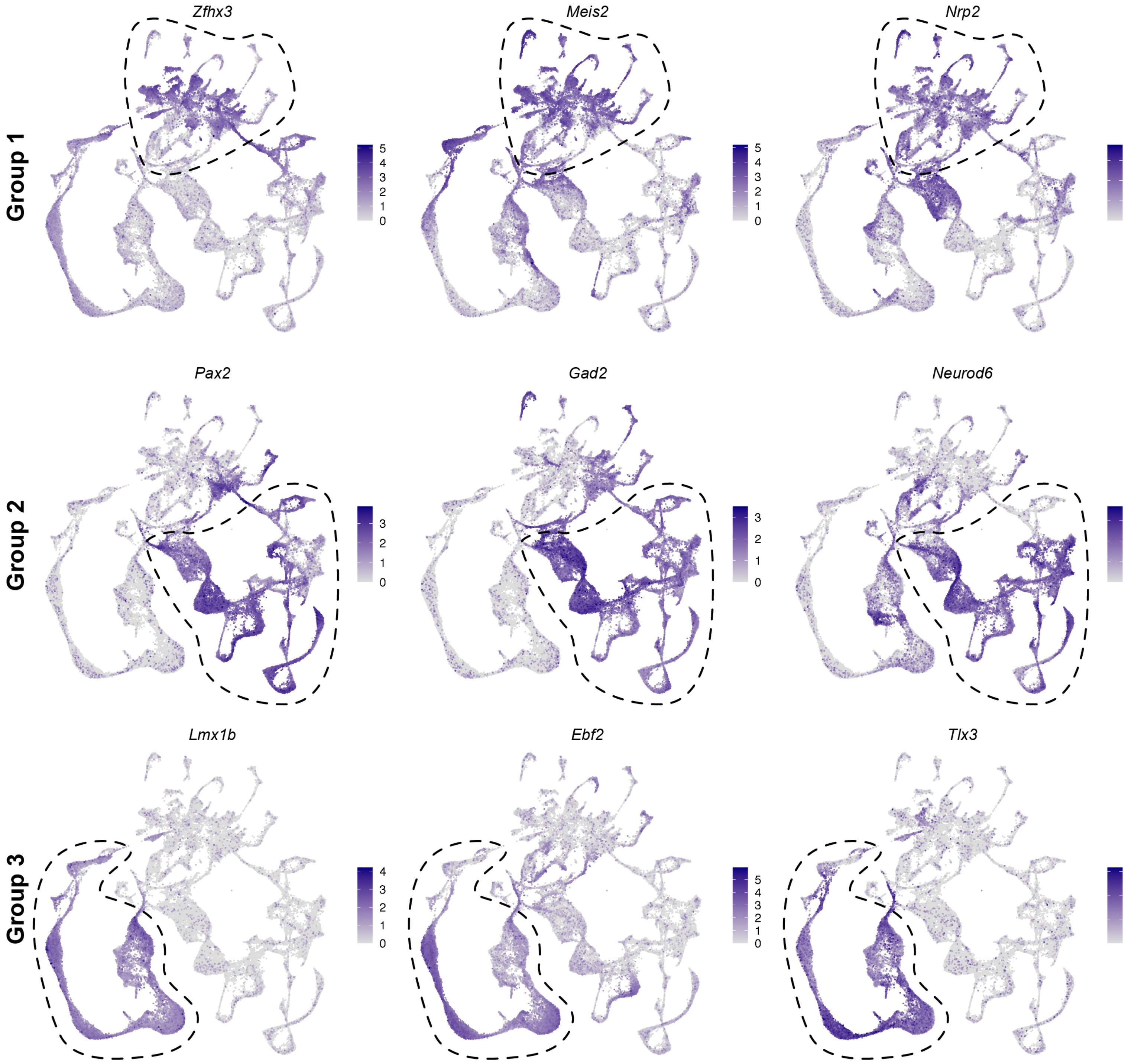

Zfhx3
Meis2
Nrp2
Group 1
5
4
3
2
1
0
Pax2
Gad2
Neurod6
Group 2
3
2
1
0
Lmx1b
Ebf2
Tlx3
Group 3
4
3
2
1
0
5
4
3
2
1
0

Fig. S2: Top differentially-expressed genes of spinal neuron groups 1, 2 and 3.

UMAP plots here depict top identifying differentially-expressed genes from spinal neuron groups 1, 2 and 3 in rows 1, 2 and 3 respectively, indicated within the perimeter of dashed lines. Cell shade depicts log-normalized gene expression levels (see legends). All cells and their UMAP coordinates are the same as presented in Figure 1B.

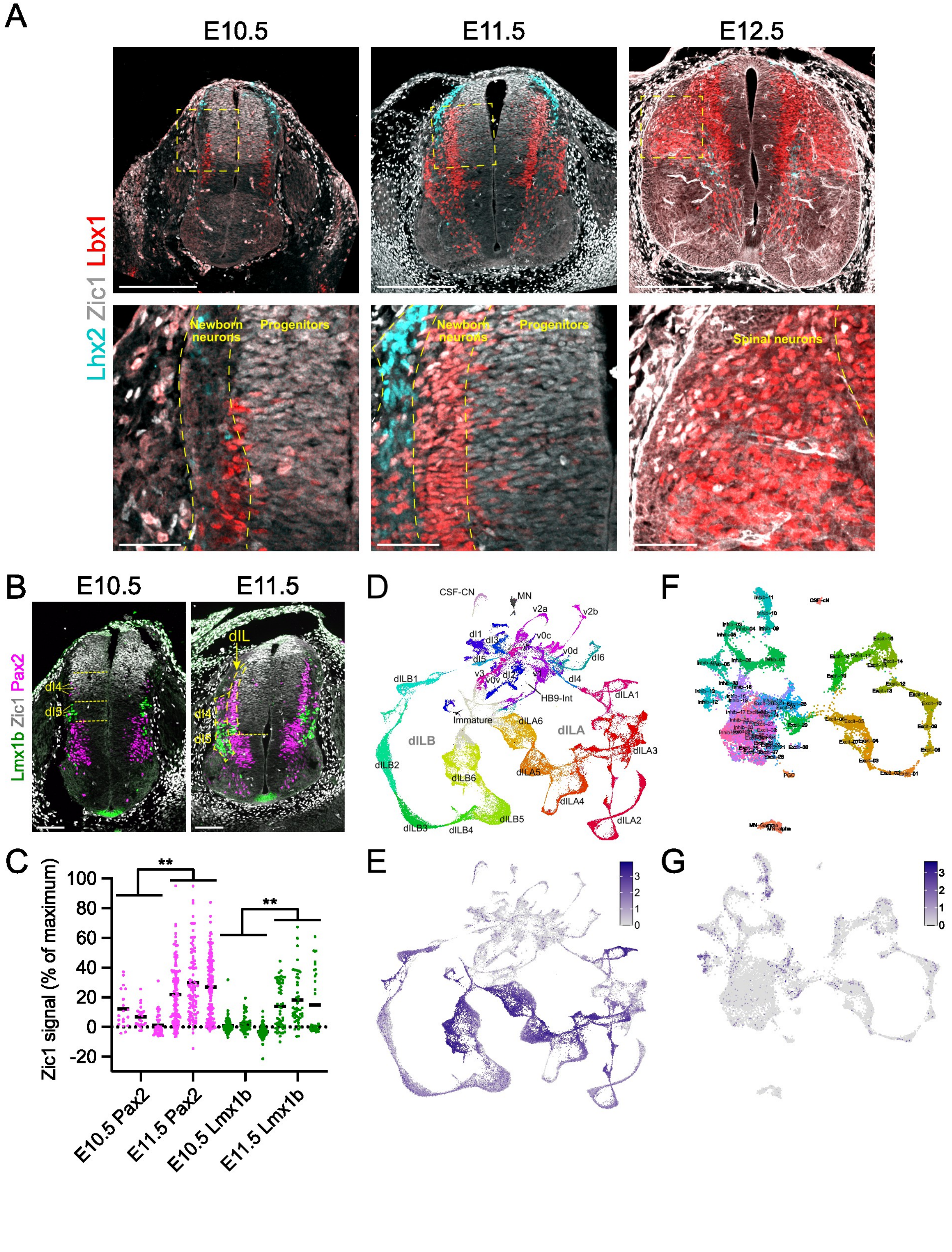
A
E10.5
E11.5
E12.5
Lhx2 Zic1 Lbx1
Newborn neurons
Progenitors
Spinal neurons
B
E10.5
E11.5
Lmx1b Zic1 Pax2
dI4
dI5
dIL
C
Zic1 signal (% of maximum)
100
80
60
40
20
0
-20
**
E10.5 Pax2
E11.5 Pax2
E10.5 Lmx1b
E11.5 Lmx1b
D
CSF-CN
MN
v2a
v2b
dI1
dI3
v0c
v0d
dI5
dI6
v3
v0v
dI2
dI4
HB9-Int
dILA1
dILB1
Immature
dILA6
dILA
dILB
dILA3
dILB2
dILB6
dILA5
dILA4
dILA2
dILB3
dILB4
dILB5
E
F
G

Fig. S3: Zic1 expression defines the boundary of dI4/dILA and dI5/dILB.

(**A**) Expression of Zic1 in embryonic day (E) 10.5, E11.5 and E12.5 spinal cords. Zic1 is expressed in dorsal progenitors throughout neurogenesis, appears in newborn dIL neurons (Lbx1+) at E11.5, and is expressed in varying degrees throughout dIL neurons at E12.5. Notably it is absent from dI4-6 neurons marked by Lbx1 at E10.5, as well as dI1 neurons marked by Lhx2. (B) Expression of Zic1 at E10.5 and E11.5 together with Pax2 (dI4, dILA) and Lmx1b (dI5, dILB). (C) Quantification of intensity of Zic1 immunohistochemistry within individual Pax2+ or Lmx1b+ newborn neurons, demonstrating an increase in Zic1 expression in newborn neurons at E11.5. (D-G) UMAP plots of neurons from single-cell RNA-sequencing data derived from E14.5-E16.5 (Fig 1B; D-E) or postnatal (Russ et al. 2021, F-G) spinal cords, showing either cluster annotations (D,F) or *Zic1* expression (E,G; point shading depicts log-normalized gene expression – see legend).

Numbers: (A,B) Representative images from n=3 spinal cords from E10.5, E11.5 and E12.5.

Statistics: Unpaired t-test. **$p<0.01$

Single-cell RNA-sequencing cells (F,G): 19,353 cells (Russ et al., 2021)

Scale bars: (A) 250 μm (top row), 50 μm (bottom row); (B) 250 μm.

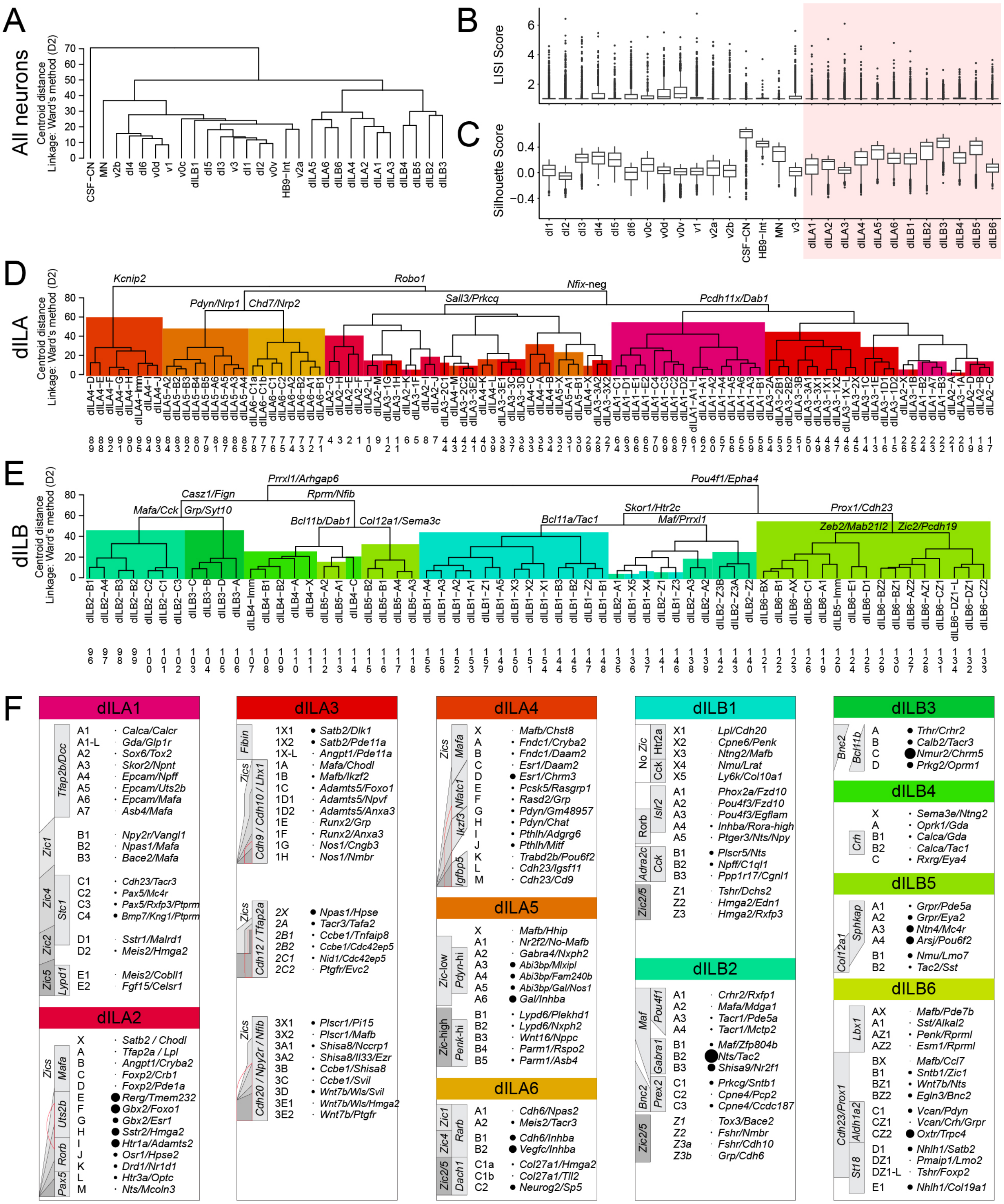
A
All neurons
B
LISI Score
C
Silhouette Score
D
dILA
E
dILB
F
dILA1
dILA2
dILA3
dILA4
dILA5
dILA6
dILB1
dILB2
dILB3
dILB4
dILB5
dILB6

Fig. S4: Transcriptomic analysis of post-neurogenesis embryonic spinal cord neurons.

(**A**) Hierarchical relationships of all major spinal cord neuron classes by cluster centroid distance in PC space. Dendrograms were produced by Ward's method (D2), with use of the top 90 PCs. **(B,C)** Cluster robustness metrics for major spinal cord neuron classes, using LISI (**B**) and silhouette (**C**) scores, with dIL neuron families highlighted in red. **(D,E)** Hierarchical relationships of dILA (**D**) neuron subtypes and dILB (**E**) neuron subtypes within each respective class by cluster centroid distance in PC space. Dendrograms were produced by Ward's method (D2), with use of 90 PCs in both cases. Colors represent major dILA and dILB families. High-order branches are annotated with top DEGs. Numbers refer to terminal dendrogram branches in Fig. 1F which corresponding to subtype names indicated on terminal dendrogram branches here. (**F**) Annotated tables for each of twelve dIL families, showing subtypes and top marker genes. The "-L" suffix represents neuron subtypes which are virtually only found samples taken from the lumbosacral cord; no outstanding neuron subtypes are exclusive to cervical or thoracic segments given the current number of dIL neurons sampled. Dot size next to subtype name corresponds to the relative frequency of dIL neuron subtype among subtypes of all six families (excluding clusters annotated as transitional or immature (those overrepresented in E14.5 data or with substantial *Robo3*/*Nhlh1* expression).

Numbers: Single-cell RNA-sequencing data analyzed here was derived from 94,048 neurons (see Fig. 1B and methods for details).

Abbreviations: DEG – differentially-expressed gene, LISI – Local Inverse Simpson's Index, PC – principal component

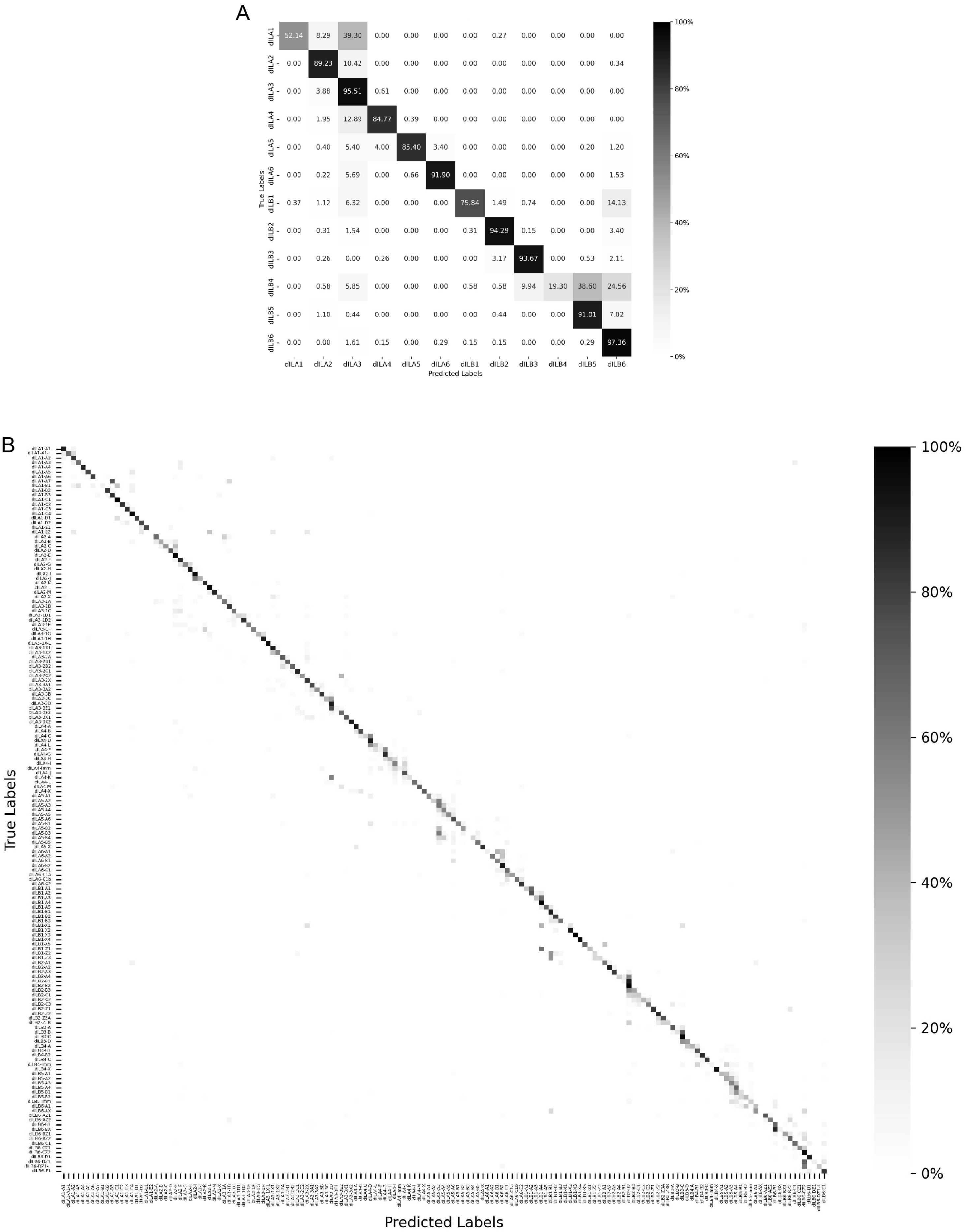
A
dILA1 52.14 8.29 39.30 0.00 0.00 0.00 0.00 0.27 0.00 0.00 0.00 0.00
dILA2 0.00 89.23 10.42 0.00 0.00 0.00 0.00 0.00 0.00 0.00 0.00 0.34
dILA3 0.00 3.88 95.51 0.61 0.00 0.00 0.00 0.00 0.00 0.00 0.00 0.00
dILA4 0.00 1.95 12.89 84.77 0.39 0.00 0.00 0.00 0.00 0.00 0.00 0.00
dILA5 0.00 0.40 5.40 4.00 85.40 3.40 0.00 0.00 0.00 0.00 0.20 1.20
dILA6 0.00 0.22 5.69 0.00 0.66 91.90 0.00 0.00 0.00 0.00 0.00 1.53
dILB1 0.37 1.12 6.32 0.00 0.00 0.00 75.84 1.49 0.74 0.00 0.00 14.13
dILB2 0.00 0.31 1.54 0.00 0.00 0.00 0.31 94.29 0.15 0.00 0.00 3.40
dILB3 0.00 0.26 0.00 0.26 0.00 0.00 0.00 3.17 93.67 0.00 0.53 2.11
dILB4 0.00 0.58 5.85 0.00 0.00 0.00 0.58 0.58 9.94 19.30 38.60 24.56
dILB5 0.00 1.10 0.44 0.00 0.00 0.00 0.00 0.44 0.00 0.00 91.01 7.02
dILB6 0.00 0.00 1.61 0.15 0.00 0.29 0.15 0.15 0.00 0.00 0.29 97.36
dILA1 dILA2 dILA3 dILA4 dILA5 dILA6 dILB1 dILB2 dILB3 dILB4 dILB5 dILB6
True Labels
Predicted Labels
100% 80% 60% 40% 20% 0%
B
True Labels
Predicted Labels
100% 80% 60% 40% 20% 0%

Fig. S5: Robustness of spinal neuron clusters using a random forest classifier.

Confusion matrix heatmaps demonstrating the accuracy of a trained random forest classifier in predicting either family-level (**A**) or family subtype-level (**B**) annotations for individual dIL neurons using single-cell RNA-sequencing data generated and annotated here.

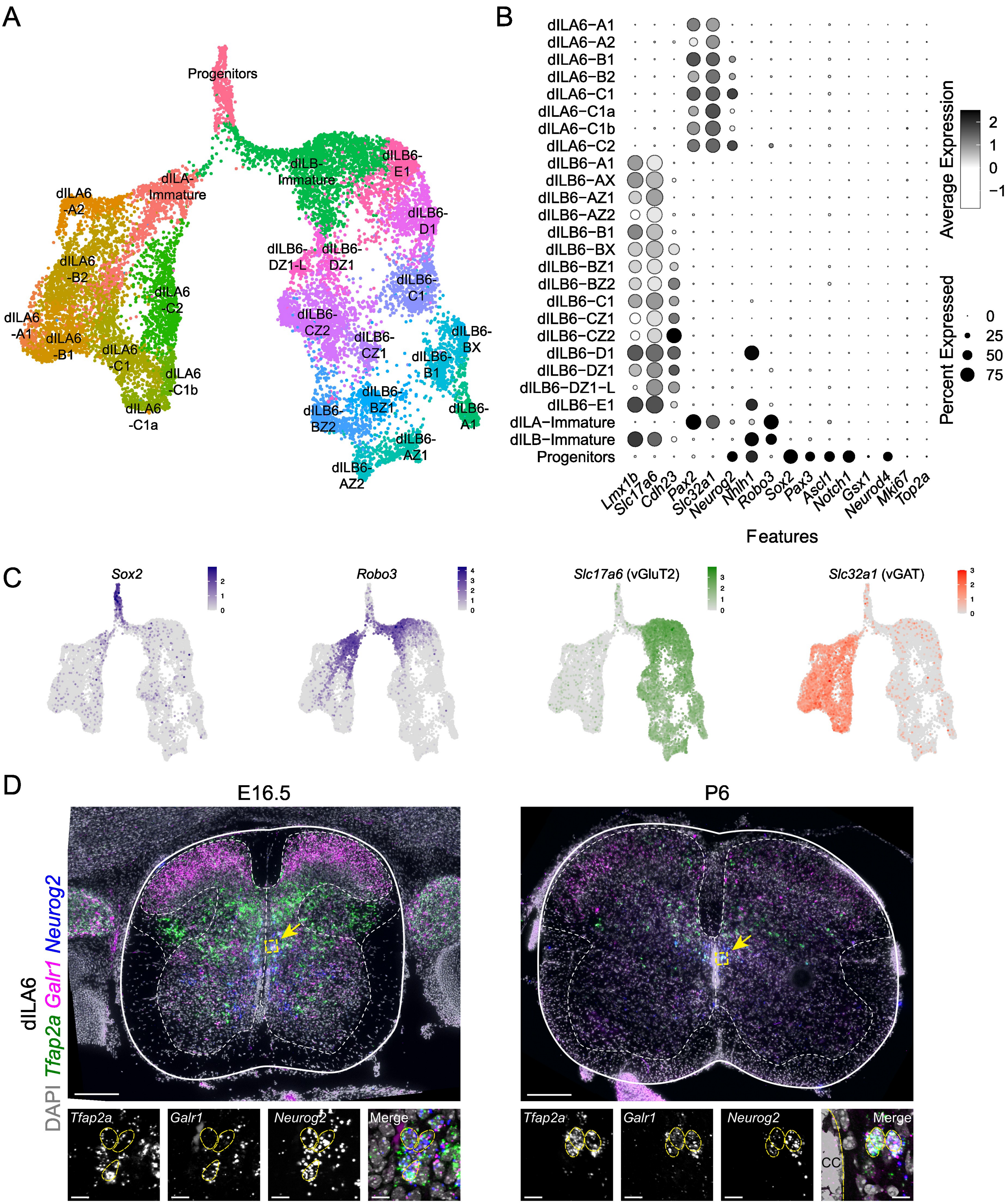

A
Progenitors
dILA-Immature
dILB-Immature
dILA6-A1
dILA6-A2
dILA6-B1
dILA6-B2
dILA6-C1
dILA6-C1a
dILA6-C1b
dILA6-C2
dILB6-E1
dILB6-D1
dILB6-DZ1-L
dILB6-DZ1
dILB6-C1
dILB6-CZ2
dILB6-CZ1
dILB6-BX
dILB6-B1
dILB6-BZ1
dILB6-BZ2
dILB6-A1
dILB6-AZ1
dILB6-AZ2
B
Average Expression
Percent Expressed
0
25
50
75
Lmx1b Slc17a6 Cdh23 Pax2 Slc32a1 Neurog2 Nhlh1 Robo3 Sox2 Pax3 Ascl1 Notch1 Gsx1 Neurod4 Mki67 Top2a
Features
C
Sox2
Robo3
Slc17a6 (vGluT2)
Slc32a1 (vGAT)
D
E16.5
P6
dILA6
DAPI Tfap2a Galr1 Neurog2
Tfap2a
Galr1
Neurog2
Merge
CC

Fig. S6: dILA6 and dILB6 are not transient cell-states.

(**A**) UMAP plot of dILA6 neurons, dILB6 neurons, immature dIL neurons as well as dorsal progenitors (a subset of single-cell RNA-sequencing data plotted in Figure 1B); a total of 14,436 cells are displayed here. UMAP was generated using 60PCs from PCA rerun specifically on this subset of neurons. Refined cell-type annotations used here and in (**B**) are described in detail in Figure S4. (**B**) Dotplot run on data from (**A**), showing that immature cells as well as refined cell types of dILA6 and dILB6 families do not express immature, progenitor, or cycling cell-related genes. (**C**) UMAP plots from (A), depicting the expression of progenitor marker *Sox2*, immature neuron marker *Robo3*, glutamatergic marker *Slc17a6* (vGluT2) and gabaergic marker *Slc32a1* (vGAT). Legends indicate log-normalized gene expression. (**D**) Photomicrographs show E16.5 (left) and postnatal day (P)6 (right) cervical spinal cords stained for expression of the dILA6 marker set *Tfap2a*, *Galr1* and *Neurog2*. White solid and dashed white lines indicate approximate anatomical boundaries of the spinal cord, except boxes marked by dashed yellow lines, which indicate the corresponding magnified fields of view in the bottom row. Yellow dashed circles in the bottom row show the boundaries of nuclei from cells expressing the dILA6 marker gene set. The central canal is marked by a yellow dashed line in magnified images from the P6 spinal cord for orientation purposes.

Numbers: (**A-C**) Single-cell RNA-sequencing data analyzed here was derived from 14,436 neurons (themselves derived from Fig. 1B). (**D**) n>3 E16.5 spinal cords; n=12 P6 spinal cords.

Scale bars: (**D**) 500 μm; 25 μm for magnified panels.

Abbreviations: CC – central canal, PC – principal component, UMAP – uniform manifold approximation and projection.

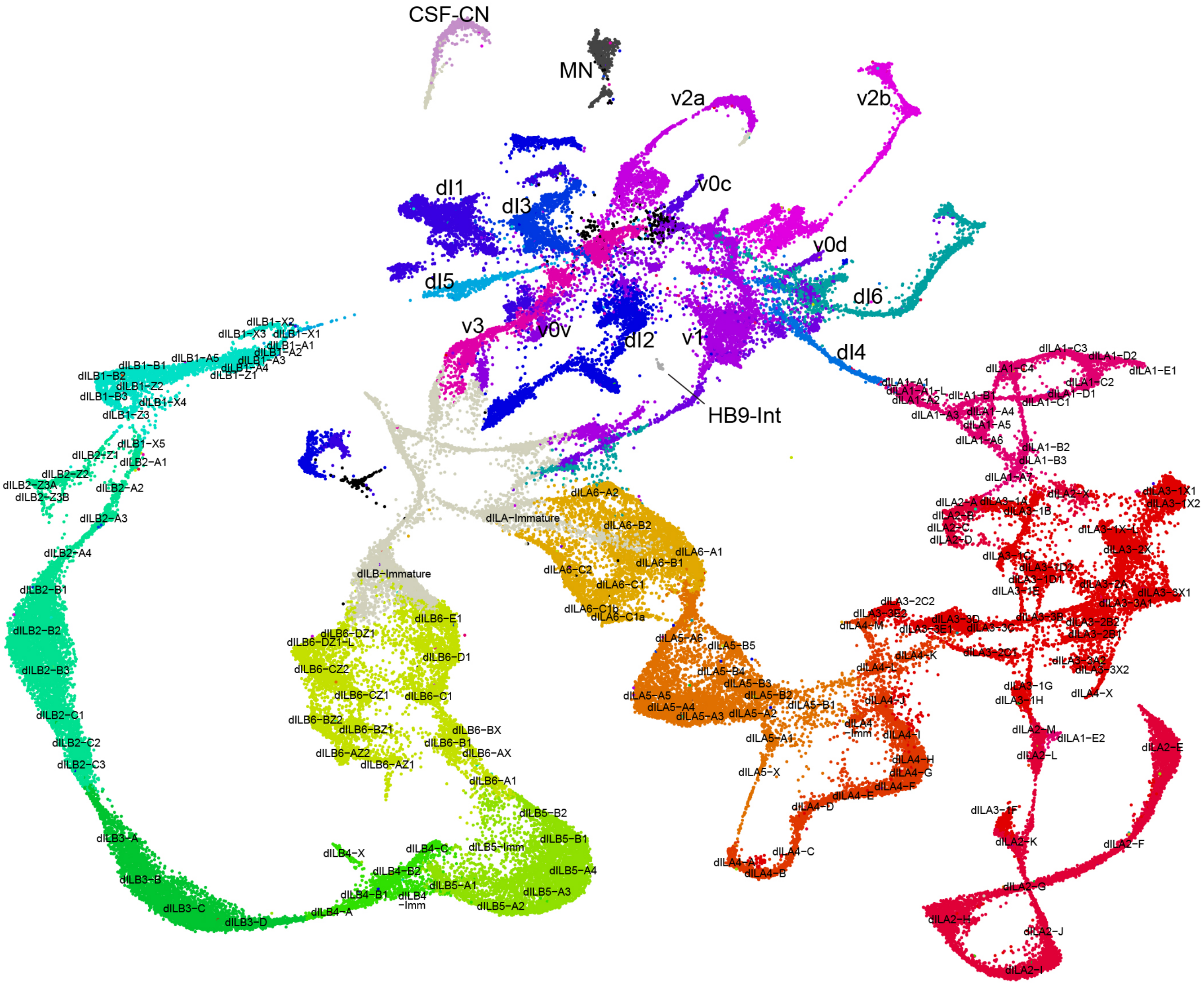

CSF-CN
MN
v2a
v2b
dI1
dI3
v0c
v0d
dI5
dI6
v3
v0v
dI2
v1
dI4
HB9-Int
dILB1-X2
dILB1-X3
dILB1-X1
dILB1-A1
dILB1-A2
dILB1-A3
dILB1-A4
dILB1-A5
dILB1-B1
dILB1-B2
dILB1-Z2
dILB1-B3
dILB1-X4
dILB1-Z1
dILB1-Z3
dILB1-X5
dILB2-Z1
dILB2-A1
dILB2-Z2
dILB2-Z3A
dILB2-Z3B
dILB2-A2
dILB2-A3
dILB2-A4
dILB2-B1
dILB2-B2
dILB2-B3
dILB2-C1
dILB2-C2
dILB2-C3
dILB3-A
dILB3-B
dILB3-C
dILB3-D
dILB4-A
dILB4-B1
dILB4-B2
dILB4-X
dILB4-C
dILB4
-Imm
dILB5-A1
dILB5-A2
dILB5-A3
dILB5-A4
dILB5-B1
dILB5-B2
dILB5-Imm
dILB6-A1
dILB6-AX
dILB6-B1
dILB6-BX
dILB6-C1
dILB6-D1
dILB6-E1
dILB6-DZ1
dILB6-DZ1-L
dILB6-CZ2
dILB6-CZ1
dILB6-BZ2
dILB6-BZ1
dILB6-AZ2
dILB6-AZ1
dILB-Immature
dILA-Immature
dILA6-A2
dILA6-B2
dILA6-A1
dILA6-B1
dILA6-C2
dILA6-C1
dILA6-C1b
dILA6-C1a
dILA5-A6
dILA5-B5
dILA5-B4
dILA5-B3
dILA5-B2
dILA5-B1
dILA5-A5
dILA5-A4
dILA5-A3
dILA5-A2
dILA5-A1
dILA5-X
dILA4-M
dILA4-K
dILA4-L
dILA4-J
dILA4-I
dILA4
-Imm
dILA4-H
dILA4-G
dILA4-F
dILA4-E
dILA4-D
dILA4-C
dILA4-A
dILA4-B
dILA4-X
dILA1-C3
dILA1-D2
dILA1-C4
dILA1-E1
dILA1-C2
dILA1-D1
dILA1-A1
dILA1-C1
dILA1-A4
dILA1-A5
dILA1-B2
dILA1-B3
dILA1-A7
dILA1-E2
dILA3-1X1
dILA3-1X2
dILA3-2X
dILA3-3X1
dILA3-3X2
dILA3-3A1
dILA3-2C2
dILA3-2B2
dILA3-2B1
dILA3-1G
dILA3-1H
dILA3-1F
dILA2-M
dILA2-L
dILA2-E
dILA2-F
dILA2-K
dILA2-G
dILA2-H
dILA2-J
dILA2-I

Fig. S7: UMAP plot of refined dIL family neuron subtypes.

UMAP plot of all spinal cord neurons, with the approximate UMAP centroid of each dIL neuron type annotated. Cardinal-class neurons are annotated in larger text.
Abbreviations: UMAP – uniform manifold approximation and projection.

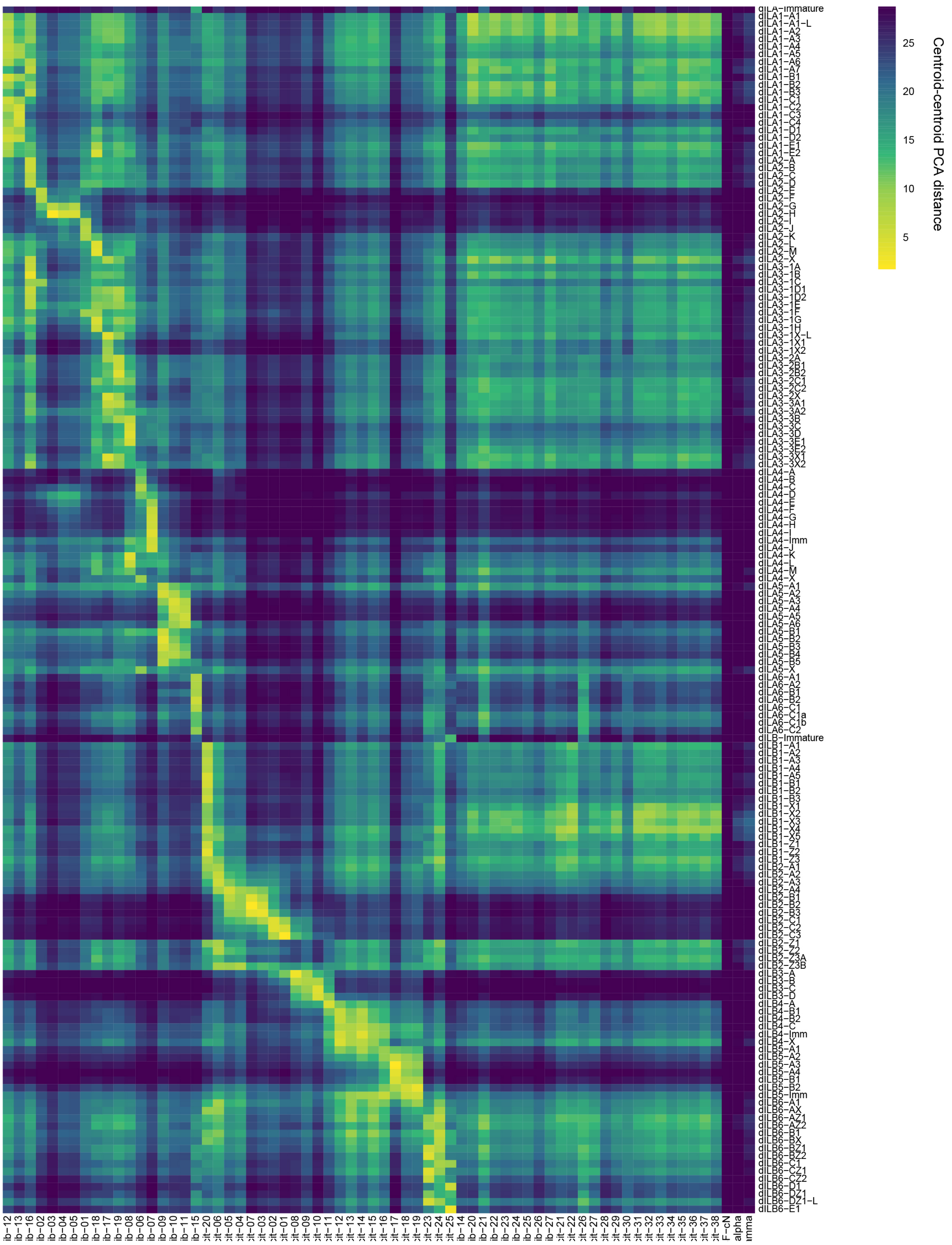

Centroid-centroid PCA distance
5
10
15
20
25
dILA−Immature
dILA1−A1
dILA1−A1−L
dILA1−A2
dILA1−A3
dILA1−A4
dILA1−A5
dILA1−A6
dILA1−A7
dILA1−B1
dILA1−B2
dILA1−B3
dILA1−C1
dILA1−C2
dILA1−C3
dILA1−C4
dILA1−D1
dILA1−D2
dILA1−E1
dILA1−E2
dILA2−A
dILA2−B
dILA2−C
dILA2−D
dILA2−E
dILA2−F
dILA2−G
dILA2−H
dILA2−I
dILA2−J
dILA2−K
dILA2−L
dILA2−M
dILA2−X
dILA3−1A
dILA3−1B
dILA3−1C
dILA3−1D1
dILA3−1D2
dILA3−1E
dILA3−1F
dILA3−1G
dILA3−1H
dILA3−1X−L
dILA3−1X1
dILA3−1X2
dILA3−2A
dILA3−2B1
dILA3−2B2
dILA3−2C1
dILA3−2C2
dILA3−2X
dILA3−3A1
dILA3−3A2
dILA3−3B
dILA3−3C
dILA3−3D
dILA3−3E1
dILA3−3E2
dILA3−3X1
dILA3−3X2
dILA4−A
dILA4−B
dILA4−C
dILA4−D
dILA4−E
dILA4−F
dILA4−G
dILA4−H
dILA4−I
dILA4−Imm
dILA4−J
dILA4−K
dILA4−L
dILA4−M
dILA4−X
dILA5−A1
dILA5−A2
dILA5−A3
dILA5−A4
dILA5−A5
dILA5−A6
dILA5−B1
dILA5−B2
dILA5−B3
dILA5−B4
dILA5−B5
dILA5−X
dILA6−A1
dILA6−A2
dILA6−B1
dILA6−B2
dILA6−C1
dILA6−C1a
dILA6−C1b
dILA6−C2
dILB−Immature
dILB1−A1
dILB1−A2
dILB1−A3
dILB1−A4
dILB1−A5
dILB1−B1
dILB1−B2
dILB1−B3
dILB1−X1
dILB1−X2
dILB1−X3
dILB1−X4
dILB1−X5
dILB1−Z1
dILB1−Z2
dILB1−Z3
dILB2−A1
dILB2−A2
dILB2−A3
dILB2−A4
dILB2−B1
dILB2−B2
dILB2−B3
dILB2−C1
dILB2−C2
dILB2−C3
dILB2−Z1
dILB2−Z2
dILB2−Z3A
dILB2−Z3B
dILB3−A
dILB3−B
dILB3−C
dILB3−D
dILB4−A
dILB4−B1
dILB4−B2
dILB4−C
dILB4−Imm
dILB4−X
dILB5−A1
dILB5−A2
dILB5−A3
dILB5−A4
dILB5−B1
dILB5−B2
dILB5−Imm
dILB6−A1
dILB6−AX
dILB6−AZ1
dILB6−AZ2
dILB6−B1
dILB6−BX
dILB6−BZ1
dILB6−BZ2
dILB6−C1
dILB6−CZ1
dILB6−CZ2
dILB6−D1
dILB6−DZ1
dILB6−DZ1−L
dILB6−E1

Fig. S8: Correspondence of embryonic to postnatal refined neuron subtypes

Diagram illustrating the relationships between refined embryonic dIL neuron types and postnatal/adult refined neuron types from Russ et al., (2021) *(7)*. Color scale indicates centroid-centroid distance in principal component (PC) space of each respective pair of neuron type annotations between dIL neurons from Figure 1D and those from Russ et al., (2021), with yellow indicating stronger relationships and navy indicating weak or no relationships.

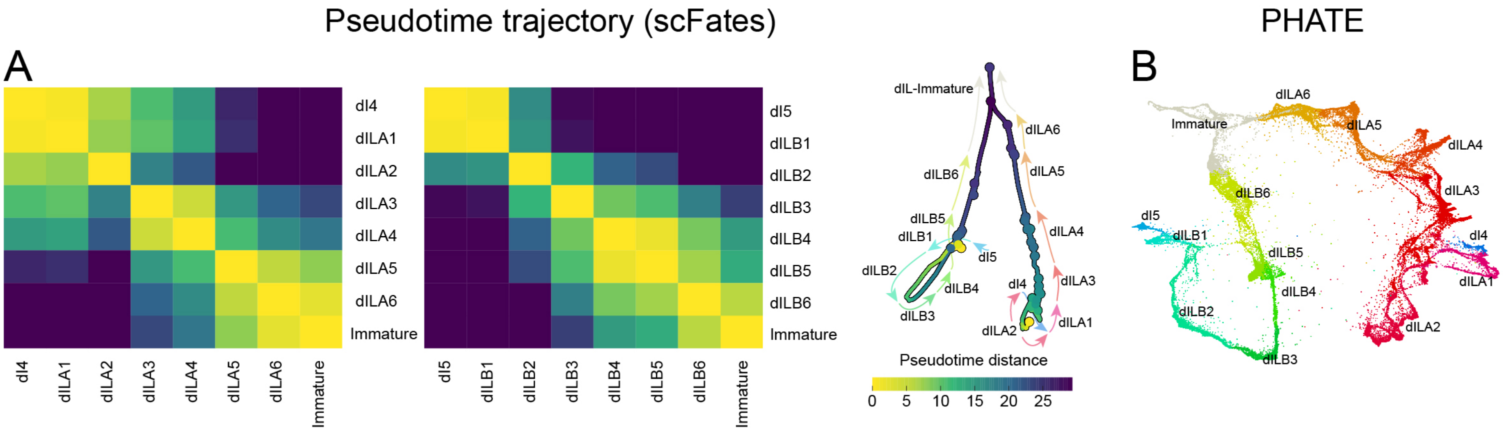
Pseudotime trajectory (scFates)
PHATE
A
B
dI4
dILA1
dILA2
dILA3
dILA4
dILA5
dILA6
Immature
dI5
dILB1
dILB2
dILB3
dILB4
dILB5
dILB6
Immature
dIL-Immature
Pseudotime distance
0 5 10 15 20 25

Fig. S9: Trajectory analysis of the progression of dIL family birth dates

A) Matrix showing pseudotime distance between dIL families, dI4, dI5 and immature neurons, calculated in scFates.
B) PHATE plot of single-cell RNA-sequencing data of dIL neurons, together with their early-born dI4/5 counterparts and immature neurons. This neuron set is the same as that used in (A). Numbers: 66,492 cells, a subset of those initially presented in Figure 1B.

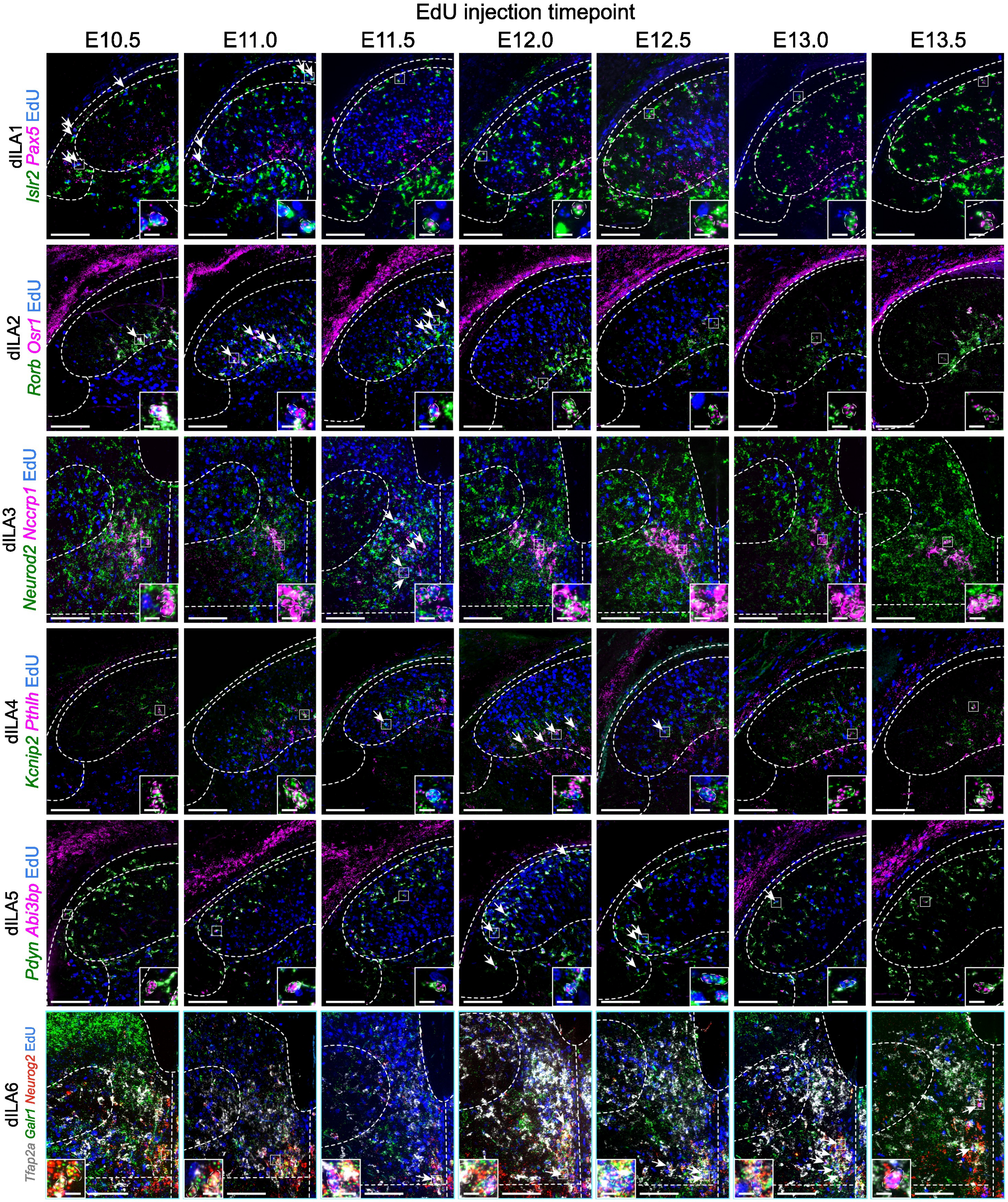

EdU injection timepoint
E10.5
E11.0
E11.5
E12.0
E12.5
E13.0
E13.5
dILA1 Islr2 Pax5 EdU
dILA2 Rorb Osr1 EdU
dILA3 Neurod2 Nccrp1 EdU
dILA4 Kcnip2 Pthlh EdU
dILA5 Pdyn Abi3bp EdU
dILA6 Tfap2a Galr1 Neurog2 EdU

Fig. S10: EdU birthdating of dILA families.

Representative images of the lateral dorsal horn of the spinal cord from E16.5 embryos having received EdU pulses at either E10.5, E11.0, E11.5, E12.0, E12.5, E13.0 or E13.5 respectively. A subset of dILA families are identified by coincident expression of two genes (magenta and green) revealed by RNA Scope in-situ hybridization which exclude all other dIL neuron families; dILA1 – *Islr2* and *Pax5*, dILA2 – *Rorb* and *Osr1*, dILA3 – *Neurod2* and *Nccrp1*, dILA4 – *Kcnip2* and *Pthlh*, dILA5 – *Pdyn* and *Abi3bp* and dILA6 (in which case grey, green and red are used) – *Tfap2a*, *Galr1* and *Neurog2*. Arrows indicate dILA family neurons expressing the corresponding marker gene set and which are labeled with nuclear EdU (blue). Dashed lines indicate the border of the superficial dorsal horn with the deep dorsal horn and the lateral spinal nucleus, the lateral spinal nucleus with the lateral funiculus, and the pia. Insets indicate examples of triple-positive neurons surrounded by dashed lines. Example images from this figure used in Fig. 2E are reused here for contextual purposes.

Numbers: Individual litters of embryos from a mother given a single EdU injection were considered a single biological replicate. E10.5 (n=5 litters), E11.0 (n=4 litters), E11.5 (n=5 litters), E12.0 (n=5 litters), E12.5 (n=5 litters), E13.0 (n=5 litters), E13.5 (n=4 litters).

Scale bars: 250 µm, 25 µm for inset panels.

Abbrevations: EdU - 5-ethynyl-2'-deoxyuridine.

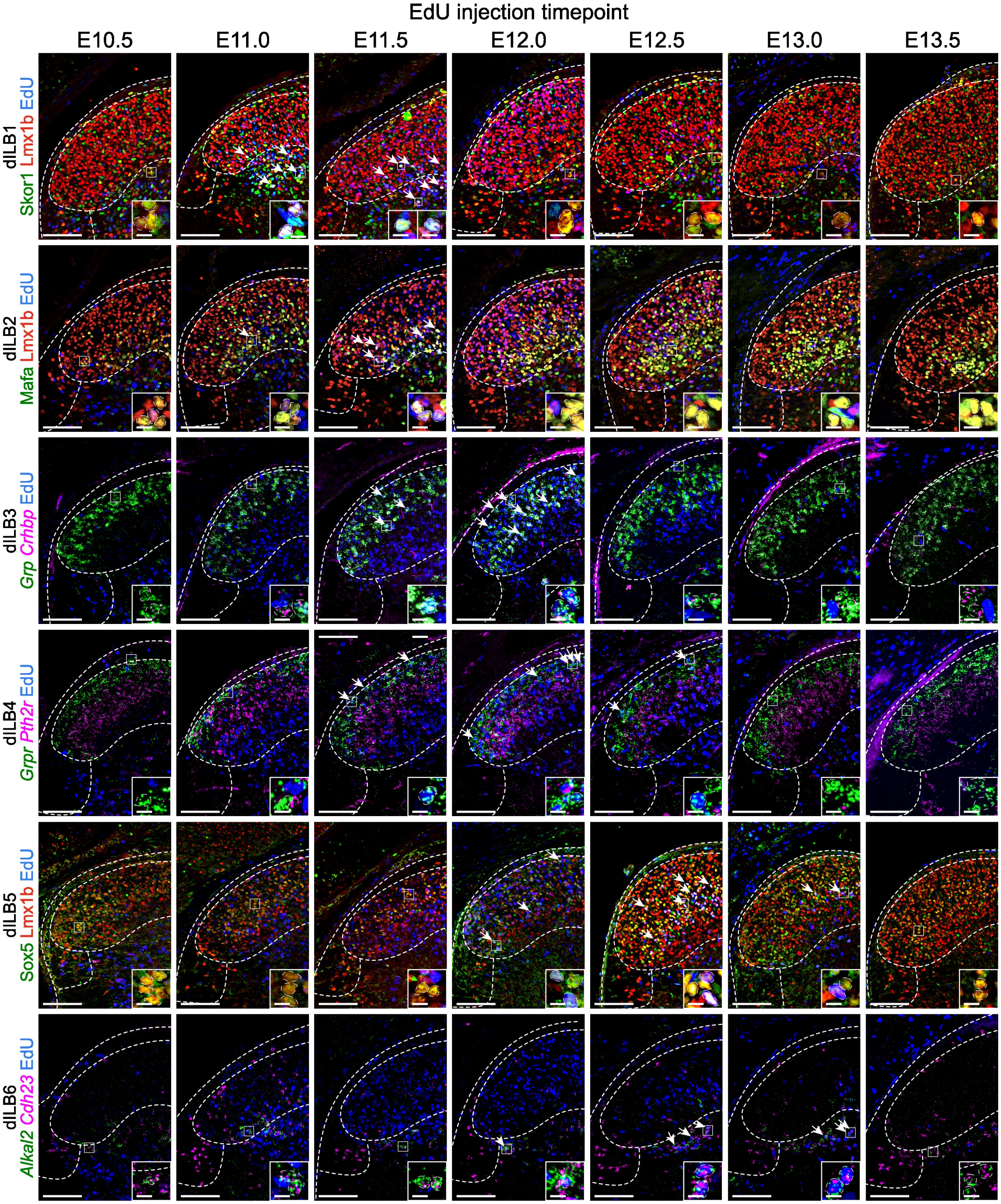

EdU injection timepoint
E10.5
E11.0
E11.5
E12.0
E12.5
E13.0
E13.5
dILB1 Skor1 Lmx1b EdU
dILB2 Mafa Lmx1b EdU
dILB3 Grp Crhbp EdU
dILB4 Grpr Pth2r EdU
dILB5 Sox5 Lmx1b EdU
dILB6 Alkal2 Cdh23 EdU

Fig. S11: EdU birthdating of dILB families.

Representative images of the lateral dorsal horn of the spinal cord from E16.5 embryos having received EdU pulses at either E10.5, E11.0, E11.5, E12.0, E12.5, E13.0 or E13.5 respectively. A subset of dILB families are identified by coincident expression of two genes (red or magenta and green) revealed by RNA Scope in-situ hybridization or nuclear protein expression; dILB1 – Lmx1b and Skor1, dILB2 – Lmx1b and Mafa, dILB3 – *Grp* and *Crhbp*, dILB4 – *Grpr* excluding *Pth2r*, dILB5 – Lmx1b and Sox5 and dILB6 – *Cdh23* and *Alkal2*. Arrows indicate dILB family neurons expressing the corresponding marker gene set and which are labeled with nuclear EdU (blue). Dashed lines indicate the border of the superficial dorsal horn with the deep dorsal horn and the lateral spinal nucleus, the lateral spinal nucleus with the lateral funiculus, and the pia. Insets indicate examples of triple-positive neurons surrounded by dashed lines. Example images from this figure used in Fig. 2G are reused here for contextual purposes.
Numbers: Individual litters of embryos from a mother given a single EdU injection were considered a single biological replicate. E10.5 (n=5 litters), E11.0 (n=4 litters), E11.5 (n=5 litters), E12.0 (n=5 litters), E12.5 (n=5 litters), E13.0 (n=5 litters), E13.5 (n=5 litters).
Scale bars: 250 µm, 25 µm for inset panels.
Abbrevations: EdU - 5-ethynyl-2'-deoxyuridine.

A

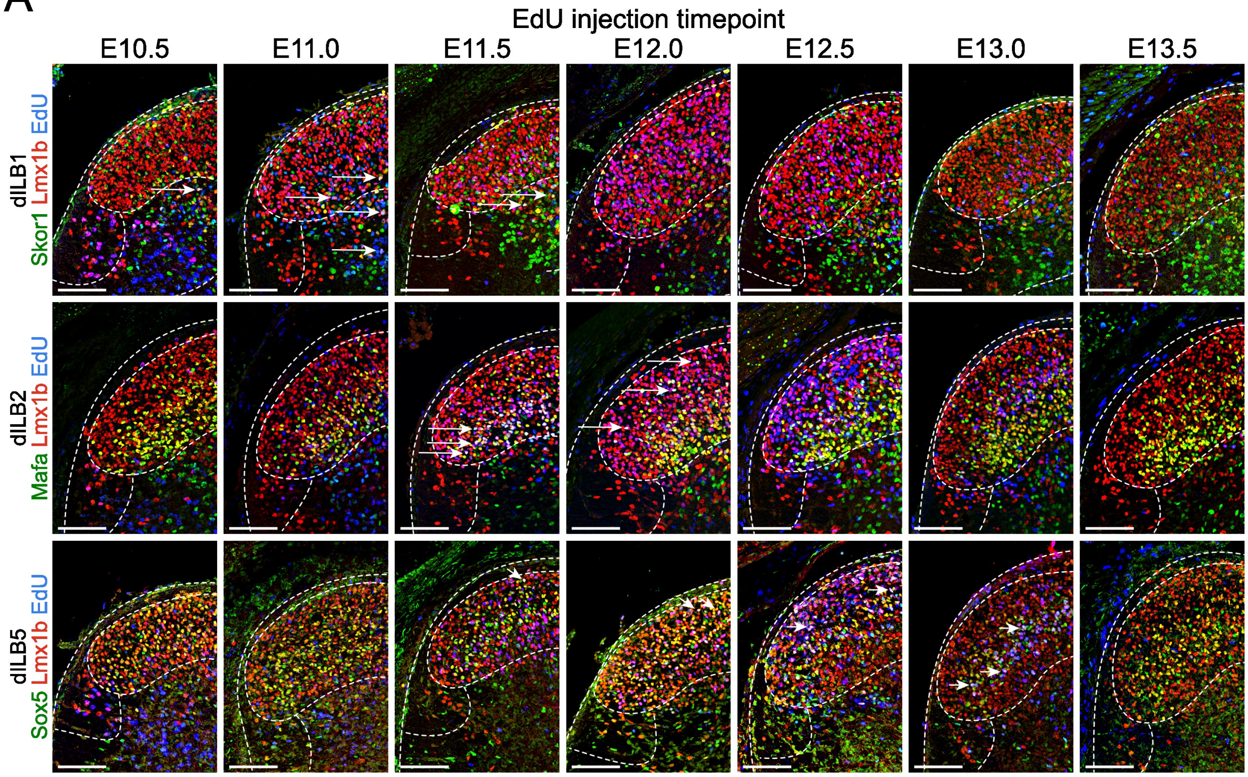

EdU injection timepoint
E10.5
E11.0
E11.5
E12.0
E12.5
E13.0
E13.5
dILB1 Skor1 Lmx1b EdU
dILB2 Mafa Lmx1b EdU
dILB5 Sox5 Lmx1b EdU

B

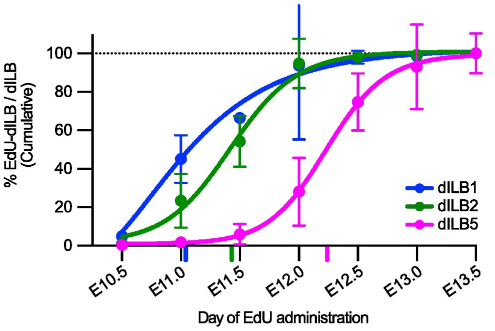

% EdU-dILB / dILB (Cumulative)
100
80
60
40
20
0
E10.5
E11.0
E11.5
E12.0
E12.5
E13.0
E13.5
Day of EdU administration
dILB1
dILB2
dILB5

Fig. S12: EdU birthdating of dILB neurons in a cohort unpaired with in-situ hybridization.

Embryos from birthdated litters where separate embryos were not appropriately fixed for in-situ hybridization were only examined for dILB families identifiable using immunohistochemistry (dILB1, dILB2 and dILB5). This experiment was supplanted by those in Fig. 2, S7 and S8, and thus not all litters were collected in triplicate prior to conducting subsequent experiments. Despite this, these experiments provide independent support the same birth order for dILB1, dILB2 and dILB5. (**A**) Representative images of the lateral dorsal horn of the spinal cord from E16.5 embryos having received EdU pulses at either E10.5, E11.0, E11.5, E12.0, E12.5, E13.0 or E13.5 respectively. A subset of dILB families are identified by coincident expression of two nuclear proteins (red and green) which exclude all other dIL neuron families; dILB1 – Lmx1b and Skor1, dILB2 – Lmx1b and Mafa and dILB5 – Lmx1b and Sox5. Arrows indicate dILB family neurons expressing the corresponding marker gene set and which are labeled with nuclear EdU (blue). Dashed lines indicate the border of the superficial dorsal horn with the deep dorsal horn and the lateral spinal nucleus, the lateral spinal nucleus with the lateral funiculus, and the pia. Insets indicate examples of triple-positive neurons surrounded by dashed lines.
(**B**) Plot showing the cumulative labeling of dILB1, dILB2 or dILB5 neurons by EdU over subsequent embryonic days. Points represent cumulative EdU labeled for each dILB family; individual data points are mean cumulative EdU labeling for all embryos of a single litter. Lines represent best-fit sigmoidal relationships of means of each dILB family.
Numbers: Images and cell counts shown here are derived from the same cohort of embryos analyzed in Fig. 1A, 1B, 3A and 3B; however, here data from all embryos in a single litter given a single EdU injection were averaged and considered a single biological replicate. E10.5 (n=2-3 litters), E11.0 (n=3 litters), E11.5 (n=4 litters), E12.0 (n=3 litters), E12.5 (n=4 litters), E13.0 (n=3 litters), E13.5 (n=4 litters).
Scale bars: 250 µm.
Abbrevations: EdU - 5-ethynyl-2'-deoxyuridine.

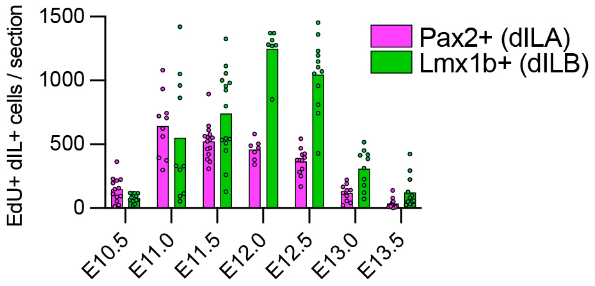
EdU+ dIL+ cells / section
1500
1000
500
0
E10.5
E11.0
E11.5
E12.0
E12.5
E13.0
E13.5
Pax2+ (dILA)
Lmx1b+ (dILB)

Figure S13: Neurogenesis rates of dILA and dILB neurons by embryonic day

Neuron counts per section of inhibitory (Pax2+, magenta) or excitatory (Lmx1b+, green) neurons that were EdU positive following EdU pulses at the indicated timepoints. Data points represent the mean double-positive neurons counted across all sections from a single embryo pulsed at the respective timepoint. Bars indicate mean double-positive neurons counted across all replicate embryos at each respective timepoint.

Numbers: n=79 embryos, 7-15 per timepoint. The same cohort of embryos is represented in Figure 1A and Figure 3A-C.

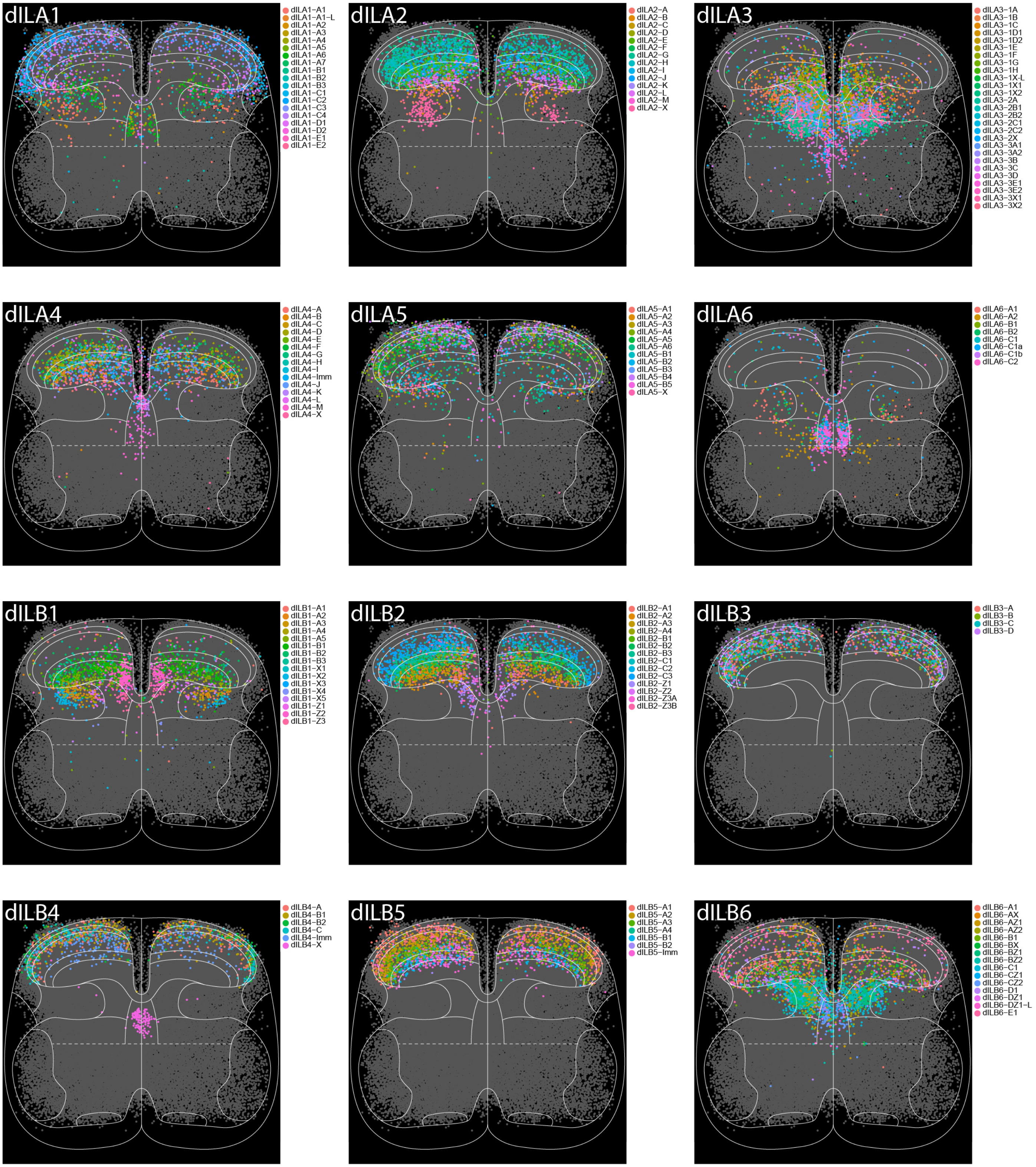
dILA1
dILA1−A1
dILA1−A1−L
dILA1−A2
dILA1−A3
dILA1−A4
dILA1−A5
dILA1−A6
dILA1−A7
dILA1−B1
dILA1−B2
dILA1−B3
dILA1−C1
dILA1−C2
dILA1−C3
dILA1−C4
dILA1−D1
dILA1−D2
dILA1−E1
dILA1−E2
dILA2
dILA2−A
dILA2−B
dILA2−C
dILA2−D
dILA2−E
dILA2−F
dILA2−G
dILA2−H
dILA2−I
dILA2−J
dILA2−K
dILA2−L
dILA2−M
dILA2−X
dILA3
dILA3−1A
dILA3−1B
dILA3−1C
dILA3−1D1
dILA3−1D2
dILA3−1E
dILA3−1F
dILA3−1G
dILA3−1H
dILA3−1X−L
dILA3−1X1
dILA3−1X2
dILA3−2A
dILA3−2B1
dILA3−2B2
dILA3−2C1
dILA3−2C2
dILA3−2X
dILA3−3A1
dILA3−3A2
dILA3−3B
dILA3−3C
dILA3−3D
dILA3−3E1
dILA3−3E2
dILA3−3X1
dILA3−3X2
dILA4
dILA4−A
dILA4−B
dILA4−C
dILA4−D
dILA4−E
dILA4−F
dILA4−G
dILA4−H
dILA4−I
dILA4−Imm
dILA4−J
dILA4−K
dILA4−L
dILA4−M
dILA4−X
dILA5
dILA5−A1
dILA5−A2
dILA5−A3
dILA5−A4
dILA5−A5
dILA5−A6
dILA5−B1
dILA5−B2
dILA5−B3
dILA5−B4
dILA5−B5
dILA5−X
dILA6
dILA6−A1
dILA6−A2
dILA6−B1
dILA6−B2
dILA6−C1
dILA6−C1a
dILA6−C1b
dILA6−C2
dILB1
dILB1−A1
dILB1−A2
dILB1−A3
dILB1−A4
dILB1−A5
dILB1−B1
dILB1−B2
dILB1−B3
dILB1−X1
dILB1−X2
dILB1−X3
dILB1−X4
dILB1−X5
dILB1−Z1
dILB1−Z2
dILB1−Z3
dILB2
dILB2−A1
dILB2−A2
dILB2−A3
dILB2−A4
dILB2−B1
dILB2−B2
dILB2−B3
dILB2−C1
dILB2−C2
dILB2−C3
dILB2−Z1
dILB2−Z2
dILB2−Z3A
dILB2−Z3B
dILB3
dILB3−A
dILB3−B
dILB3−C
dILB3−D
dILB4
dILB4−A
dILB4−B1
dILB4−B2
dILB4−C
dILB4−Imm
dILB4−X
dILB5
dILB5−A1
dILB5−A2
dILB5−A3
dILB5−A4
dILB5−B1
dILB5−B2
dILB5−Imm
dILB6
dILB6−A1
dILB6−AX
dILB6−AZ1
dILB6−AZ2
dILB6−B1
dILB6−BX
dILB6−BZ1
dILB6−BZ2
dILB6−C1
dILB6−CZ1
dILB6−CZ2
dILB6−D1
dILB6−DZ1
dILB6−DZ1−L
dILB6−E1

Fig. S14: Spatial transcriptomics reveals laminar position of dIL family subtypes.

Scatter plots depict the coordinates of all wild-type brachial (C5-C8) E16.5 neurons examined in the Xenium spatial transcriptomics assay. Cell-type identity was predicted using RCTD, with single-cell RNA-sequencing data from Fig. 1B as a reference. Cell coordinates were normalized to anatomic landmarks from each profiled spinal cord section; subsequently, the plane of cell coordinates was rotated and stretched to fit an ideal diagram of a spinal cord (white solid lines in each panel, with approximate laminar boundaries in dashed white lines). This process was identical for all cells regardless of annotation. Cells assigned to each of 6 dILA and 6 dILB families are plotted separately, as indicated in the top left of each panel. Cells not belonging to the indicated dIL family are plotted in gray; otherwise, they are colored based on their family subtype identity in the corresponding legend to each panel.
Numbers: Cell coordinates were derived from six whole sections and seven hemisections (N=9.5 sections) among n=3 replicate wild-type E16.5 spinal cords. From these sections 54,166 cells were identified as neurons and plotted, of which 27,714 were annotated as dIL neurons. The same data are presented in Fig. 3D with dIL family annotations.
Abbreviations: RCTD – Robust cell-type decomposition (R library).

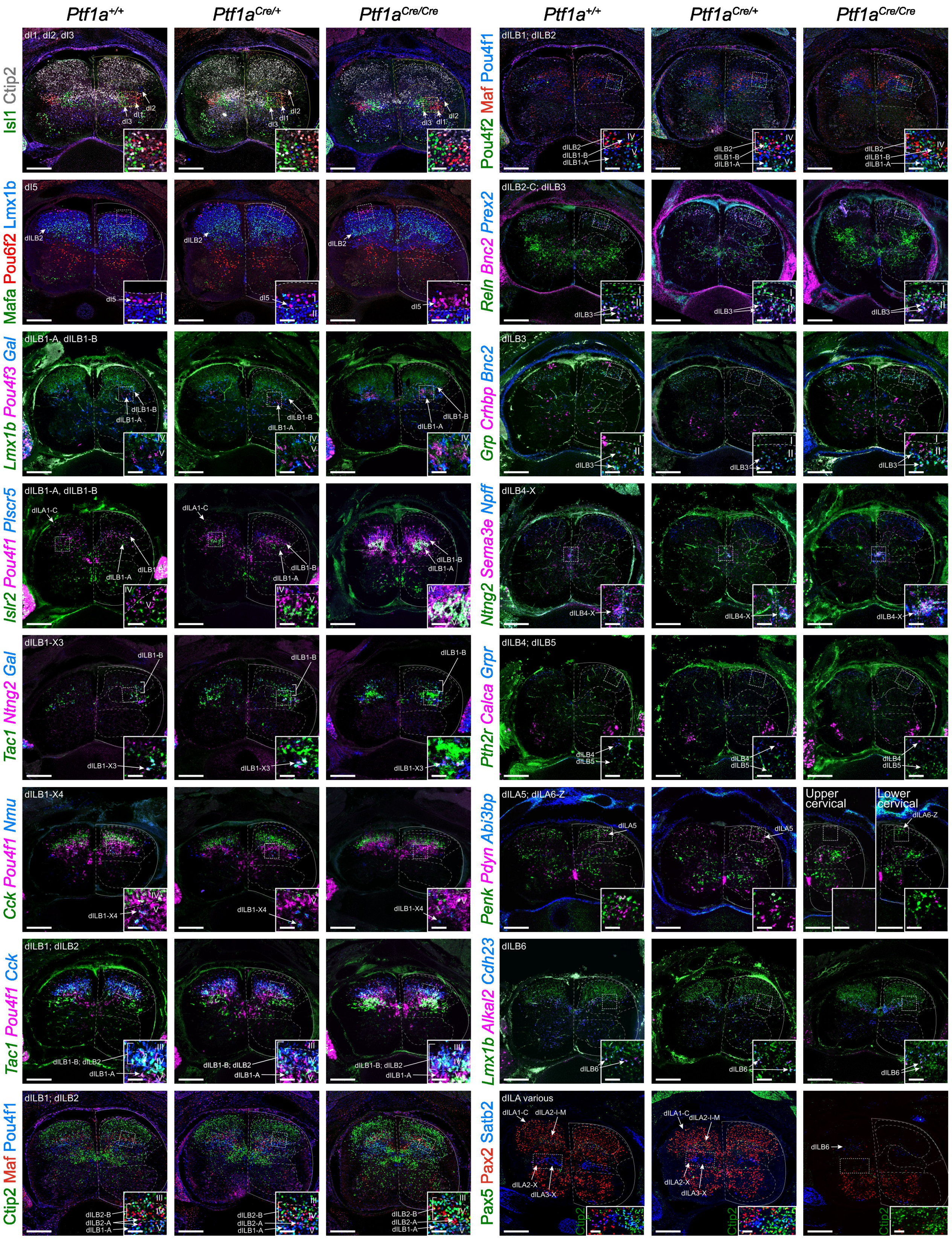

Ptf1a+/+
Ptf1aCre/+
Ptf1aCre/Cre
Ptf1a+/+
Ptf1aCre/+
Ptf1aCre/Cre
Foxd3 Barhl1 Isl1 Ctip2
dI1, dI2, dI3
Mafa Pou6f2 Lmx1b
dI5
dILB2
Lmx1b Pou4f3 Gal
dILB1-A, dILB1-B
Islr2 Pou4f1 Plscr5
dILB1-A, dILB1-B
dILA1-C
Tac1 Ntng2 Gal
dILB1-X3
Cck Pou4f1 Nmu
dILB1-X4
Tac1 Pou4f1 Cck
dILB1; dILB2
dILB1-B; dILB2
dILB1-A
Ctip2 Maf Pou4f1
dILB1; dILB2
dILB2-B
dILB2-A
dILB1-A
Pou4f2 Maf Pou4f1
dILB1; dILB2
Reln Bnc2 Prex2
dILB2-C; dILB3
Grp Crhbp Bnc2
dILB3
Ntng2 Sema3e Npff
dILB4-X
Pth2r Calca Grpr
dILB4; dILB5
Penk Pdyn Abi3bp
dILA5; dILA6-Z
Upper cervical
Lower cervical
Lmx1b Alkal2 Cdh23
dILB6
Pax5 Pax2 Satb2
dILA various
dILA1-C
dILA2-I-M
dILA2-X
dILA3-X
Ctip2

Fig. S15: dILB neurons laminate normally in absence of dILA neurons – images.

Images of immunohistochemistry and RNA Scope in-situ hybridization of various cardinal excitatory, dILB dorsal horn neuron types and selected dILA populations (indicated in the top left of each row) are examined in E16.5 spinal cords from embryos wild-type, heterozygous-null, or null for *Ptf1a*. dILA neurons are largely absent from null spinal cords while excitatory neurons are anatomically similar to controls (though present in increased numbers). White arrows indicate examples of dIL family subtypes. Insets at the bottom right of each panel are magnified fields of view from boxes marked by dashed white lines in the larger panel. Other solid and dashed white lines indicate approximate anatomical boundaries of the spinal cord. Images of *Plscr5* (row 4, left) are reused here from Fig. 4, for contextual purposes.
Numbers: Representative images from n=4 $Ptf1a^{+/+}$; n=4 $Ptf1a^{Cre/+}$; n=4 $Ptf1a^{Cre/Cre}$ E16.5 spinal cords.
Scale bars: 250 µm, 50 µm for inset panels.

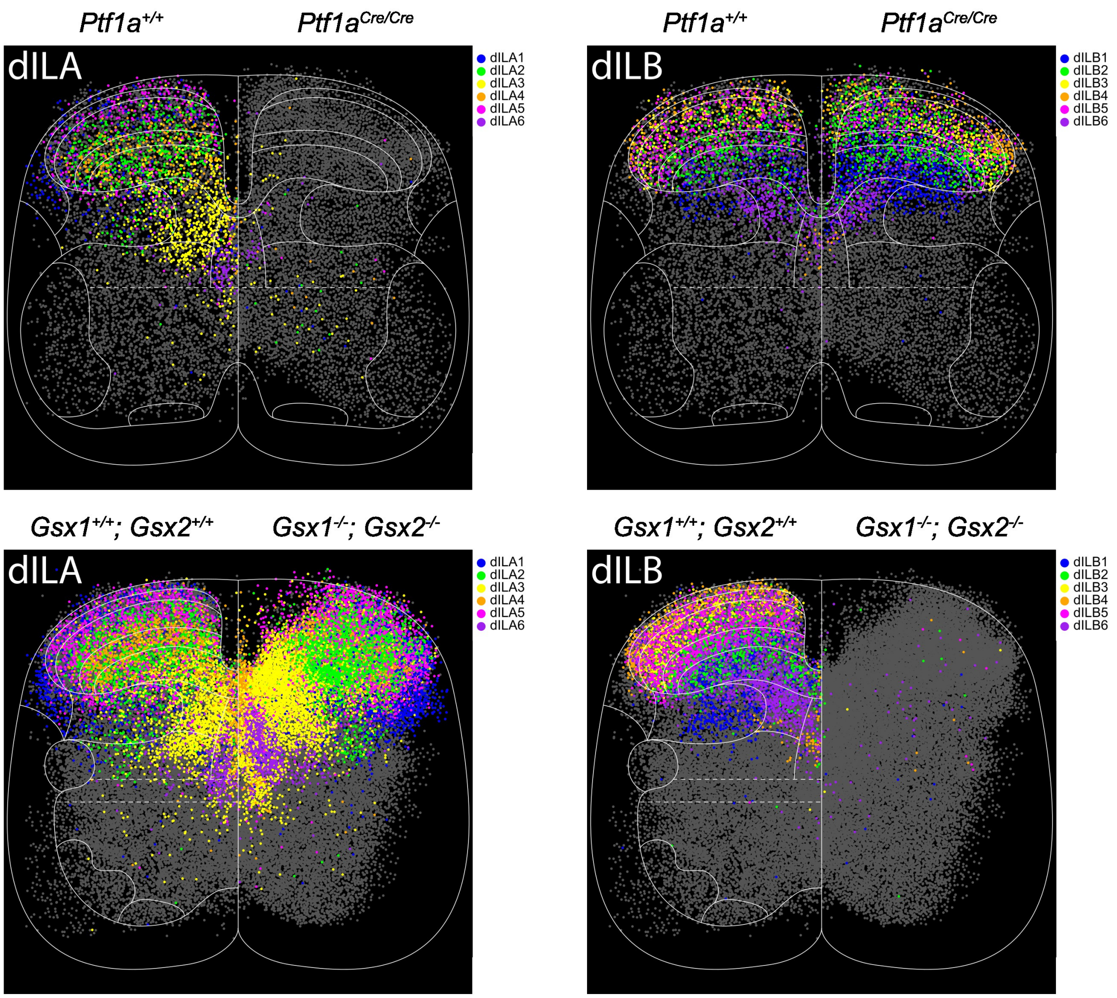

Ptf1a+/+
Ptf1aCre/Cre
dILA
dILA1
dILA2
dILA3
dILA4
dILA5
dILA6
Ptf1a+/+
Ptf1aCre/Cre
dILB
dILB1
dILB2
dILB3
dILB4
dILB5
dILB6
Gsx1+/+; Gsx2+/+
Gsx1-/-; Gsx2-/-
dILA
dILA1
dILA2
dILA3
dILA4
dILA5
dILA6
Gsx1+/+; Gsx2+/+
Gsx1-/-; Gsx2-/-
dILB
dILB1
dILB2
dILB3
dILB4
dILB5
dILB6

Fig. S16: Trans-differentiation of dIL neurons in Ptf1a and Gsx1/2 null embryos.

Scatter plots depict the coordinates of wild-type, *Ptf1a*-null or *Gsx1/2*-null E16.5 neurons examined in the Xenium spatial transcriptomics assay, colored by either dILA family (column 1) or dILB family (column 2). Here, segmentally matched wild-type cell coordinates have been mirrored and overlaid on the left side of each diagram and segmentally corresponding cell coordinates from either *Ptf1a*-null tissue (row 1) or *Gsx1/2*-null tissue (row 2) have been mirrored and overlaid on the right side of each diagram in order to facilitate anatomical comparisons between genotype. *Ptf1a*-null cells have been compared to brachial wild-type cell coordinates displayed in Fig. S14 while *Gsx1/2*-null tissue analyzed from upper thoracic segments have been compared to wild-type thoracic cell coordinates not previously displayed. Wild-type neurons have been evenly downsampled from each neuron subtype annotation to match the total number of null cells displayed per diagram. Notably, dILA neurons are absent from Ptf1a-null tissue and dILB neurons are absent from *Gsx1/2*-null tissue. Cells not belonging to the indicated dIL family are plotted in gray. Solid and dashed white lines indicate approximate anatomical boundaries of the spinal cord.

Numbers: Spatial transcriptomics from E16.5 wild-type embryo brachial segments was previously described (Fig. S14). For E16.5 wild-type thoracic segments, cell coordinates were derived from five whole sections and one hemisection (N=5.5 sections), among n=3 replicate spinal cords. From these sections 27,758 cells were identified as neurons and plotted, of which 13,724 were annotated as dIL neurons. For E16.5 *Ptf1a*-null brachial segments, cell coordinates were derived from two whole sections (N=2 sections) among n=1 spinal cord. From these sections, 8,267 cells were identified as neurons, and of which 2,767 were dIL neurons. E16.5 brachial data was downsampled to 8,106 neurons of which 4,151 were dIL neurons to match. For E16.5 *Gsx1/2*-null thoracic segments, cell coordinates were derived from four whole sections and three hemisections (N=5.5 sections) among n=3 replicate spinal cords. From these sections, 24,737 cells were identified as neurons, and of which 12,217 were dIL neurons. E16.5 thoracic data was downsampled to 24,583 neurons with, also, 12,217 dIL neurons, to match.

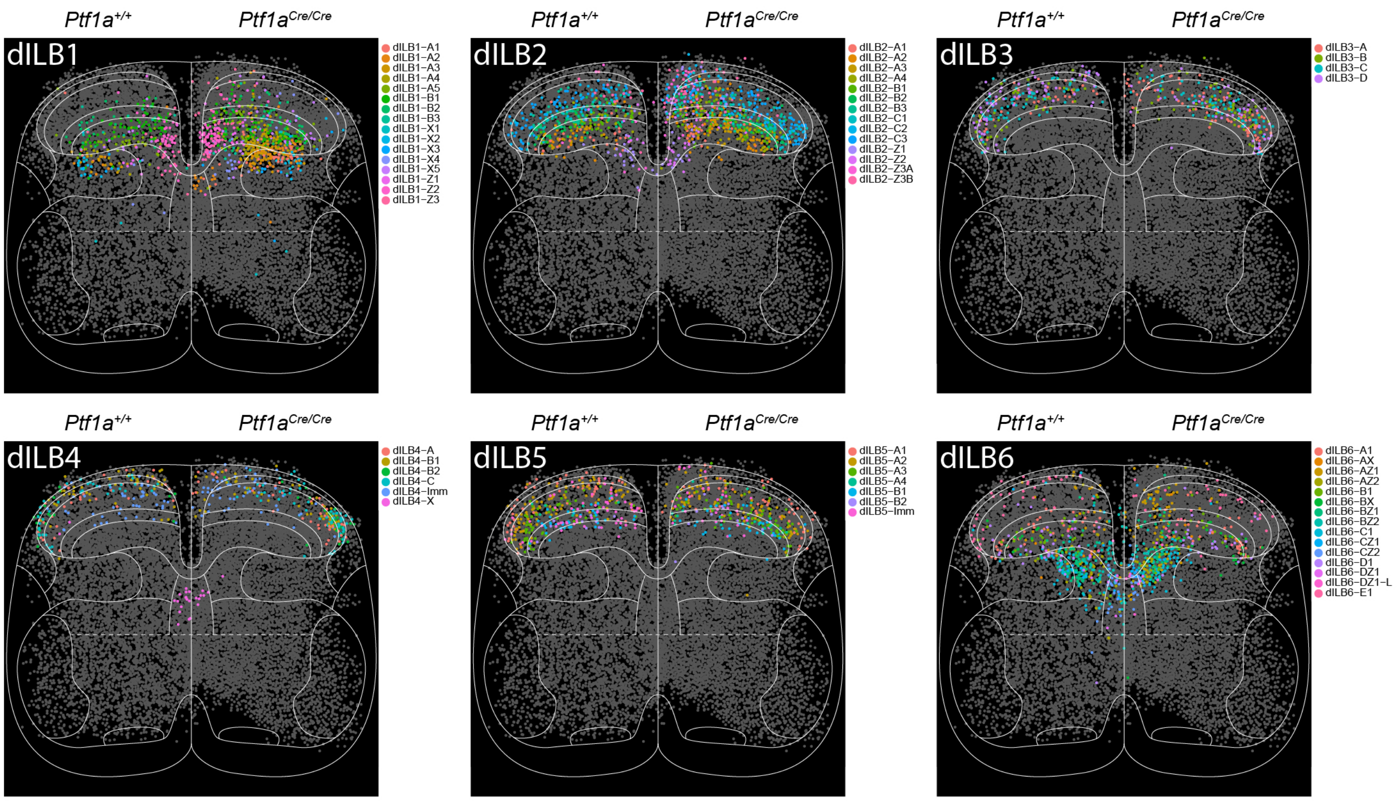
Ptf1a+/+
Ptf1aCre/Cre
dILB1
dILB1–A1
dILB1–A2
dILB1–A3
dILB1–A4
dILB1–A5
dILB1–B1
dILB1–B2
dILB1–B3
dILB1–X1
dILB1–X2
dILB1–X3
dILB1–X4
dILB1–X5
dILB1–Z1
dILB1–Z2
dILB1–Z3
dILB2
dILB2–A1
dILB2–A2
dILB2–A3
dILB2–A4
dILB2–B1
dILB2–B2
dILB2–B3
dILB2–C1
dILB2–C2
dILB2–C3
dILB2–Z1
dILB2–Z2
dILB2–Z3A
dILB2–Z3B
dILB3
dILB3–A
dILB3–B
dILB3–C
dILB3–D
dILB4
dILB4–A
dILB4–B1
dILB4–B2
dILB4–C
dILB4–Imm
dILB4–X
dILB5
dILB5–A1
dILB5–A2
dILB5–A3
dILB5–A4
dILB5–B1
dILB5–B2
dILB5–Imm
dILB6
dILB6–A1
dILB6–AX
dILB6–AZ1
dILB6–AZ2
dILB6–B1
dILB6–BX
dILB6–BZ1
dILB6–BZ2
dILB6–C1
dILB6–CZ1
dILB6–CZ2
dILB6–D1
dILB6–DZ1
dILB6–DZ1–L
dILB6–E1

Fig. S17: dILB neurons laminate normally in absence of dILA neurons – Xenium.

Scatter plots depict the coordinates of wild-type and *Ptf1a*-null E16.5 neurons examined in the Xenium spatial transcriptomics assay, sorted into each of six dILB families and colored by subtype annotation. As described previously, wild-type cells are displayed on the left and null cells on the right of each diagram, and wild-type cells were downsampled to match the number of null cells. All cells presented here are also presented in Fig. 4 and Fig. S16 with family annotations. Cells not belonging to the indicated dIL family are plotted in gray. Solid and dashed white lines indicate approximate anatomical boundaries of the spinal cord.

Figure S18: dILA neurons lose laminar patterning in absence of dILB neurons – images

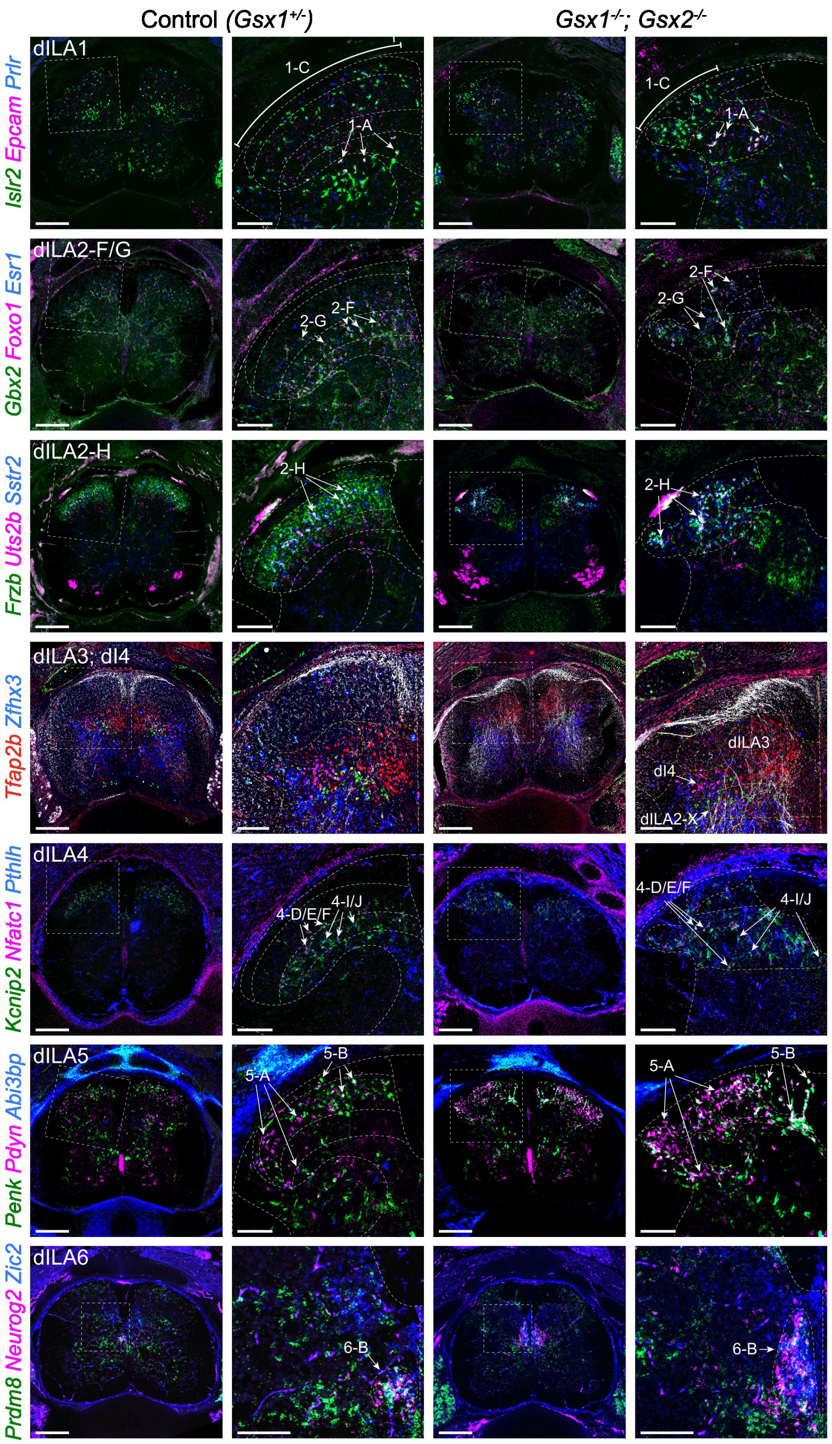

Fig. S18: dILA neurons lose laminar patterning in absence of dILB neurons – images.

Images of immunohistochemistry and RNA Scope in-situ hybridization of dILA family subtypes (indicated in the top left of each row) are examined in E16.5 spinal cords from embryos heterozygous-null for *Gsx1* (as control), or null for both *Gsx1* and *Gsx2*. dILA neurons normally present in laminae are no longer present in laminae and are largely disorganized. Unlaminated dILA neurons in the deep dorsal horn are slightly displaced, though in anatomically similar locations. dILB neurons are largely absent from null spinal cords. White arrows indicate examples of dIL family subtypes. For each genotype, the left column of images depicts an entire spinal cord section while the right column depicts a magnified image from the dashed white line box in the left column. Other solid and dashed white lines indicate approximate anatomical boundaries of the spinal cord.
Numbers: Representative images from n=4 $Gsx1^{+/-}$; $Gsx2^{+/+}$, n=4 $Gsx1^{-/-}$; $Gsx2^{-/-}$.
Scale bars: 250 µm for left column, 100 µm for magnified panels (right column) for each genotype.
Images of PV (row 4) are reused here from Fig. 4, for contextual purposes.

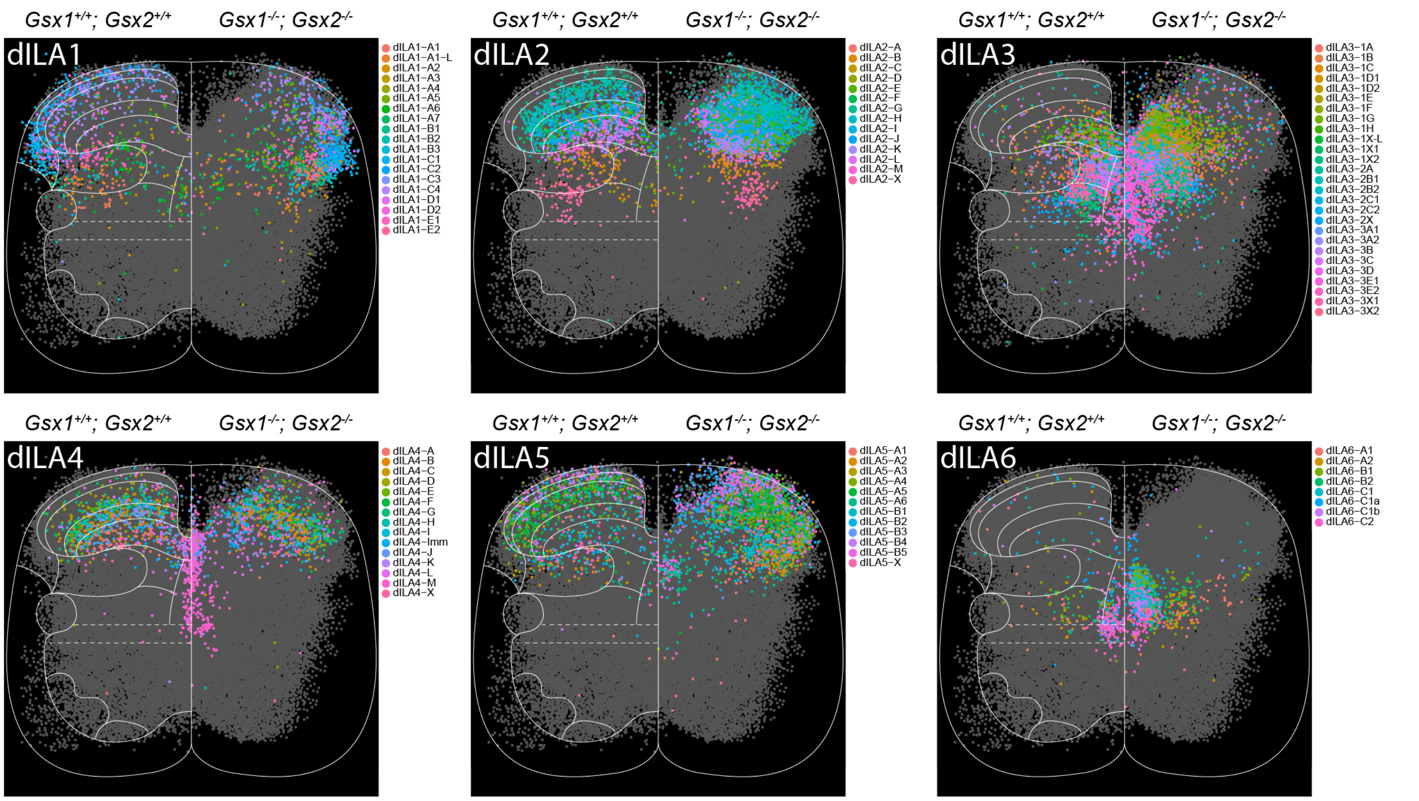
Gsx1+/+; Gsx2+/+
Gsx1-/-; Gsx2-/-
dILA1
dILA1−A1
dILA1−A1−L
dILA1−A2
dILA1−A3
dILA1−A4
dILA1−A5
dILA1−A6
dILA1−A7
dILA1−B1
dILA1−B2
dILA1−B3
dILA1−C1
dILA1−C2
dILA1−C3
dILA1−C4
dILA1−D1
dILA1−D2
dILA1−E1
dILA1−E2
Gsx1+/+; Gsx2+/+
Gsx1-/-; Gsx2-/-
dILA2
dILA2−A
dILA2−B
dILA2−C
dILA2−D
dILA2−E
dILA2−F
dILA2−G
dILA2−H
dILA2−I
dILA2−J
dILA2−K
dILA2−L
dILA2−M
dILA2−X
Gsx1+/+; Gsx2+/+
Gsx1-/-; Gsx2-/-
dILA3
dILA3−1A
dILA3−1B
dILA3−1C
dILA3−1D1
dILA3−1D2
dILA3−1E
dILA3−1F
dILA3−1G
dILA3−1H
dILA3−1X-L
dILA3−1X1
dILA3−1X2
dILA3−2A
dILA3−2B1
dILA3−2B2
dILA3−2C1
dILA3−2C2
dILA3−2X
dILA3−3A1
dILA3−3A2
dILA3−3B
dILA3−3C
dILA3−3D
dILA3−3E1
dILA3−3E2
dILA3−3X1
dILA3−3X2
Gsx1+/+; Gsx2+/+
Gsx1-/-; Gsx2-/-
dILA4
dILA4−A
dILA4−B
dILA4−C
dILA4−D
dILA4−E
dILA4−F
dILA4−G
dILA4−H
dILA4−I
dILA4−Imm
dILA4−J
dILA4−K
dILA4−L
dILA4−M
dILA4−X
Gsx1+/+; Gsx2+/+
Gsx1-/-; Gsx2-/-
dILA5
dILA5−A1
dILA5−A2
dILA5−A3
dILA5−A4
dILA5−A5
dILA5−A6
dILA5−B1
dILA5−B2
dILA5−B3
dILA5−B4
dILA5−B5
dILA5−X
Gsx1+/+; Gsx2+/+
Gsx1-/-; Gsx2-/-
dILA6
dILA6−A1
dILA6−A2
dILA6−B1
dILA6−B2
dILA6−C1
dILA6−C1a
dILA6−C1b
dILA6−C2

Fig. S19: dILA neurons lose laminar patterning in absence of dILB neurons – Xenium.

Scatter plots depict the coordinates of wild-type and *Gsx1/2*-null E16.5 neurons examined in the Xenium spatial transcriptomics assay, sorted into each of six dILA families, and colored by subtype annotation. As described previously, wild-type cells are displayed on the left and null cells on the right of each diagram, and wild-type cells were downsampled to match the number of null cells. All cells presented here are also presented with family-level annotations in Fig. S16 and with selected refined annotations in Fig. 4. Cells not belonging to the indicated dIL family are plotted in gray. Solid and dashed white lines indicate approximate anatomical boundaries of the spinal cord.

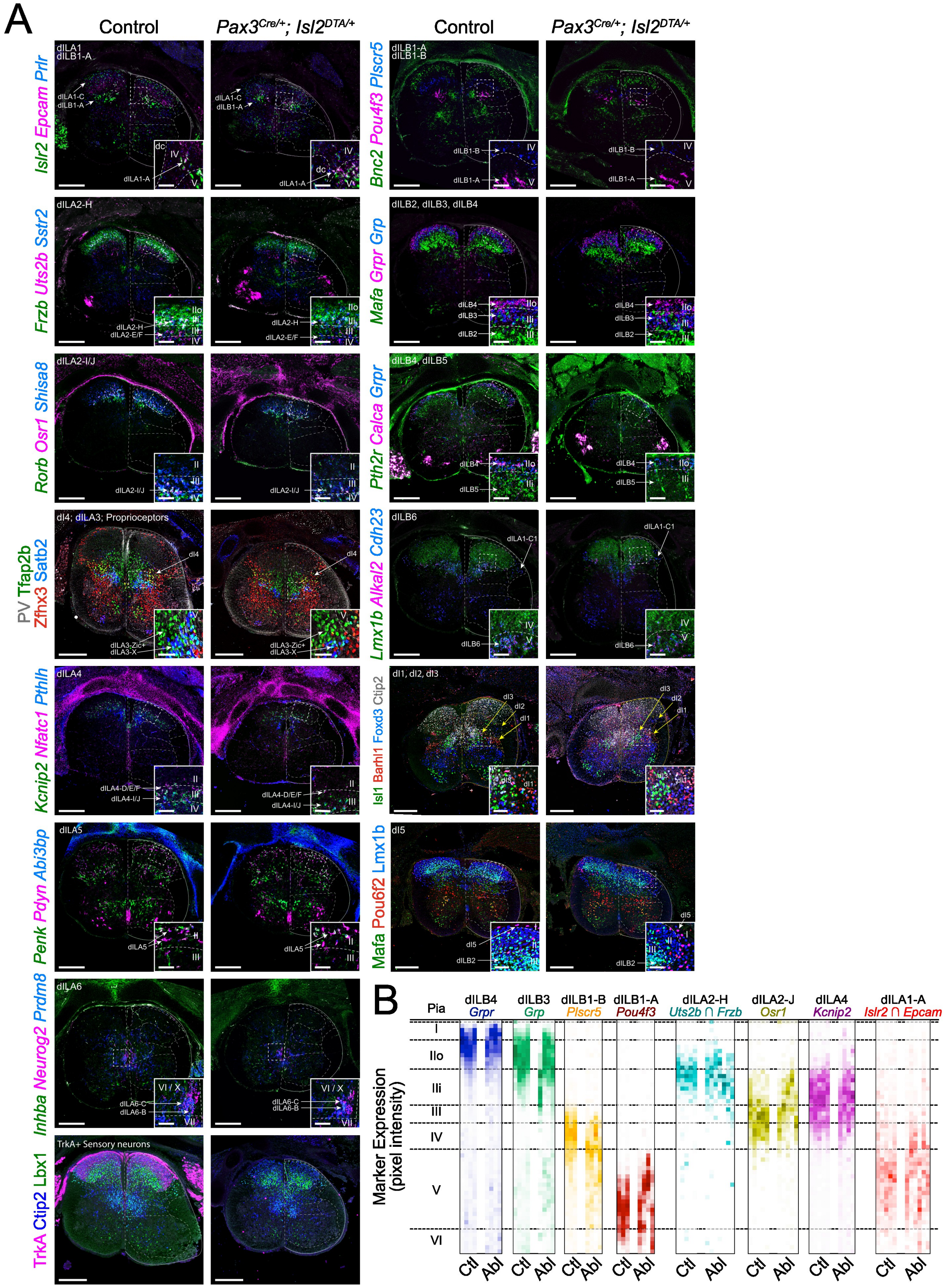
A
Control
Pax3Cre/+; Isl2DTA/+
Control
Pax3Cre/+; Isl2DTA/+
Islr2 Epcam Prlr
dILA1
dILB1-A
Bnc2 Pou4f3 Plscr5
dILB1-A
dILB1-B
Frzb Uts2b Sstr2
dILA2-H
Mafa Grpr Grp
dILB2, dILB3, dILB4
Rorb Osr1 Shisa8
dILA2-I/J
Pth2r Calca Grpr
dILB4, dILB5
PV Tfap2b Zfhx3 Satb2
dI4; dILA3; Proprioceptors
Lmx1b Alkal2 Cdh23
dILB6
Kcnip2 Nfatc1 Pthln
dILA4
Isl1 Barhl1 Foxd3 Ctip2
dI1, dI2, dI3
Penk Pdyn Abi3bp
dILA5
Mafa Pou6f2 Lmx1b
dI5
Inhba Neurog2 Prdm8
dILA6
TrkA Ctip2 Lbx1
TrkA+ Sensory neurons
B
Pia
I
IIo
IIi
III
IV
V
VI
Marker Expression (pixel intensity)
dILB4 Grpr
dILB3 Grp
dILB1-B Plscr5
dILB1-A Pou4f3
dILA2-H Uts2b ∩ Frzb
dILA2-J Osr1
dILA4 Kcnip2
dILA1-A Islr2 ∩ Epcam
Ctl
Abl

Fig. S20: Sensory neuron afferents are dispensable for dorsal horn structure.

(**A**) Immunohistochemistry and RNA Scope in-situ hybridization of dILA family subtypes, dILB family subtypes, and sensory afferents (indicated in the top left of each row) are examined in E16.5 spinal cords from control littermates, (either $Pax3^{Cre/+}$ or $Isl2^{DTA/+}$) or embryos which are both $Pax3^{Cre/+}$ and $Isl2^{DTA/+}$, resulting in pan-sensory neuron ablation. DILA and dILB neurons laminate in sensory neuron-ablated spinal cord similarly to control spinal cords. Notable phenotypes are atrophy of the dorsal columns (due to a lack of sensory neuron axons), and mild atrophy of dorsal horn interneurons non-specifically when sensory neurons are absent. White arrows indicate examples of dIL family subtypes. Insets at the bottom right of each panel are magnified fields of view from boxes marked by dashed white lines in the larger panel. Other solid and dashed white lines indicate approximate anatomical boundaries of the spinal cord.
(**B**) Spatial distribution of individual gene or protein markers or marker sets (as indicated at the top of each box), showing the normalized proportion of total signal per dorso-ventral bin (color intensity) where the pia is set as the dorsal boundary and approximate laminar positions are shown. Biological replicates are grouped together as individual columns for either Ctl (control – either $Pax3^{Cre/+}$ or $Isl2^{DTA/+}$) or Abl (Ablated - $Pax3^{Cre/+}$; $Isl2^{DTA/+}$).
Numbers: Representative images and data from n=3-5 control (either $Pax3^{Cre/+}$ or $Isl2^{DTA/+}$), n=4-7 $Pax3^{Cre/+}$;$Isl2^{DTA/+}$ E16.5 spinal cords.
Statistics: See Table S12.
Scale bars: 250 µm, 50 µm for inset panels.

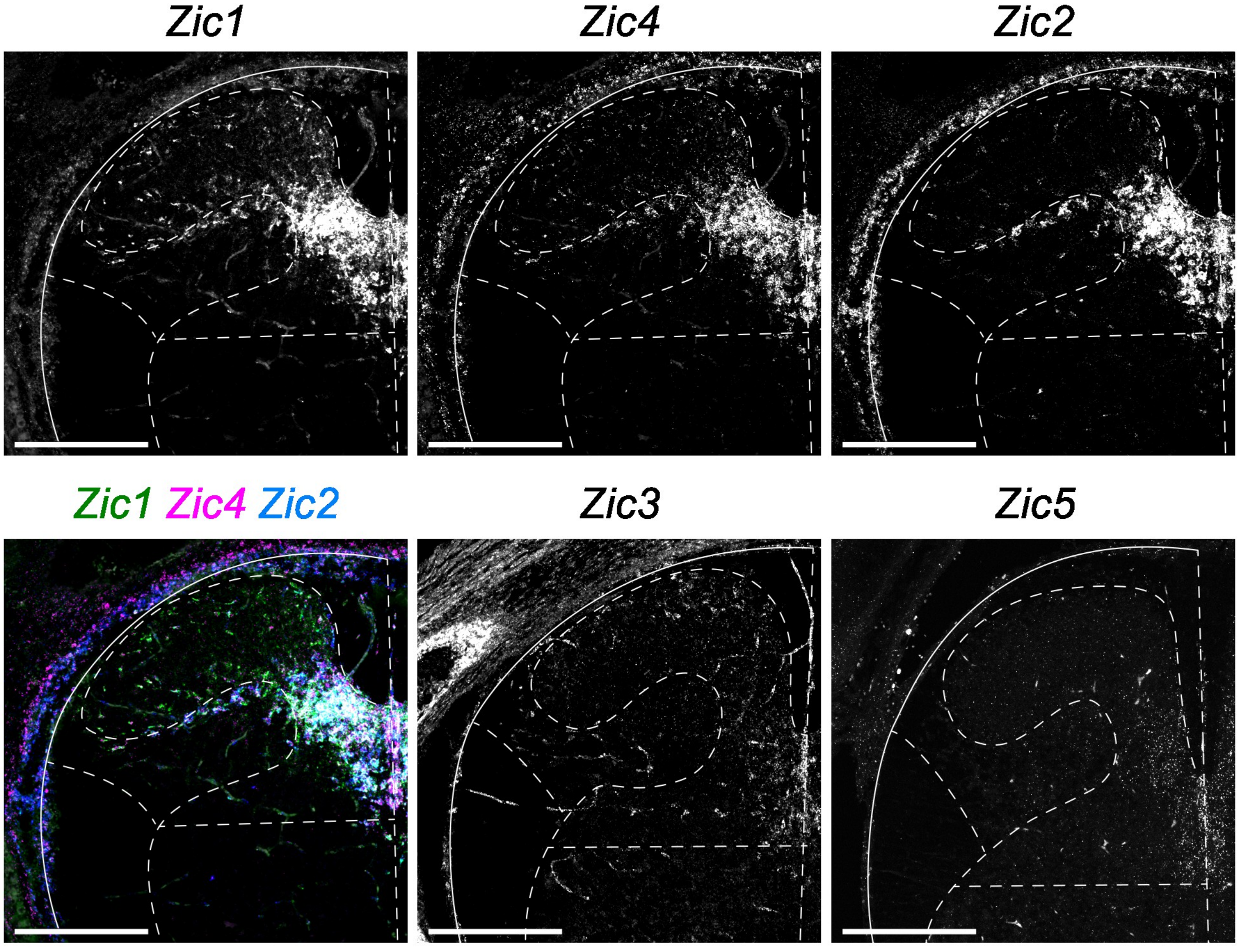
Zic1
Zic4
Zic2
Zic1 Zic4 Zic2
Zic3
Zic5

Fig. S21: Expression of Zic family genes in the dorsal horn.

Zic1-5 genes are expressed in stereotypical patterns restricted to dorsal horn neurons, with notable expression also along vasculature and meninges. The breadth and intensity of Zic gene expression corresponds well to that observed within single-cell RNA-sequencing of dIL neurons.
Numbers: Representative images from n=3 E16.5 wild-type mouse spinal cords
Scale bars: 250 µm

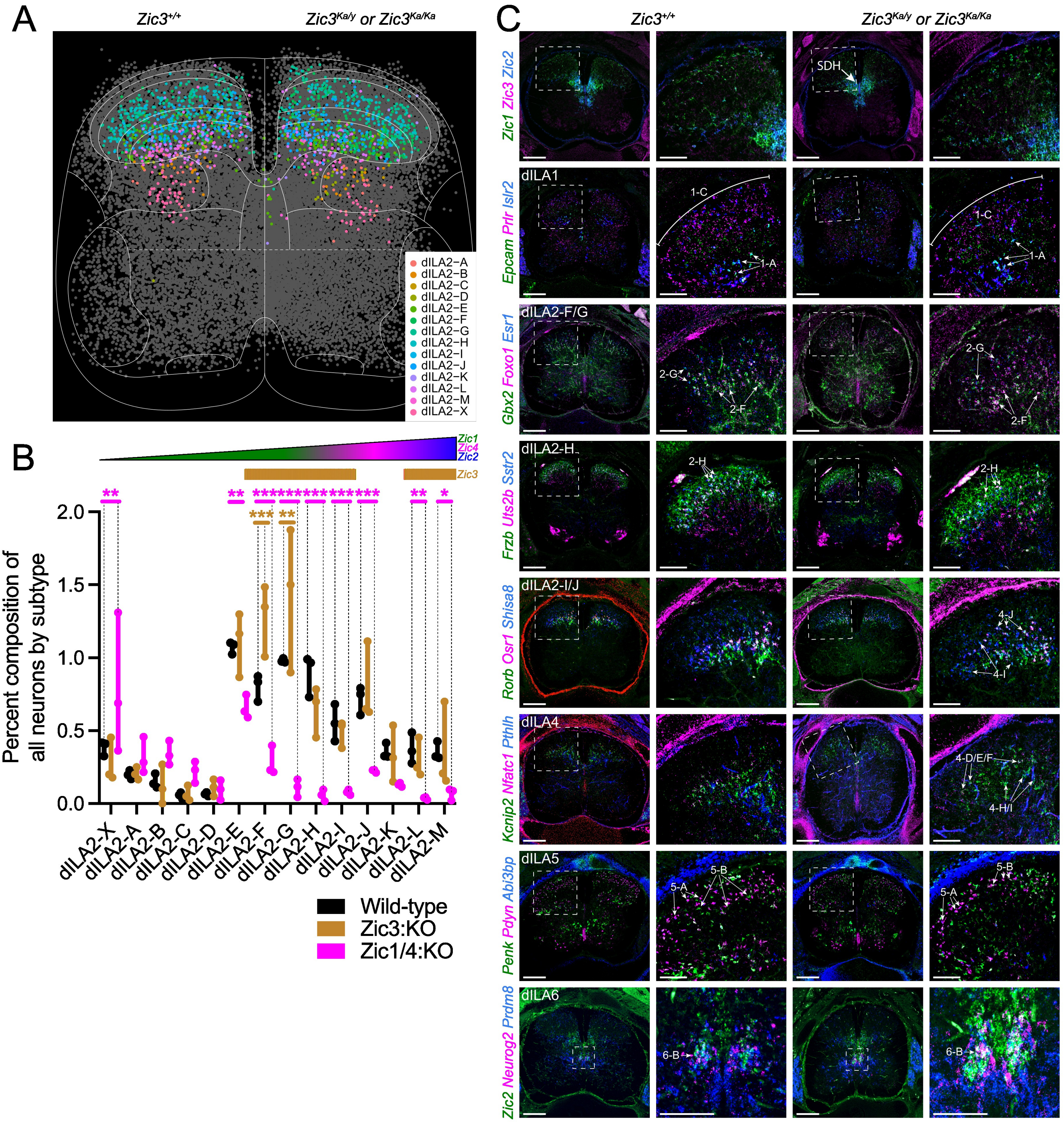
A
Zic3+/+
Zic3Ka/y or Zic3Ka/Ka
dILA2-A
dILA2-B
dILA2-C
dILA2-D
dILA2-E
dILA2-F
dILA2-G
dILA2-H
dILA2-I
dILA2-J
dILA2-K
dILA2-L
dILA2-M
dILA2-X
B
Zic1
Zic4
Zic2
Zic3
Percent composition of all neurons by subtype
Wild-type
Zic3:KO
Zic1/4:KO
C
Zic1 Zic3 Zic2
SDH
dILA1
Epcam Prlr Islr2
1-C
1-A
dILA2-F/G
Gbx2 Foxo1 Esr1
2-G
2-F
dILA2-H
Frzb Uts2b Sstr2
2-H
dILA2-I/J
Rorb Osr1 Shisa8
4-J
4-I
dILA4
Kcnip2 Nfatc1 Pthlh
4-D/E/F
4-H/I
dILA5
Penk Pdyn Abi3bp
5-A
5-B
dILA6
Zic2 Neurog2 Prdm8
6-B

Fig. S22: *Zic3* mildly represses formation of mid Zic-gradient subtypes of dIL neurons.

(**A**) The scatter plot depicts the coordinates of brachial wild-type and *Zic3*-null E16.5 neurons examined in the Xenium spatial transcriptomics assay and colored by subtype annotation of the dILA2 family (as an example). As described previously, wild-type cells are displayed on the left and null cells on the right of each diagram, and wild-type cells were downsampled to match the number of null cells. Cells not belonging to the dILA2 family are plotted in gray. Solid and dashed white lines indicate approximate anatomical boundaries of the spinal cord. (**B**) The fraction of dILA2 family subtypes of all neurons in *Zic3*-null spinal cords is depicted here compared to wild-type and *Zic1/4*-null data, gathered from predicted neuron subtype identities in Xenium spatial transcriptomics analyses. Wild-type and *Zic1/4*-null data are depicted in Fig. 5K and are displayed here again for reference. Vertical black dashed lines connect data points to their corresponding statistical significance of comparisons at the top of the plot. (**C**) RNA Scope in-situ hybridization was performed on E16.5 wild-type matched littermate and *Zic3*-null spinal cords, showing qualitatively normal dILA neuron family location and numbers. For each genotype, a column of images showing a full spinal cord section and a column from a magnified region containing example dIL family subtypes are shown, with the field of view of the second column derived from the region within the box drawn with dashed white lines. White arrows indicate examples of dIL family subtypes. Other solid and dashed white lines indicate approximate anatomical boundaries of the spinal cord.

Numbers: Spinal cord sections from the same E16.5 embryos were used for both spatial transcriptomics and in-situ hybridization: n=3 wild-type, n=3 *Zic3*-null and n=3 *Zic1/4*^{DKO} with an additional five wild-type and *Zic3*-null embryos examined for in-situ hybridization alone (n=8 wild-type, n=8 *Zic3*-null). For spatial transcriptomics, numbers of E16.5 wild-type brachial neurons are noted in Fig. S14, downsampled to 13,356 neurons of which 6,216 were classified as dIL. Additionally, cell coordinates were derived from two whole sections and one hemisection (N=2.5 sections) among n=3 replicate Zic3-null E16.5 spinal cords. From these sections 13,197 cells were identified as neurons and plotted, of which 6,753 were annotated as dIL neurons.

Statistics: (B) Two-way ANOVA (neuron subtype x genotype). For each neuron subtype in dILA2, Tukey's multiple comparisons test was performed for each pair of genotypes, the result of which is indicated by asterisks above the plot. **p<0.01, ***p<0.001

Scale bars: 250 µm, or 100 µm when magnified from boxes.

Abbreviations: ANOVA – Analysis of variance

Figure S23: Absence of *Zic1* and *Zic4* systematically alters dIL subtype composition across families

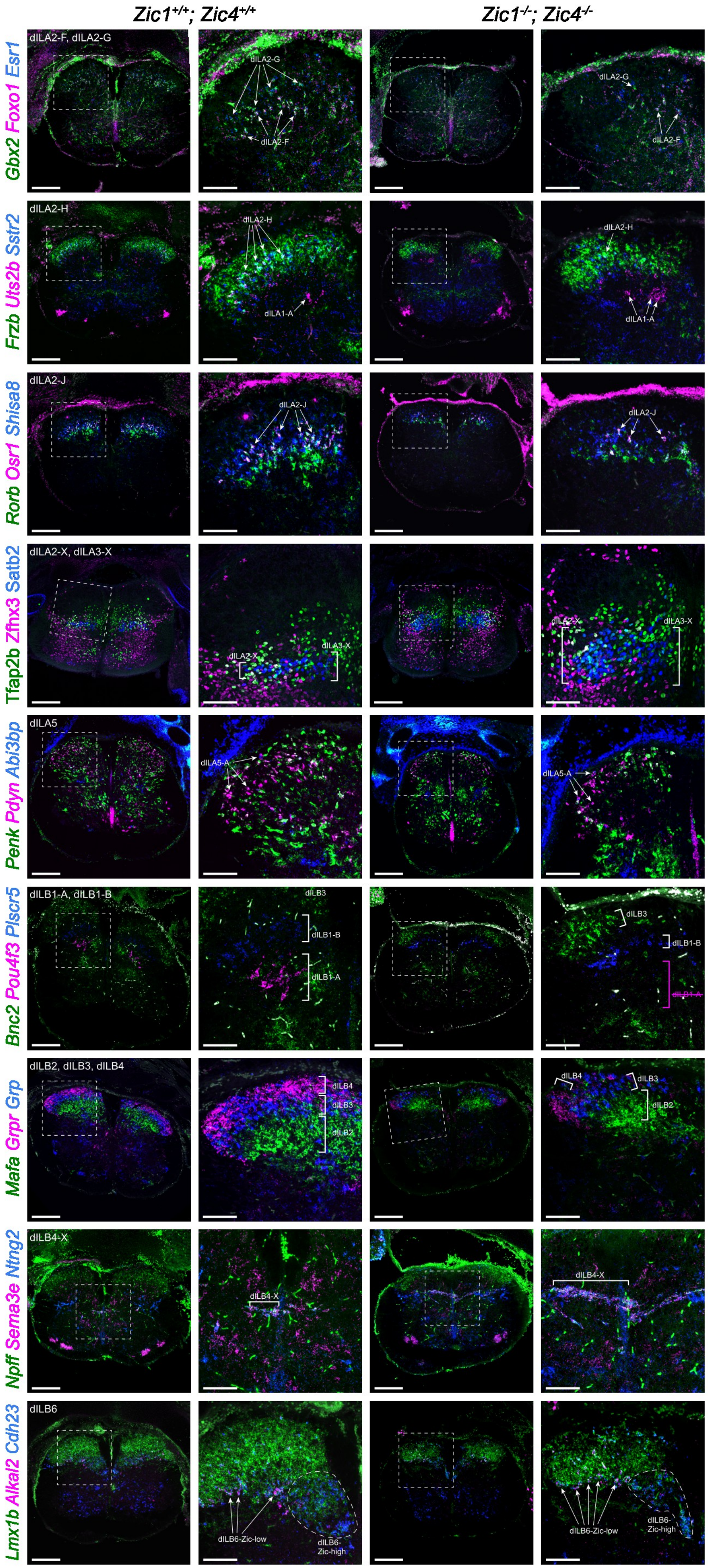

Fig. S23: Absence of *Zic1* and *Zic4* systematically alters dIL subtype composition across families.

Images of immunohistochemistry and RNA Scope in-situ hybridization of selected dILA and dILB dorsal horn neuron types (indicated in the top left of each row) are examined in E16.5 spinal cords from wild-type (columns 1 and 2) or *Zic1/4*^{DKO} embryos (columns 3 and 4). For each genotype, a low magnification images (columns 1 and 3) and a high magnification image derived from boxes marked by dashed white lines in low magnification images (columns 2 and 4) are shown. White arrows or brackets indicate examples of dIL family subtypes. Other solid and dashed white lines indicate approximate anatomical boundaries of the spinal cord. Magenta text or brackets indicates depletion of neuron types in DKO spinal cords.
Numbers: Images obtained from representative n=3 wild-type and n=3 *Zic1/4*^{DKO} embryos.
Scale bars: 250 µm for columns 1 and 3; 100 µm for columns 2 and 4.
Abbreviations: DKO – double knock-out

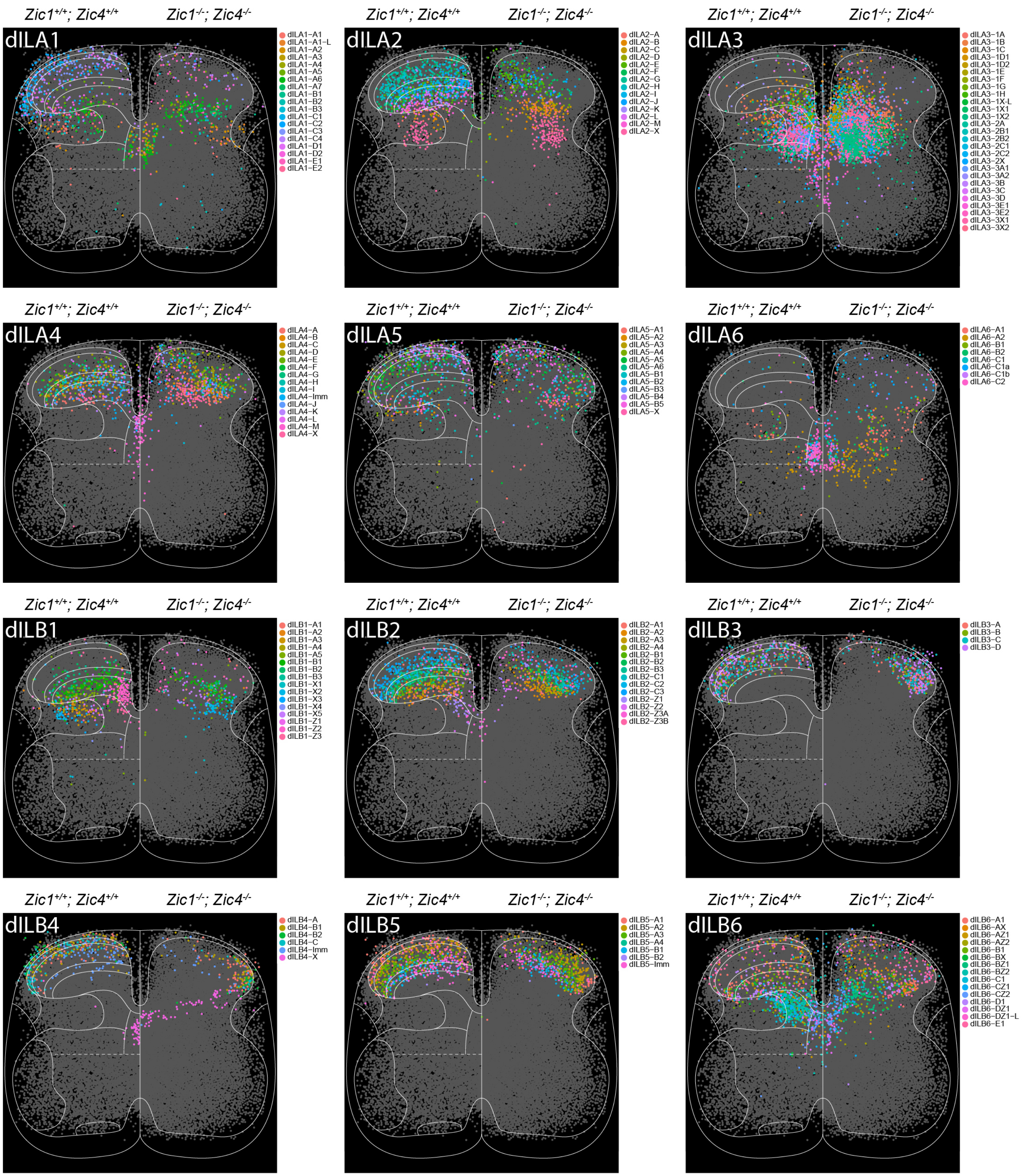
Zic1+/+; Zic4+/+
Zic1-/-; Zic4-/-
dILA1
dILA1-A1 dILA1-A1-L dILA1-A2 dILA1-A3 dILA1-A4 dILA1-A5 dILA1-A6 dILA1-A7 dILA1-B1 dILA1-B2 dILA1-B3 dILA1-C1 dILA1-C2 dILA1-C3 dILA1-C4 dILA1-D1 dILA1-D2 dILA1-E1 dILA1-E2
dILA2
dILA2-A dILA2-B dILA2-C dILA2-D dILA2-E dILA2-F dILA2-G dILA2-H dILA2-I dILA2-J dILA2-K dILA2-L dILA2-M dILA2-X
dILA3
dILA3-1A dILA3-1B dILA3-1C dILA3-1D1 dILA3-1D2 dILA3-1E dILA3-1F dILA3-1G dILA3-1H dILA3-1X-L dILA3-1X1 dILA3-1X2 dILA3-2A dILA3-2B1 dILA3-2B2 dILA3-2C1 dILA3-2C2 dILA3-2X dILA3-3A1 dILA3-3A2 dILA3-3B dILA3-3C dILA3-3D dILA3-3E1 dILA3-3E2 dILA3-3X1 dILA3-3X2
dILA4
dILA4-A dILA4-B dILA4-C dILA4-D dILA4-E dILA4-F dILA4-G dILA4-H dILA4-I dILA4-Imm dILA4-J dILA4-K dILA4-L dILA4-M dILA4-X
dILA5
dILA5-A1 dILA5-A2 dILA5-A3 dILA5-A4 dILA5-A5 dILA5-A6 dILA5-B1 dILA5-B2 dILA5-B3 dILA5-B4 dILA5-B5 dILA5-X
dILA6
dILA6-A1 dILA6-A2 dILA6-B1 dILA6-B2 dILA6-C1 dILA6-C1a dILA6-C1b dILA6-C2
dILB1
dILB1-A1 dILB1-A2 dILB1-A3 dILB1-A4 dILB1-A5 dILB1-B1 dILB1-B2 dILB1-B3 dILB1-X1 dILB1-X2 dILB1-X3 dILB1-X4 dILB1-X5 dILB1-Z1 dILB1-Z2 dILB1-Z3
dILB2
dILB2-A1 dILB2-A2 dILB2-A3 dILB2-A4 dILB2-B1 dILB2-B2 dILB2-B3 dILB2-C1 dILB2-C2 dILB2-C3 dILB2-Z1 dILB2-Z2 dILB2-Z3A dILB2-Z3B
dILB3
dILB3-A dILB3-B dILB3-C dILB3-D
dILB4
dILB4-A dILB4-B1 dILB4-B2 dILB4-C dILB4-Imm dILB4-X
dILB5
dILB5-A1 dILB5-A2 dILB5-A3 dILB5-A4 dILB5-B1 dILB5-B2 dILB5-Imm
dILB6
dILB6-A1 dILB6-AX dILB6-AZ1 dILB6-AZ2 dILB6-B1 dILB6-BX dILB6-BZ1 dILB6-BZ2 dILB6-C1 dILB6-CZ1 dILB6-CZ2 dILB6-D1 dILB6-DZ1 dILB6-DZ1-L dILB6-E1

Fig. S24: Laminar position of dIL family subtypes in *Zic1/4*^{DKO} spinal cords.

Scatter plots depict the coordinates of brachial wild-type and *Zic1/4*^{DKO} E16.5 neurons examined in the Xenium spatial transcriptomics assay, sorted into each of six dILA and six dILB families, and colored by subtype annotation. As described previously, wild-type cells are displayed on the left and null cells on the right of each diagram, and wild-type cells were downsampled to match the number of null cells. Cells not belonging to the indicated dIL family are plotted in gray. Solid and dashed white lines indicate approximate anatomical boundaries of the spinal cord.
Numbers: Spatial transcriptomics from E16.5 wild-type embryo brachial segments was previously described (Fig. S14). For E16.5 *Zic1/4*^{DKO} brachial segments, cell coordinates were derived from three whole sections and one hemisection (N=3.5 sections) among n=3 replicate spinal cords. From these sections, 17,505 cells were identified as neurons, and of which 7,376 were dIL neurons. E16.5 brachial data was downsampled to 17,347 neurons of which 8,852 were dIL neurons to match.

Table S1: Single-cell RNA-sequencing sample statistics

Table S2: Spinal cell type marker genes

Table S3: Neuron family statistics

Table S4: Marker genes of neuron families

Table S5: Centroid-centroid distance between Russ and Roome coarse annotations

Table S6: Refined neuron statistics

Table S7: Marker genes of refined dIL neuron types

Table S8: Confusion matrix for refined neuron types

Table S9: Roome to Russ refined annotations correspondence - summary

Table S10: Centroid-centroid distance between Russ and Roome refined annotations

Table S11: Pseudotime distance

Table S12: Statistical tests

Table S13: Xenium gene panel

Table S14: Top PC loadings of dIL families

Table S15: Materials list
